# Standardizing Substrate Selection: A Strategy toward Unbiased Evaluation of Reaction Generality

**DOI:** 10.1021/acscentsci.3c01638

**Published:** 2024-04-08

**Authors:** Debanjan Rana, Philipp M. Pflüger, Niklas P. Hölter, Guangying Tan, Frank Glorius

**Affiliations:** Universität Münster, Organisch-Chemisches Institut, Corrensstraße 36, 48149 Münster, Germany

## Abstract

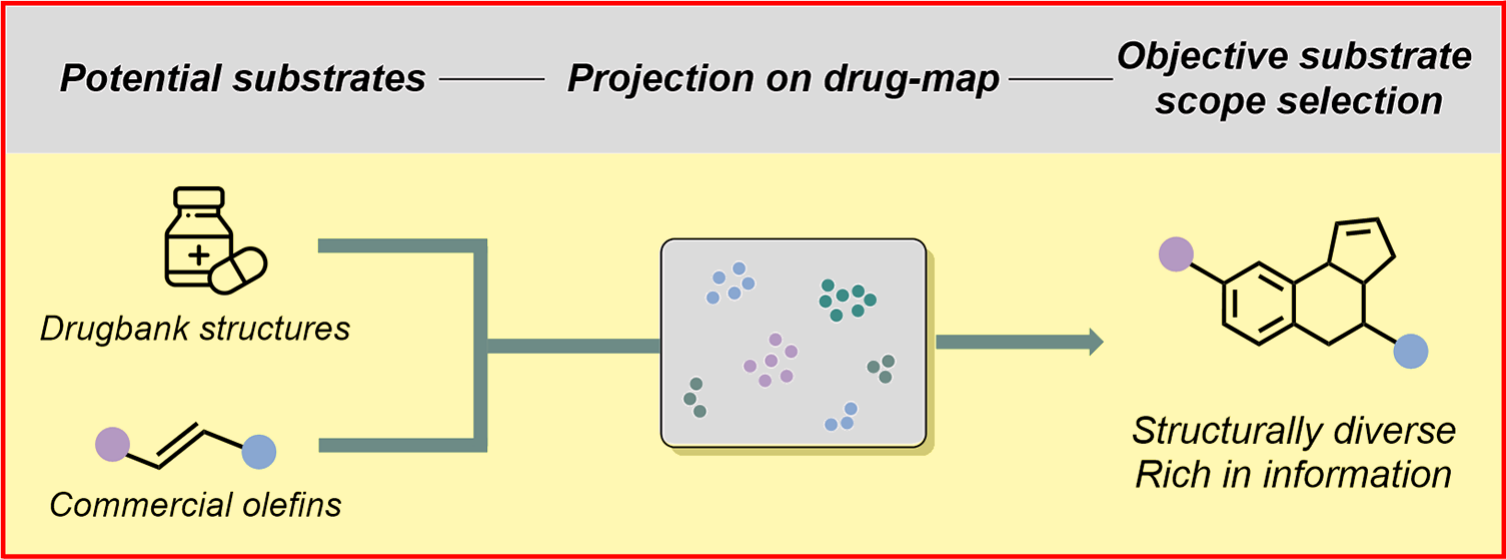

With over 10,000 new reaction protocols arising every year, only a handful of these procedures transition from academia to application. A major reason for this gap stems from the lack of comprehensive knowledge about a reaction’s scope, i.e., to which substrates the protocol can or cannot be applied. Even though chemists invest substantial effort to assess the scope of new protocols, the resulting scope tables involve significant biases, reducing their expressiveness. Herein we report a standardized substrate selection strategy designed to mitigate these biases and evaluate the applicability, as well as the limits, of any chemical reaction. Unsupervised learning is utilized to map the chemical space of industrially relevant molecules. Subsequently, potential substrate candidates are projected onto this universal map, enabling the selection of a structurally diverse set of substrates with optimal relevance and coverage. By testing our methodology on different chemical reactions, we were able to demonstrate its effectiveness in finding general reactivity trends by using a few highly representative examples. The developed methodology empowers chemists to showcase the unbiased applicability of novel methodologies, facilitating their practical applications. We hope that this work will trigger interdisciplinary discussions about biases in synthetic chemistry, leading to improved data quality.

## Abstract 2

We introduce an objective substrate scope selection method for assessing the generality of chemical reactions.

## Introduction

The synthesis of new and active compounds with ever-increasing complexity continues to be the bottleneck in pharmaceutical research.^1,2^ Therefore, developing smart synthetic methodologies and protocols that enable novel ways of making molecules efficiently drives progress in this crucial industry.^3,4^ Driven by this need, thousands of reports on synthetic methodology get published each year, and this number is only expected to rise.^5^ However, it is alarming to note that the vast majority of these reactions never find their way into industrial application.^6,7^ This becomes particularly evident by the fact that the 10 most used reactions in medicinal chemistry all originate from the last century.^7^ So what is preventing new methods from being used in industry? Reasons could lie in chemical limitations of the reactions themselves, such as scalability or functional group tolerance; however, even with these restrictions, reactions should be applicable in specific cases.^2,3^ Another explanation could be the lack of comprehensive understanding of the reaction, which limits the chemist’s confidence in its synthetic utility.^8^ Following this, integrating a new reaction into the synthetic chemist’s toolbox not only demands wide tolerance to a range of functional groups but also requires knowledge about its applicability and especially limitations.^2,9^

**Figure 1:**
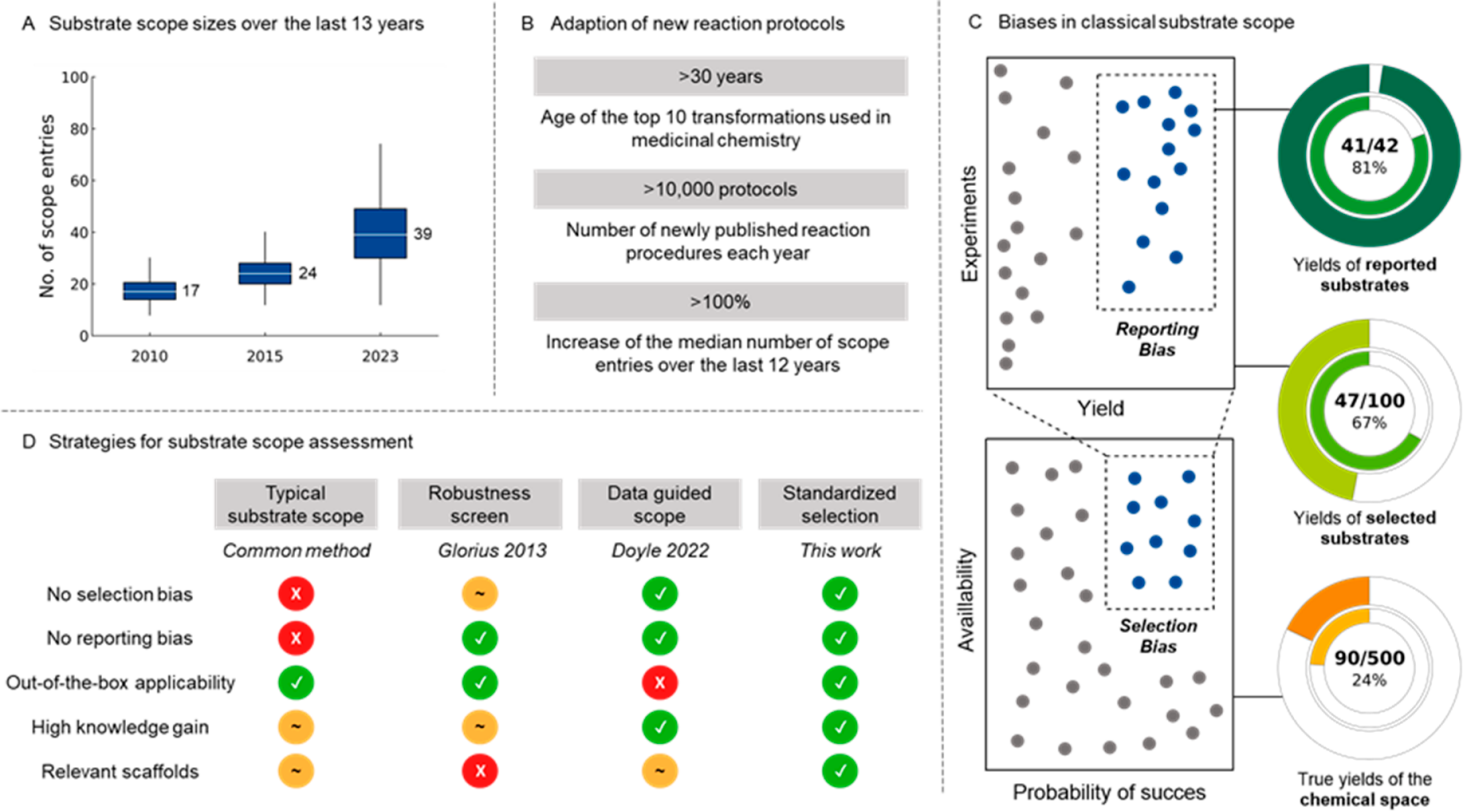
Overview of current substrate scope statistics and challenges. (A) Increase in the number of scope entries over the last 13 years (SI section 2.4). (B) Missing transition of new protocols to industry. (C) Biases involved in reported substrate scope studies. Nested pie charts depict the number of successful examples (outer circle) and average yield (inner circle). (D) Comparison of contemporary strategies for the substrate scope.

To demonstrate this tolerance and reaction generality, chemists usually test a variety of different substrates for a transformation and include the results in their publication. Thereby, reports typically showcase a range of entries from 20 to more than 100 in their scope tables. This conventional substrate scope presents entries with varying electronic and steric properties as well as substitution patterns. However, due to the combinatorial nature of chemistry, even the best scope will always be incomplete.^10^ To counteract this complexity, chemists have begun to test more and more compounds, leading to a dramatic increase in the average number of reported substrate scope entries (Figure 1A).^9^ Although this recent trend aims to emphasize the robustness of the developed protocol, current enlarged scope tables are often redundant. The underlining reason is their subjection to substantial biases,^8,11^ namely, selection^12^ and reporting bias (Figure 1C).^13^ The former—selection bias—can be explained by the chemist’s prioritization of substrates that are expected to give higher yields or are easily accessible. The latter—reporting bias—is given, as most publications do not report unsuccessful experiments or low-yielding results that constitute negative data.^14^ It should be mentioned that these biases are known to exist in all sciences.^15^ However, while measures to minimize these have been taken in several disciplines,^16^ those in synthetic chemistry are still in their nascent stage.^17^

Therefore, new approaches to standardize substrate selection and comparably benchmark reaction generality are highly desirable (Figure 1D).^18,19^ On this note, our group previously developed the robustness screen, which provides a simple way to assess the functional group tolerance of a reaction.^19^ This screen measures the impact of standardized additives on the reaction outcome and therefore allows an approximation of the applicability and limits of any given report. Although the protocol is readily applicable, it fails to capture intramolecular effects such as electronics and sterics as the additives consist of small molecules, representing synthetic needs only partially. Researchers from Merck introduced the informer library, comprising a set of structurally complex substrates specifically chosen to maximize coverage of the physicochemical drug space.^20^ While this library facilitates screening conditions for cross-coupling reactions, its applicability to other substrate classes has been limited. In addition, the utilized principal component analysis method cannot identify complex global and local structural patterns. Recently, Doyle and co-workers reported an efficient method for the scope selection of aryl bromides based on their coverage of the chemical space.^21^ Their method selects the scope broadly to cover electronic and steric effects of substituents around the reaction center based on calculated quantum chemical descriptors. While the method impressively reduces selection and reporting bias, it cannot capture the diverse reactivity and interactions of functional groups in complex pharmaceutically relevant substrates. Moreover, applying this methodology to different transformations requires significant adaptations, including defining a suitable set of quantum chemical descriptors followed by time-consuming calculations for a large dataset of substrate candidates.

Given the rapid pace at which new scientific literature is published, it is imperative to develop new approaches to meaningfully evaluate synthetic protocols based on their generality. For such a method to provide meaningful conclusions, it must meet the following requirements: I) should be low in selection and without any reporting bias; II) should be readily applicable to any chemical transformation; III) should provide broad knowledge with the minimal number of substrates; and IV) gained insights should be applicable on complex scaffolds such as those found in drugs. One solution to fulfill these requirements could be to map available substrates onto the currently known chemical space of drugs followed by an unbiased selection of a diverse set of compounds. This selected set of molecules could be tested in addition to the conventional scope examples, therefore providing a comparable benchmark of the protocol’s applicability. Based on this hypothesis, we herein report a standardized approach for substrate selection that can be easily applied by researchers to test the unbiased applicability of their developed methods.

## Results and Discussion

**Figure 2:**
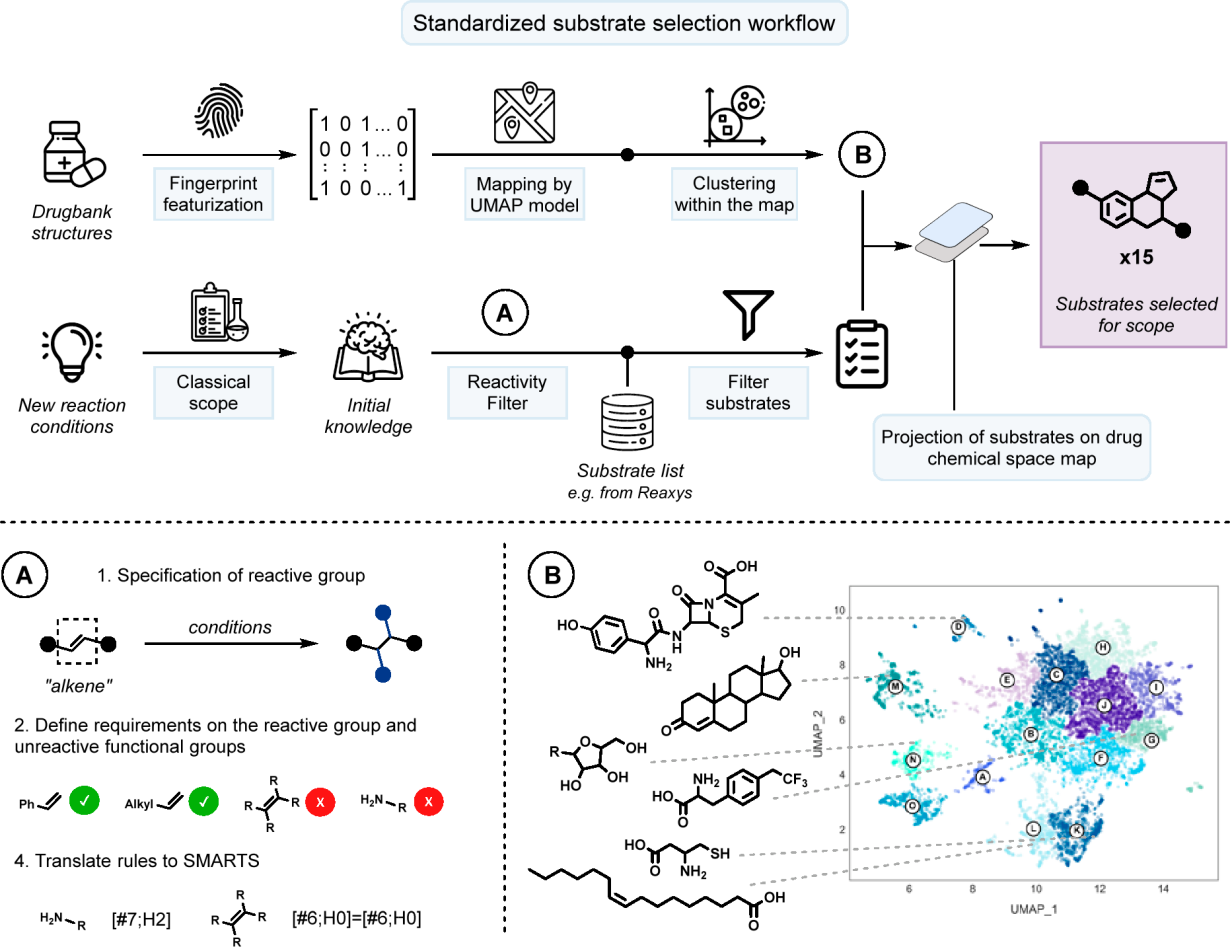
Schematic overview of steps involved in the standardized substrate selection workflow. (A) Analyzing reaction compatibility and requirements from the initial classical scope for filtering the potential list of substrates. (B) Mapped chemical space of drugs obtained after UMAP dimensionality reduction and hierarchal clustering; cluster centers are labeled from A to O.

The envisioned substrate selection workflow operates in three steps: first, a machine learning algorithm is utilized to identify common structural patterns inherent to the given molecular dataset such as a drug library. Thereby, it maps molecules sharing similar scaffolds closer together while placing structurally dissimilar structures further apart from each other. This map can be divided into clusters and is then utilized in the second step: the trained machine learning model analyzes potential reaction substrates based on their structural proximity to previously given drug scaffolds and projects them onto the original map. These overlaid maps are then used in a third step to finally select candidate molecules for experimental reaction evaluation (Figure 2).

To begin the development of this workflow, we opted for the Drugbank database as a representative dataset encompassing the structural diversity of drug molecules.^22^ It is worth noting that this approach could also be applied to datasets for other applications such as crop protectants or fragrances.^23^ For the sake of clarity, it has to be stated that while our aim is to minimize the human bias in substrate selection, the choice of Drugbank here introduces a dataset bias. However, this choice was motivated by the significant focus within the field of synthetic methodologies on streamlining the synthesis of pharmaceutical compounds. Next, we featurized the drug molecules utilizing extended connectivity fingerprints (ECFP).^24^ While quantum chemical descriptors have been shown to be effective in mapping molecular structures,^21^ they feature major disadvantages for the given application. Typically, they are highly problem- and structure-specific,^25^ lacking a general set of descriptors to describe the high structural diversity of the drug chemical space. In contrast, molecular fingerprints can natively encode substructures,^24^ providing a robust structural representation designed for broader applicability. (See SI section 5 for fingerprint comparison.)

With a general molecular featurization in hand, we turned our focus toward the mapping of the drug chemical space,^26,27^ leveraging unsupervised learning, which has already demonstrated promising results in identifying inherent patterns in molecular datasets.^26,28^ To obtain a meaningful map of the drug chemical space, structural relationships and similarities between drug molecules need to be identified.^29^ To achieve this, we employed UMAP (Uniform Manifold Approximation and Projection), a nonlinear dimensionality reduction algorithm utilized for embedding chemical datasets.^30^ The amount of global information (such as recurring motifs, e.g. terpenes,^31^ or sugar-like patterns^32^) versus the local information (such as functional groups or substitutional variations) captured by UMAP is dependent on two key parameters: the minimum distance allowed between data points (*M*_*d*_) and the number of nearest neighbors (*N*_*b*_). To optimize these parameters, two metrics were utilized: the correlation of the Jaccard distance between fingerprint pairs and their distance in the final projection (*D*), as well as the silhouette score^33^ (*S*) to assess whether significant clustering can be achieved in the projected space (SI section 4). It was found that by using a number of nearest neighbors *N*_*b*_ = 30 and a minimum distance of *M*_*d*_ = 0.1, a mapping can be accomplished which effectively preserves global similarity while still capturing distinct local characteristics of specific compound classes (Figure 2B).

Subsequently, we performed clustering to compartmentalize the embedded drug chemical space. Evaluating different clustering algorithms revealed hierarchical agglomerative clustering^34^ as the superior method (SI section 6). The algorithm conserves visually separable clusters while segmenting larger regions. However, determining the number of clusters poses a significant challenge, as this would then determine the size of a later scope. As any example could in principle give new information, we argue that a scope of more than 25 examples would be impractical. This is especially true if complex substrates are tested. In contrast, a scope of fewer than 10 examples would neglect relevant structural motifs. To select an appropriate number, we computed the silhouette scores for cluster numbers ranging from 10 to 25, revealing no significant trend. Ultimately, we chose 15 clusters for practical reasons, allowing us to adapt the methodology efficiently. It has to be stated, however, that a greater number could also be selected.

The UMAP embedded drug chemical space forms the basis of the envisioned substrate selection workflow. In the subsequent step, the trained UMAP model can be utilized to project any given class of substrate molecules onto the drug map and finally select candidate molecules for the substrate scope. This capability that different substrate classes could be projected onto the universal drug map makes our approach generally applicable to various substrate categories. Depending on the reaction, initially a broad list of molecules for a specific substrate class should be collected from a molecular database or supplier catalogue and filtered based on previous knowledge of reactivity. The filtering process allows for explicitly stating already known limitations and ultimately enhances information about the reactivity. These filters can typically be obtained from the conventional scope of a reaction’s incompatibility toward specific functional groups or steric restrictions (Figure 2A). Complementary unbiased data-driven approaches such as the quantum chemically modeled substrate selection^21^ could also be utilized for identifying electronic and steric reactivity trends and defining filters.

The filtered list is then fed into the trained UMAP model, which can project these possible starting materials on the drug map. Within this process, the previously learned interdependencies are utilized by the model to project substrates based on their similarity to known drugs. To select a final list of diverse scope entries from this projection, previously derived clusters are utilized. Thereby, the substrates which fall in closest proximity to each drug cluster center are chosen and subjected to reaction conditions. In cases where the centermost candidate is hardly accessible (e.g., due to price or availability concerns), the top-*n* closest structures should be considered. The entire standardized substrate selection workflow has been automated through a web interface,^35^ making it readily accessible to synthetic chemists. Users only need to upload a list of potential substrate molecules for obtaining a standardized set of substrates. (See SI section 3.1 for implementation guidelines.)

**Figure 3:**
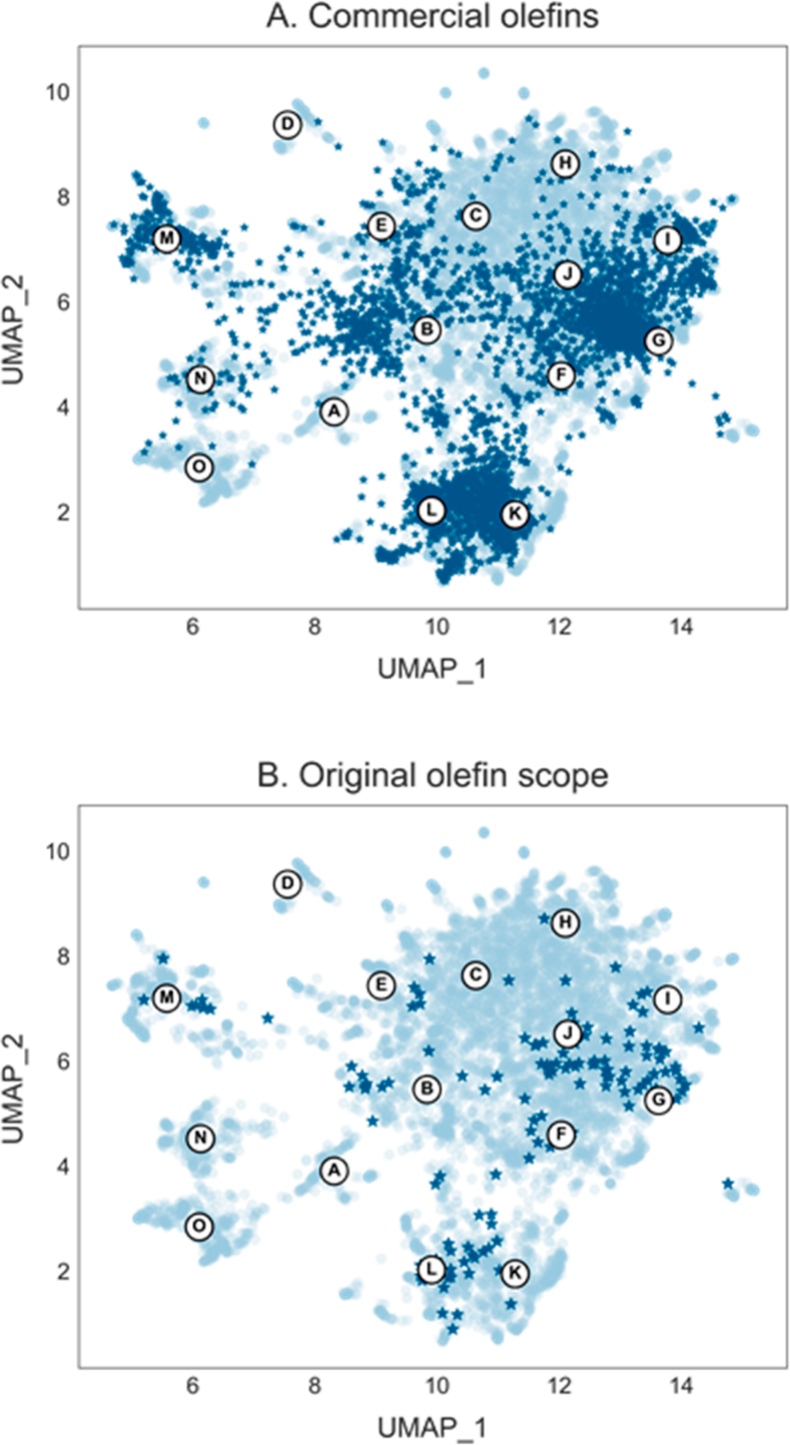
Projections of olefins (dark blue) over the drug chemical space (light blue). (A) Commercially available olefins were filtered from Reaxys. Olefins lying closest to the center of each drug cluster are selected for the standardized substrate scope. (B) Olefins shown in the original photocatalytic iminocarboxylation report.

To test our standardized substrate selection strategy, we chose two reactions. First, the photochemical iminocarboxylation of alkenes, recently reported from our laboratory.^36^ This energy transfer enabled difunctionalization approach converts alkenes to biologically important β-amino acid derivatives in a single step. Previously, the reaction was tested on 100 different alkene substrates and was able to tolerate a broad variety of functional groups. However, as is true for most reaction procedures published today, the presented scope was subjected to reporting and selection bias, making this reaction ideal for our method. Second, to compare the results against the same set of substrates, we also chose the osmium-catalyzed dihydroxylation of alkenes.^37,38^ This reaction is a widely known transformation and is routinely used within academic and industrial chemical laboratories all over the world due to its versatility. For defining the broadest possible substrate space for the alkenes, the Reaxys database was queried on all the commercially available olefins. To keep costs acceptable and the approach applicable, the results were filtered based on price (<100 €/g) and molecular weight (<700 Da). In addition, tetra-substituted alkenes and free amine groups were filtered out because they were known to be incompatible with the photochemical iminocarboxylation reaction. Overall, a dataset of 3811 olefins was obtained and projected onto the drug map by the trained UMAP model (Figure 3A).

Alkenes typically do not share the same scaffold complexity as drugs, and as a result, certain regions (e.g., linear chains) have a higher density compared to others. This nonuniform distribution again reemphasizes the need for a systematic approach, as random or human selection would result in substrates being picked primarily from the denser regions, thus giving less information about a protocol’s applicability (Figure 3B). Therefore, based on our selection strategy, alkenes lying closest to the center of each drug cluster were chosen based on availability, yielding a representative set of alkenes. As expected, the selected set of 15 candidate molecules represents a wide variety of alkenes, including terminal, 1,1-disubstituted, 1,2-disubstituted, and trisubstituted alkenes. Alkenes with different electronics ranging from electron-deficient α,β-unsaturated ketones, unactivated terminal alkenes, and electron-rich conjugated ethers are present. Various functional groups such as alcohols, esters, amides, ethers, silyl ethers, halides, thiols, tertiary amines, and trifluoroethyl groups are also included. Notably, in comparison to conventional scopes, a major fraction of the selected molecules are polyfunctionalized. To demonstrate that the developed standardized scope selection workflow is highly transferable and applicable to different classes of compounds, it was also tested with (hetero) aryl bromides, again showing uneven but broad drug space coverage. Following the described selection process, it was possible to obtain a diverse set of (hetero) aryl bromides (SI section 8).

**Figure 4:**
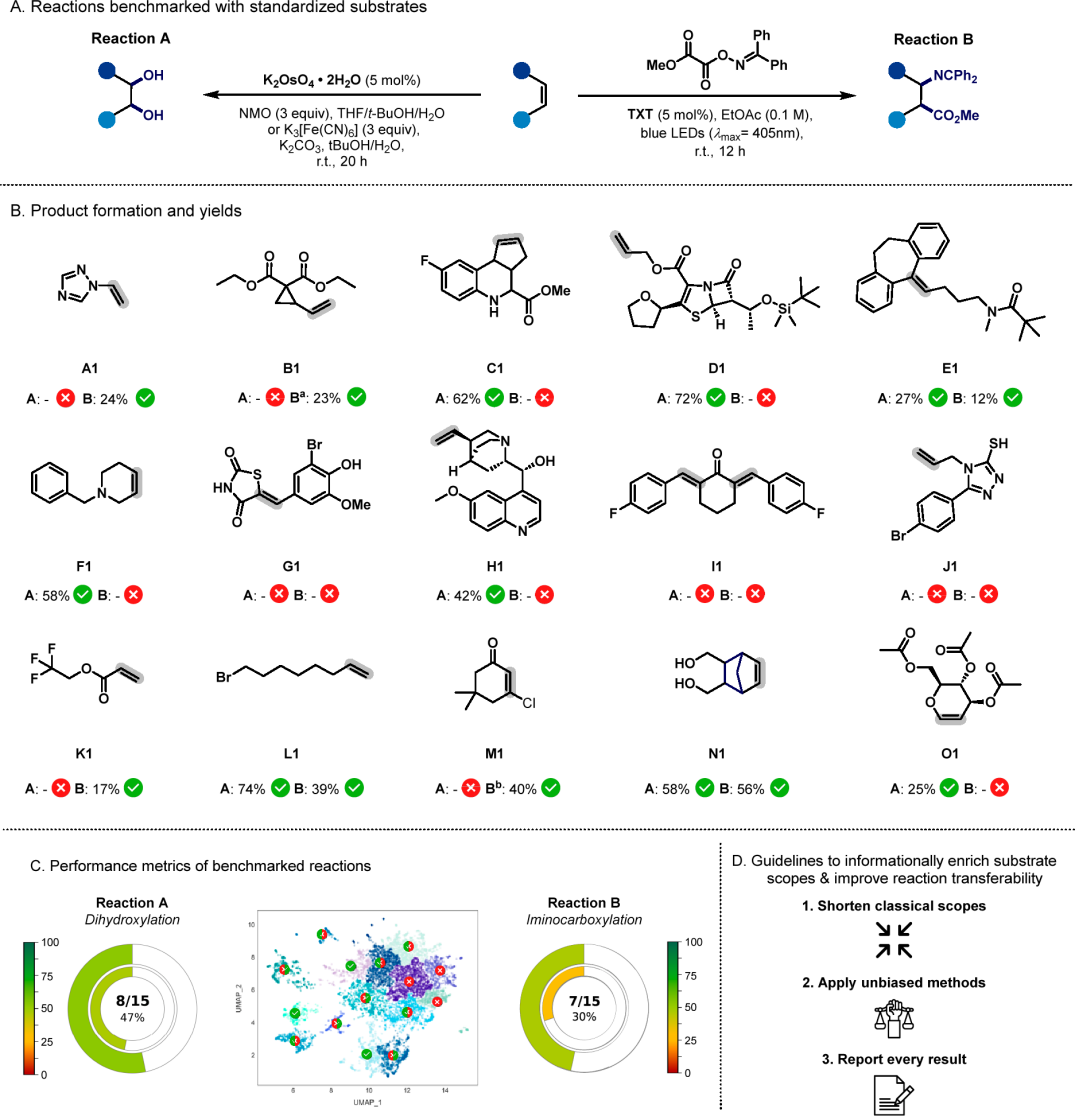
(A) Experimental reaction evaluation with the 15 alkenes selected by the standardized substrate selection workflow. (B) The alkenes are labeled according to the cluster labels (A1 to O1). All reactions were performed at 0.2 mmol scale, and isolated yields are reported. Full experimental procedure can be found in the Supporting Information. ^a^Ring-opening product was obtained. ^b^Dechlorinated and β-hydrogen eliminated product was obtained. (C) Performance metrics of the benchmarked reactions. Nested pie charts: number of working substrates (outer ring) and average yield of successful reactions (inner ring). (D) Guidelines to improve the reaction evaluation and applicability of protocols.

While the substrate selection process can be applied to most reactions, we focused our experimental evaluation on the 2 aforementioned reactions with all 15 selected olefins being subjected to both reaction conditions (Figure 4). Our criterion for a reaction to be successful was obtaining the expected product in greater than 10% yield (isolable). This threshold is arguably low, although it provides enough product for analytical as well as primary pharmaceutical studies. Following this criterion, eight (53%) substrates successfully underwent dihydroxylation and seven (47%) substrates underwent iminocarboxylation. Unactivated alkenes (L1 and N1) were successfully converted in both reactions. The nortriptyline derivative (E1) was also converted in both reactions, albeit in lower yields. 1-Vinyltriazole (A1), vinyl cyclopropane derivative (B1), and electron-deficient alkenes M1 and trifluoroethyl acrylate (K1) were unreactive toward dihydroxylation but yielded the corresponding β-amino acid derivatives. Despite participating in the desired iminocarboxylation reaction, alkene-M1 underwent dechlorination and subsequent β-hydrogen elimination, and alkene-B1 afforded the ring-opening product under the employed photochemical conditions. Three out of the 15 chosen alkenes (G1, I1, and J1) were unsuccessful in both reactions. However, a tetrahydropyridine derivative (F1), a β-lactam-bearing alkene (D1), (−)-quinuclidine (H1), triacetyl-d-glucal (O1), and alkene-C1 were successfully converted to their corresponding diols, while the expected products were not detected in the case of iminocarboxylation (Figure 4B).

Given the relatively small number of examples presented in this standardized scope, the information obtained from this set of structurally diverse substrates is manifold. For example, in the iminocarboxylation reaction, the positive scope outcomes align with the originally published scope, focusing on styrenes, unactivated alkenes, and electron-deficient alkenes. However, the iminocarboxylation shows limited applicability when it comes to structurally complex substrates bearing multiple reactive functionalities, which contradicts the originally published scope that exemplified complex substrates where the olefin was located farther away from the reactive functionalities.^36^ In contrast, the osmium-catalyzed dihydroxylation reaction, being a long-standing valuable tool in synthetic chemistry, exhibits a broader scope. The dihydroxylation reaction successfully converted 8 out of the 15 substrates, including structurally complex alkenes. Substrates that did not work include electron-deficient and heteroatom-bound alkenes, which are well-known limitations for this reaction.^37^ Overall, the osmium-catalyzed dihydroxylation demonstrated greater productivity with a 47% average yield as compared to 30% for the iminocarboxylation reaction (Figure 4C). The structural, electronic, and functional diversity and the substrate complexity underline the potential of the developed approach to serve as a benchmark for reaction applicability.

While a conventional substrate scope approach remains crucial for assessing electronic and steric demands, we advocate against enlarging them with human-selected examples for showcasing reaction generality. Instead, we propose a two-step approach: first, performing a short classical scope to identify reactivity trends and second, combining it with examples selected by the standardized substrate selection workflow. This combined approach will enrich the information in substrate scope tables and facilitate a more unbiased evaluation of reaction generality (Figure 4D). It is also important to note that the standardized scope selection approach presents a considerable fraction of negative (i.e., low/no-yielding) examples. Although the inclusion of low-yielding or unsuccessful scope entries can be observed occasionally in present day publications, still a large number of unsuccessful scope examples remain unpublished.^9,14^ This originates from researchers being hesitant to share unsuccessful examples, which might negatively impact their chances of publication. We must state clearly that negative results are equally important in delineating the limits of applicability of a newly reported reaction and identifying potential room for further methodological improvements. Therefore, we encourage the scientific community to share, accept, and even highlight negative results.

## Conclusions

We have developed a standardized substrate selection strategy allowing for a straightforward, unbiased, and comparable assessment of chemical reactions. At the core of our workflow, a UMAP model generates a map of a chemical (e.g., drug space) by learning underlying structural relationships and projecting reaction substrates onto the chemical space based on structural similarity. Selecting substrates from the different clusters in the drug map enables an unbiased selection of a diverse set of representative examples. Our strategy also complements previous unsupervised learning based substrate selection approaches which focused on quantum chemical modeling of the reactive center.^21^ We have demonstrated the applicability of the workflow on different substrate classes, namely, alkenes and (hetero)aryl bromides, and utilized it to assess the scope of different chemical reactions.

Since 15 substrates comprise a small fraction as compared to the full chemical space of a substrate class, the perception of reaction generality on the entire chemical space still remains limited, especially considering the fact that a minute structural variation can have major consequences on the reaction outcome. As variations in UMAP or clustering parameters can lead to a set of 15 similar but yet different substrates, the chosen centermost substrate may not always explain the complete reactivity of the whole cluster. However, due to the underlying complexity of chemical reactions, we anticipate that by sampling only a few molecules no individual method will ever be able to capture all reactivity trends. With that said, the current trends of increasing scope entries, which are often biased and redundant, could be enriched in information by a standardized approach to diverse substrate selection. Therefore, we anticipate our workflow to be used as a tool toward an unbiased scope evaluation of new chemical transformations.

To further make this approach user-friendly, a web platform^35^ was built for generating representative substrate scopes and visualization of the given substrate space using only a list of potential substrate SMILES. Alternatively, it can also be used to retrospectively evaluate already existing substrate scope tables. To demonstrate the broad applicability of a reaction and enable quick adoption in academia and industry, a scope does not necessarily need to be enlarged but broadened in diversity and cleansed of biases. Implementing our proposed substrate selection strategy in addition to the conventional scope examples would inevitably reveal a more realistic picture of a new transformation’s true utility.

## Data Availability

The web interface can be accessed through the link https://pharmascope.uni-muenster.de/. The developed code and all associated datasets can be found at https://zivgitlab.uni-muenster.de/ag-glorius/published-paper/standardizing-substrate-selection/.

## Supporting Information Available

Details of python scripts and implementation guidelines for the developed workflow, supporting experiments, details of synthetic procedures, and data of isolated compounds (PDF)
