# Supplementary material for: Standardizing Substrate Selection: A Strategy toward Unbiased Evaluation of Reaction Generality
Source: ACS Cent Sci. 2024 Apr 8;10(4):899–906. doi: 10.1021/acscentsci.3c01638 (PMC11046462; doi:10.1021/acscentsci.3c01638)
Supplement: Supplementary file 1 — oc3c01638_si_001.pdf [file oc3c01638_si_001.pdf]

Supplementary Information

Standardizing Substrate Selection – A Strategy Towards Unbiased Evaluation of Reaction Generality

Debanjan Rana, Philipp M. Pflüger, Niklas P. Hölter, Guangying Tan, Frank Glorius*

Organisch-Chemisches Institut, Universität Münster, Corrensstraße 36, 48149 Münster.

*Corresponding author. Email: glorius@uni-muenster.de

Table of Contents

1. General Information *S3*

1.1 Reagents, Solvents and experimental conditions S3

1.2 Computational hardware information S3

1.3 Analytical Techniques S3

1.4 Photochemical Reaction Setup S4

1.5 Compound Purification S4

2. Data Pre-processing *S5*

2.1 Data pre-processing for Drug Bank S5

2.2 Data pre-processing for Olefins S5

2.3 Data preprocessing for aryl bromides and heteroaryl bromides S7

2.4 Substrate scope statistics S7

3. Standardized substrate selection workflow – implementation guidelines *S8*

3.1 Guidelines for using the Web-Interface S10

3.2 Details of Python Scripts S13

4. UMAP parameters optimization *S15*

5. Comparative experiments for molecular fingerprints *S18*

6. Clustering UMAP embeddings *S20*

7. Different substrate selection strategies *S23*

8. Application to other substrate classes *S31*

9. Experimental details *S34*

9.1 General procedure for photocatalytic imino-carboxylation S34

9.2 General procedure for osmium-catalyzed dihydroxylation S34

10. Experimental data for the synthesized products *S36*

11. Copies of ^{1}H and ^{13}C NMR *S45*

12. References *S62*

1. General Information

1.1 Reagents, Solvents and experimental conditions

Unless otherwise stated, all reactions were performed in oven-dried glassware under argon atmosphere, using standard Schlenk techniques. All reagents were purchased from BLD Pharm, Alfa Aesar, Sigma-Aldrich, Merck, TCI, Fluorochem, Combi-blocks, ACROS Organics and used without further purification. All solvents used in synthesis were purchased from Acros in AcroSeal® bottles and were directly stored under 3 or 4 Å molecular sieves, replacing the collected volume with argon or purified by solvent purification system (SPS). Solvents for chromatographic purification (n-pentane, CH_2Cl_2 and EtOAc) were purchased as technical grade and purified by atmospheric pressure distillation. Reaction temperatures are referred to the ones of the heating/cooling media, unless otherwise stated.

1.2 Computational hardware information

All computations were performed using 2*intel Xeon Gold 6240 CPU @ 2.60 GHz CPU cluster nodes. The associated Python scripts and related materials can be found as a zip file and also at https://zivgitlab.uni-muenster.de/ag-glorius/published-paper/standardizing-substrate-selection.

1.3 Analytical Techniques

NMR spectra were recorded at room temperature on Bruker Avance II 300, Avance II 400, Bruker Avance Neo 400, Agilent DD2 500 or DD2 600 spectrometers. Deuterated solvents were purchased from Euristop (CDCl3, deuteration > 99.8%) or Aldrich (CD_3OD, deuteration > 99.9 %). Chemical shifts (δ) for ^1H and ^{13}C chemicals shifts are quoted in parts per million (ppm) relative to tetramethylsilane (TMS, δ = 0.00 ppm) and were internally referenced to residual $CDCl_3$ (7.26 ppm for ^1H, 77.16 ppm for ^{13}C) or CD_3OD (3.31 ppm for ^1H, 49.00 ppm for ^{13}C). ^{19}F chemicals shifts (δ) are quoted in parts per million (ppm) and were calibrated using absolute referencing to the 1H NMR spectrum. Coupling constants (J) are reported in Hertz (Hz) to the nearest 0.1 Hz. The multiplicity of all signals were described following standard abbreviations: s = singlet, d = doublet, t = triplet, q = quartet, quintet, p = pentet, m = multiplet. All NMR spectra were processed using MestReNova 14 using standard phase and baseline correction.

High-resolution mass spectra (HRMS) were obtained by the MS service of the Organisch-Chemisches Institut, Westfälische Wilhelms Universität Münster, using electrospray ionisation (ESI) on a Bruker Daltonics, MicroToF spectrometer.

GC-MS spectra were recorded on an Agilent Technologies 7890A GC-system with an Agilent 5975C VL MSD or an Agilent 5975 inert Mass Selective Detector (EI) and a HP-5MS column (0.25 mm × 30 m, film: 0.25 µm). GC-MS samples were filtered over a plug of silica or basified alumina and eluted with EtOAc prior to analysis or were directly collected from the eluted section from column chromatography on silica gel.

Thin layer chromatography was carried out on Merck silica gel 60 F254 pre-coated aluminium sheets. TLC plates were visualized using UV light (254 nm or 365 nm) and/or stained with basic aqueous potassium permanganate.

1.4 Photochemical Reaction Setup

The following set-up was used for performing the photochemical reaction (0.2 mmol scale): the Hepatochem EvoluChem^{TM} PhotoRedOx Box Duo device (HCK1006-01023) (Fig. S1) was irradiated with two EvoluChem^{TM} Blue LEDs (18 W, (λ_{max} = 405 nm, HCK1012-01-010). The reaction temperature was measured not to be exceeding room temperature by more than 9 °C, using this setup. Stirring was ensured by magnetic stirring plates (1000 rpm). A 10 mL Schlenk tube equipped with a PTFE-screw cap and a PTFE-coated rare-earth “extra power” oval stirring bar (10 x 5 mm) was used for small scale reactions.

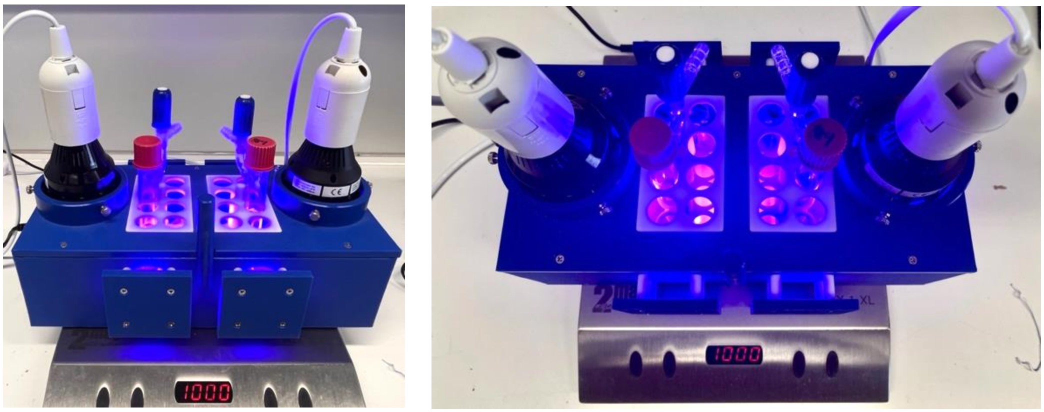

Figure S1: Hepatochem EvoluChem^{TM} PhotoRedOx Box Duo irradiated by EvoluChem^{TM} HCK 1012-01-010 blue LEDs (18 W, λ_{max} = 405 nm).

1.5 Compound Purification

Flash chromatography was carried out using silica gel (Acros Organics, 0.035-0.070 mm, 60 Å) under a light positive pressure of argon, eluting with the specified solvent system as mentioned.

2. Data Pre-processing

2.1 Data pre-processing for Drug Bank

DrugBank 5.0 was used as the reference dataset for drugs.[1] This dataset originally contained 11912 drug molecules. All molecules with valid SMILES (simplified molecular-input line-entry system)[2] were checked for RDkit parsing errors and canonicalized. Since the focus of the developed methodology was on small molecule drugs, additional filters for compounds having a molecular weight < 750 Da were applied, and inorganic compounds were excluded. This was followed by cleaning for heavy metals and salt counterparts. Finally, the dataset was checked for duplicates. After the mentioned pre-processing steps, the dataset consists of 9765 small-molecule drugs.

2.2 Data pre-processing for Olefins

For investigating the generality of the photochemical amino-carboxylation reaction,[3] we sought to build a dataset for olefins with feasibly broadest chemical space coverage. For obtaining this dataset, a Reaxys © structure query search was performed in May 2022 for commercially available olefins with prices below 100 €/g and having spectral characterization data information.

Exported data points: 70476.

The raw exported data from Reaxys © database[4] was found to be containing many erroneous entries and duplicates, so additional filtering was applied as follows:

- RDkit parsing error, canonicalization
- substructure search for aliphatic alkenes
- removing heavy metals and cleaning for salts
- removing duplicates
- Functional group filters:
    - Free amines
    - Tetra substituted alkenes
- Less than 700 Da

Data points after filtering: 3811

The described data extraction and filtering methods were implemented based on olefins, the substrate of our choice. Similar approaches could be utilized to extract data for other substrate classes. The photochemical difunctionalization reaction is incompatible with free amines and tetra-substituted alkenes, so they were removed. Depending on the reaction being investigated, the functional group filtering process should be modified. One general comment would be not to constrain the substrates with too many functional group restrictions, which would reduce the chemical space that the reaction can access. This would subsequently restrict the accessible pharmaceutical chemical space and the reaction generality.

The Python scripts (https://zivgitlab.uni-muenster.de/ag-glorius/published-paper/standardizing-substrate-selection) for preprocessing data obtained from Reaxys can be found in the file filter_substrates.py. The path of the .csv file containing the list of SMILES is provided as the input and all the preprocessing/ filtering steps can be executed by instantiating the Class *Clean_Smiles.* The description of the code is provided below. Depending on the reaction compatibility, SMARTS (SMILES arbitrary target specification)[5] should be defined to include/ exclude substrates with desired substructures. The preprocessing script can be adapted for other substrate classes by modifying the SMARTS pattern.

```
# Specify the path of the raw exported file containing the list of substrate SMILES.
path = 'data/exported_alkenes.csv'

# Instantiate the Clean_Smiles class. SMILES are canonicalized and checked
# for parsing errors.
smiles_preprocessor = Clean_Smiles(path)

# Perform a substructure search for alkenes from the substrate list by calling the method:
# screen_pattern. Specify SMARTS filter e.g., SMARTS for alkenes: '[#6]=[#6]'.
alkenes_filtered_df=smiles_preprocessor.screen_pattern(smiles_preprocessor.cannonical_s
miles_df, '[#6]=[#6]', type='include')

# Filter out salts by calling the method: cleaning_salts
salts_filtered_df = smiles_preprocessor.cleaning_salts(alkenes_filtered_df, '[#6]=[#6]')

# Filter out duplicates
duplicates_filtered_df = smiles_preprocessor.remove_duplicates(salts_filtered_df)

# Remove substrate molecules above 700 Da.
mw_wt_filtered_df = smiles_preprocessor.clean_carbon_molwt(duplicates_filtered_df,
n_Carbon=1, l_mol_wt=0, u_mol_wt=700)

# Filter out free primary amines by calling the screen_pattern class and specifying the
# corresponding SMARTS: '[NH2]~*'.
fg_filtered_df1 = smiles_preprocessor.screen_pattern(mw_wt_filtered_df, '[NH2]~*',
type='exclude')

# Define SMARTS for 1,1-disubstituted, terminal, 1,2- disubstituted and tri-substituted
# aliphatic alkenes.
patterns = ['[CH2]=[CH0]', '[CH2]=[CH1]', '[CH1]=[CH1]', '[CH0]=[CH1]']

# Perform substructure search to include the defined alkene substitution pattern by calling #
the screen_pattern method.
Final_alkenes = smiles_preprocessor.screen_pattern(fg_filtered_df1, patterns, type=’multi’)

# Save the results
final_alkenes.to_csv('data/ final_alkenes.csv', index=False)
```

2.3 Data preprocessing for aryl bromides and heteroaryl bromides

To demonstrate the transferability of the developed method on other substrate classes, we curated a dataset of commercially available aryl- and heteroaryl bromides as they represent important substrates for many reaction types, especially cross-coupling reactions.

Heteroaryl bromides were retrieved from the Reaxys © commercial substances database in March 2023 by querying the "HAr-Br" building block. The initial weight filter was set to 1000 Da. This way, an initial dataset of 73956 commercially available heteroaryl bromides was obtained.

In the same way, aryl bromides were queried with the "Ar-Br" building block and the same filters from the Reaxys © database. This yielded an initial dataset of 119995 aryl bromides.

Since we suspected that prices in the Reaxys © database are not always up-to-date and may be unreliable, e.g., for not-in-stock compounds, we applied no price filter and used the number of reports of a compound in literature & patents as a gauge for availability in a subsequent Python filtering step (see below).

Both datasets were respectively filtered individually and combined according to the following criteria using a Python script (refer to https://zivgitlab.uni-muenster.de/ag-glorius/published-paper/standardizing-substrate-selection):

- Removal of entries that were not parsable with the RDKit library in Python.
- Canonicalization of all SMILES for duplicate filtering.
- Removal of duplicates.
- Removal of compounds with more than one reactive bromide.
- Removal of compounds with M > 750 Da.
- Removal of compounds that have been reported less than 10 times in academic literature & patents. This was used instead of a price filter due to the previously mentioned reasons. Increasing this threshold will also increase the "popularity" of compounds in this dataset.

Applying these filter criteria resulted in cleaned datasets of 7222 heteroaryl bromides, 10060 aryl bromides and a combined dataset of 17282 (hetero)aryl bromides.

2.4 Substrate scope statistics

We considered the Journal of the American Chemical Society and Organic Letters for analyzing the trend of substrate scope sizes over the years (2010, 2015 and 2023). For all the issues in a year, we selected one report per issue for assessing the distribution of the number of substrate scope entries (Figure 1A).

3. Standardized substrate selection workflow – implementation guidelines

The goal of the developed workflow is not to entirely replace the conventional approach to substrate scopes but rather to eliminate biases in substrate scope assessment and diversify substrate selection.[6,7] Classical substrate scopes continue to play a pivotal role in systematically evaluating factors such as functional group tolerance, as well as steric and electronic effects. However, the recent trend of presenting an increasing number of scope entries for demonstrating reaction generality should be counteracted.[7] Instead of expanding the substrate scope analysis with human-based substrate selection, a two-step approach is proposed. Initially, a concise classical scope is performed to identify reaction compatibility and structural restrictions, followed by utilization of the standardized substrate selection workflow to unbiasedly evaluate the generality across a structurally diverse set of substrates, thereby presenting an informationally rich substrate scope.

To implement the developed standardized substrate selection approach, the following steps can be followed:

1. Defining functional group compatibility/ structural restrictions:
   Conduct a concise classical scope to determine functional group tolerance, as well as steric and electronic demands of the reaction. Complimentary substrate selection methods such as the quantum chemically modelled unbiased substrate selection can be an ideal choice for identifying electronic and steric requirements.[8] The robustness screen can also be utilized for assessing functional group tolerance.[9]

2. Compiling a broad list of potential substrates:
   Curate a broad list of molecules for the desired substrate class from commercial substrate catalogues, databases, or inventories. The goal should be to keep this list as broad and diverse as possible.
   e.g., querying Reaxys © database[4] for commercially available compounds with substrate SMARTS pattern.

3. Filtering substrates based on the reactivity knowledge:
   Filter the obtained substrate list based on the gathered reactivity knowledge. The SMARTS language[5] should be employed to perform substructure search and exclude functional groups or substitution patterns that are found to be reaction incompatible. The SMARTS language allows for defining a clear rule which helps to inform future users of the reaction about its applicability.
   e.g., primary free amines ([NH2]~*) and tetra-substituted alkenes ([CH0]=[CH0]) were incompatible.

4. Running the standardized substrate selection workflow:
   Feed the filtered list of substrates into the workflow to generate the representative set of standardized substrates. Two options are available for running the workflow:
   - Accessing the automated web-platform at https://pharmascope.uni-muenster.de/. In the "Generate/Asses Scope" tab, users can input the filtered list of potential substrates (as SMILES). The web platform allows any user to run the workflow without further knowledge about programming (see section 3.1 for details).
   - Alternatively, by running the provided Python scripts (see Section 3.2 for details).

5. Experimental reaction evaluation:
   Perform reactions with the selected substrates. Report both positive and negative results to ensure an unbiased assessment of applicability and to establish a benchmark for reaction generality.

The final substrate selection for experimental evaluation is subject to the commercial availability of the substrates. For this reason, the top-N selected substrate results should be considered.

3.1 Guidelines for using the Web-Interface

The web application (https://pharmascope.uni-muenster.de/) provides easy access to the standardized substrate selection workflow without the need for any coding or data science knowledge. The user must only prepare a CSV-, Excel-, or text file containing all SMILES[2] strings of potential substrate candidates. This may either be a large dataset of compounds (suggested when the aim is to generate a diverse, unbiased scope of molecules) or a smaller list of compounds (e.g., a handpicked collection of scope entries) that should be evaluated in terms of chemical space coverage.

Large datasets can be retrieved from common databases.[4] Smaller collections of own compounds can be transferred into SMILES strings using common software such as ChemDraw. Final CSV- and Excel files should contain all candidate SMILES in a column with the heading "SMILES", text files should only contain one SMILES string per line (Figure S2).

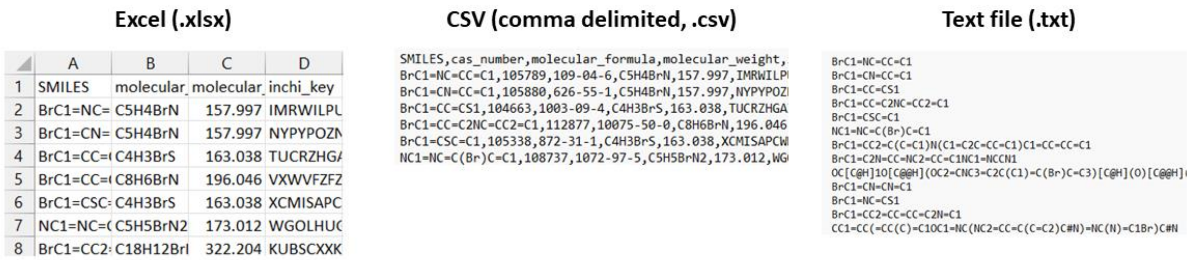

Figure S2: Compatible file types and formats to upload a collection of SMILES strings into the web application.

In the web application, the page "Generate / Assess Scope" is used to project the collection of given substrate candidates into the drug space. On this page, various parameters as well as additional filters can be set. The project name/identifier can be freely chosen and is only used to label the output files. Besides that, the user can change the following parameters if deviations from standard settings are desired (Figure S3):

- Number of clusters:
  Select how many clusters the drug space should be partitioned. The user-uploaded compounds will be assigned to those clusters.
- Top-N substrates per cluster:
  Select how many (N) compounds should be selected and exported as a potential scope entry from each cluster based on their proximity to the cluster's centre. When only assessing the drug space coverage of a small dataset, this value is meaningless and can be incremented to high values.
- Upload CSV-, Excel- or text file:
  Upload the file containing the list of all candidate molecules as SMILES strings. The detailed procedure for substrate list curation and filtering is described in Section 2.
- UMAP model:
  The UMAP model and settings to be used for dimensionality reduction of the drug space and transforming the substrate candidates. By default, the hyperparameter-optimized model with ECFP is used (refer to the main manuscript and SI section 4 for details). If further models are required, please use the Python-based workflow (refer to section 3.2).

- Manual filters:
  If not already carried out during the preparation of the upload file, some additional filters can be set up in this section. These include a molecular weight range, the removal of inorganic compounds and salts (set as on by default) as well as the removal of compounds that contain certain substructures. Substructures to filter out can be added as one SMARTS[5] string per line in the corresponding input field.

Clicking "Submit" will start the transformation of user-uploaded candidates into the drug space using the defined UMAP model. Depending on the size of the uploaded file, this process can take up to several minutes.

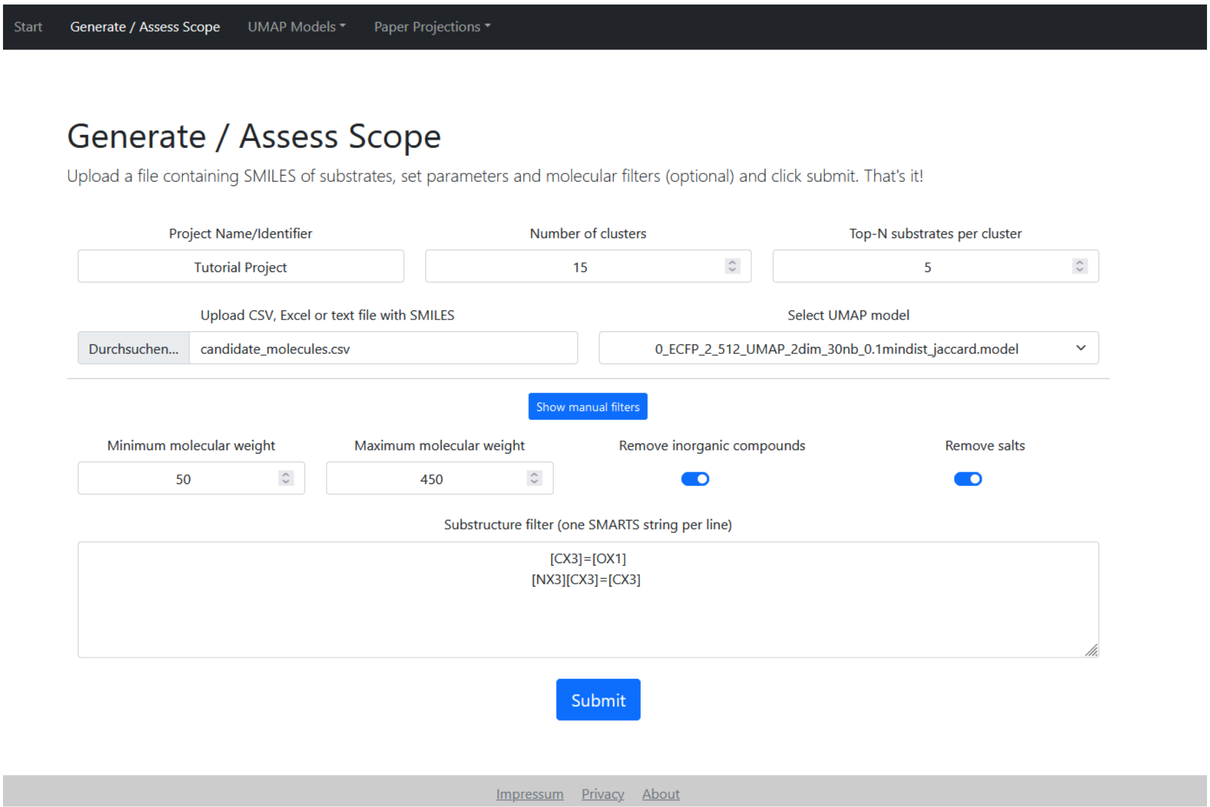

Figure S3: Starting page to generate or assess a substrate scope using the web application. Parameters and additional filters can be set here.

After the transformation is finished, an overview of the results will be displayed (Figure S4). The selected Top-N molecules per cluster will be presented along with additional information about the corresponding molecules. This table with suggestions for the standardized substrate scope can be downloaded as a CSV- or Excel file. The distribution of data points in the drug space can also be visualized (Figure S5) especially useful for qualitative assessment of drug space coverage). All results are only available as long as the session is not closed by the user. The uploaded data is only saved on the server for processing and deleted after 24 hours.

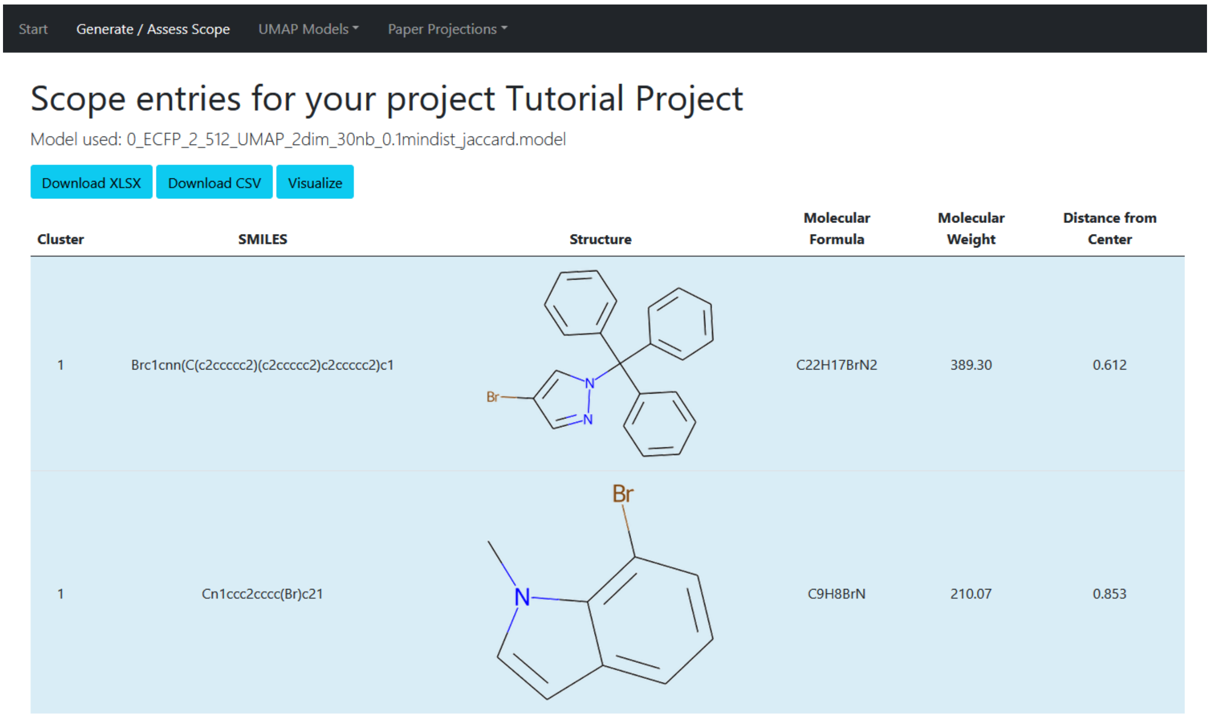

Figure S4: Results overview page of a generated unbiased scope. The selected Top-N compounds for each cluster are shown and the whole scope suggestion table can be downloaded as a CSV or Excel file.

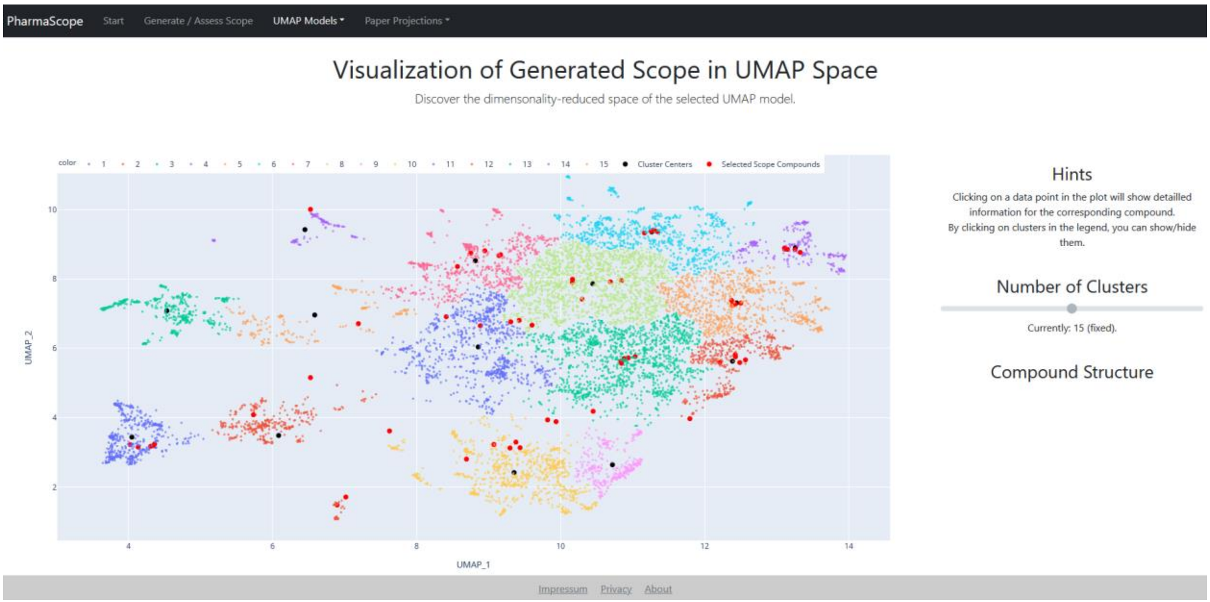

Figure S5: Visualization of the user-uploaded dataset (red circles) in drug space can be used for assessing the chemical space coverage of an existing small compound collection.

The web application is based on Python interfaces and scripts described in section 3.2 as well as HTML, CSS & Javascript within the Flask library[10] in Python.

3.2 Details of Python Scripts

standardized_scope.txt has all the dependencies specified for running the Python scripts. The workflow can be executed through the *scope_generator.py* file. The *scope_generator.py* file imports and utilizes the classes defined in the *substrate_selection.py* file to carry out all the tasks. The *substrate_selection.py* file has three classes:

Class: *Fingerprints*: generates and stores the specified molecular fingerprints and smiles for all the molecules.

Class: *Chemspace_mapper*: runs UMAP dimensionality reduction, hierarchical agglomerative clustering and selects substrate based on the specified selection strategy.

Class: *Scope_manager*: wrapper class that incorporates the functionalities of 'Fingerprints' and 'Chemspace_mapper' classes. Along with that it reads the settings provided in settings.json, loads the datasets and saves all the obtained results.

All the parameters for running the workflow can be specified using the *settings.json* file as described below.

```
{
        "dataset": ["data", "final_DB"],
        "test_dataset": ["data", "Alkenes_final"],

        "fp_settings":
        {
                "fp_type":"ECFP",
                "fp_radius":2,
                "nr_bits":512
        },

        "umap_settings":
        {
                "dimensions": 2,
                "neighbours": 30,
                "m_dist": 0.1,
                "metric": "jaccard"
        },

        "n_clusters": 15,

        "additional_settings":
        {
                "load_model": true,
                "model_path": "model_nb30_mdist0.1_ECFP4_jacc.sav",
                "exp_name": "3Aug_alk_top1",
                "topn_mol": 1,
```

```
                "selection_strategy": "centre-based"
        }
}
```

In the current setting, the project directory should be organized as follows:

```
Project_directory/
        scope_generator.py
        substrate_selection.py
        Settings/settings.json
        data/Final_DB/drug_dataset.csv
        data/Alkenes_final/final_alkenes_dataset.csv
        trained_models/model_nb30_mdist0.1_ECFP4_jacc.sav
        results/
```

“dataset”: providing the folder location for the drug dataset as a list.

“test_dataset”: providing the folder location for the respective substrate dataset as a list.

“fp_settings”: dictionary specifying the fingerprint type. Currently, ECFP and MACCS keys are included. For ECFP the desired radius and number of bits should also be specified.

“umap_settings”: dictionary specifying the UMAP parameter configuration.[11]

“n_clusters”: Number of clusters to be considered for hierarchical clustering.

“additional_settings”:

“load_model”: can be set true or false. If set as true, a trained model is used, otherwise, a new model would be trained with the given UMAP parameters.

“model_path”: if “load_model” is set as true then the model name needs to be provided. e.g., model_name.sav

“exp_name”: name of the folder under which the experiment results will be saved.

“topn_mol”: specifying the number of substrates to be selected from each cluster depending on the selection strategy.

“selection_strategy”: can be set “centre-based” or “similarity-based”.

The *correlation_metric.py file* contains the code for optimizing the UMAP parameters based on the pairwise distance correlation metric *(D)* and *optimize_silh.py* contains the code for silhouette score analysis.

4. UMAP parameters optimization

UMAP (Uniform Manifold Approximation and Projection)[11] is a dimensionality reduction algorithm that learns low-dimensional representations from a given high-dimensional input data. It first constructs a weighted graph in the high dimensional input data (ECFP4) and then learns embeddings in the lower-dimensional target space such that the spatial distance relationship between data points in the original higher-dimensional space is conserved. We chose the target embedding to be 2-dimensional for allowing comprehensible visualization. Apart from the target number of lower dimensions, the algorithm is dependent on the n_neighbors (N_b) and min_dist (M_d) parameters.[11] Another important UMAP parameter is the *metric* which controls how the distance between data points in the input higher-dimensional space is computed. Since fingerprints are binary bit vectors, we chose the *Jaccard metric (*i.e., Tanimoto distance).[12]

Based on initial analysis we found that the N_b and M_d parameters largely influence the dimensionality reduction results, so they should be chosen carefully with proper validation and interpretation.

To optimize these parameters, we employed the following two metrics:

- the average silhouette score[13] for a defined range of clusters.
- the pairwise distance correlation between the drug molecular fingerprints (original Jaccard distance) and their corresponding distance in the UMAP embedded space (D).

The average silhouette score is a widely used metric for determining the optimal number of clusters present in the data. In our context, it would indicate whether significant clustering can be achieved in the UMAP embedded drug space by measuring how well the UMAP embeddings of drug molecules in one cluster are separated from drugs in other clusters. The silhouette score varies from -1 to 1, with larger values indicating good clustering quality. While it can help in assessing the clustering quality, it cannot be solely used as an appropriate metric for choosing the parameters of UMAP. Therefore, we introduced the distance correlation metric (D). This metric gauges the amount of information retained in the reduced space in comparison to the original fingerprint space, providing direct insights for selecting UMAP parameters.

Since the underlying drug data is unlabeled there is no single perfect solution for mapping the drug molecules based on structural similarity. However, for effective mapping of the drug molecules, both local and global structural similarity information must be captured. Global features encompass widely prevalent scaffolds such as sugars, fatty acids, or β-lactams, while local features involve functional groups or substitutional variations around similar scaffolds. Therefore, we aimed to optimize the UMAP parameters considering both global and local information, based on the distance correlation metric (D) as well as the silhouette score.

In general, higher values of N_b led to an increase in the distance correlation metric. The N_b parameter controls the number of neighbouring drug molecules to be considered when constructing the graph in the higher dimensional fingerprint space. Higher values of N_b prioritize the inclusion of a larger number of neighbouring molecules, emphasizing the global

data structure. In contrast, lower values of N_b constrain the algorithm to focus more on the local structure by considering fewer neighbouring molecules (Figure S6). However, we also observed that for higher values of N_b the average silhouette score (for cluster numbers ranging from 10 to 25) slightly decreased. This suggested that data projection becomes fuzzier and loses local structural details. The M_d parameter controls the minimum distance that is allowed between two data-points in the target low-dimensional space. Higher values of the M_d parameter also resulted in lower silhouette scores indicating reduced cohesion within clusters (Figure S6).

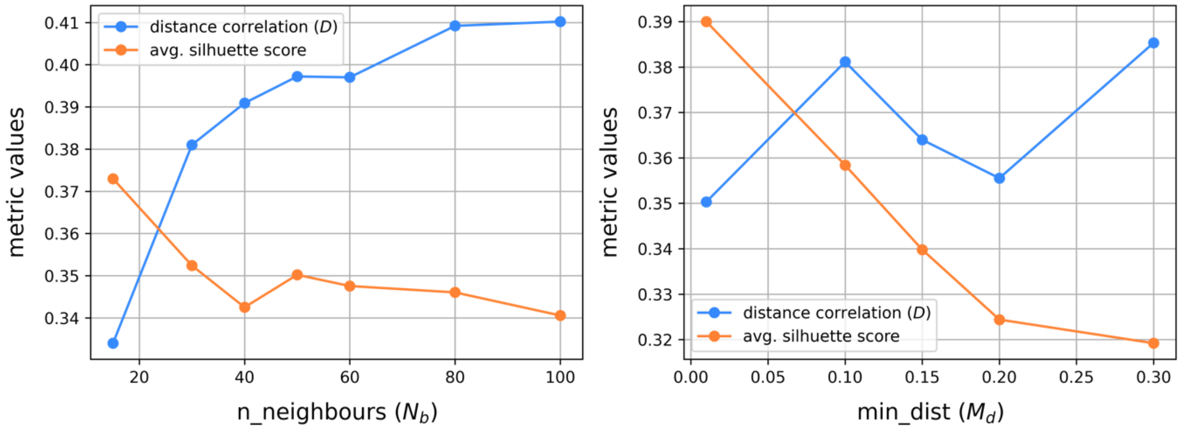

Figure S6: Relationship between pairwise distance correlation metric (blue) and average silhouette score (orange) with UMAP parameters. The plotted average silhouette scores for each parameter value are the average over cluster numbers from 10 to 25.

Overall, there can only be a tradeoff between the amount of local to global information one chooses to retain. While higher values of N_b favor the algorithm to retain more of the global similarity information among the drug molecules it pays less attention to embedding structurally similar drug molecules distinctly from others. After careful consideration to strike an optimum balance between preserving the local and global information in the data, we selected the values of 30 for N_b and 0.1 for m_dist parameters, for training the UMAP model (Figure S7).

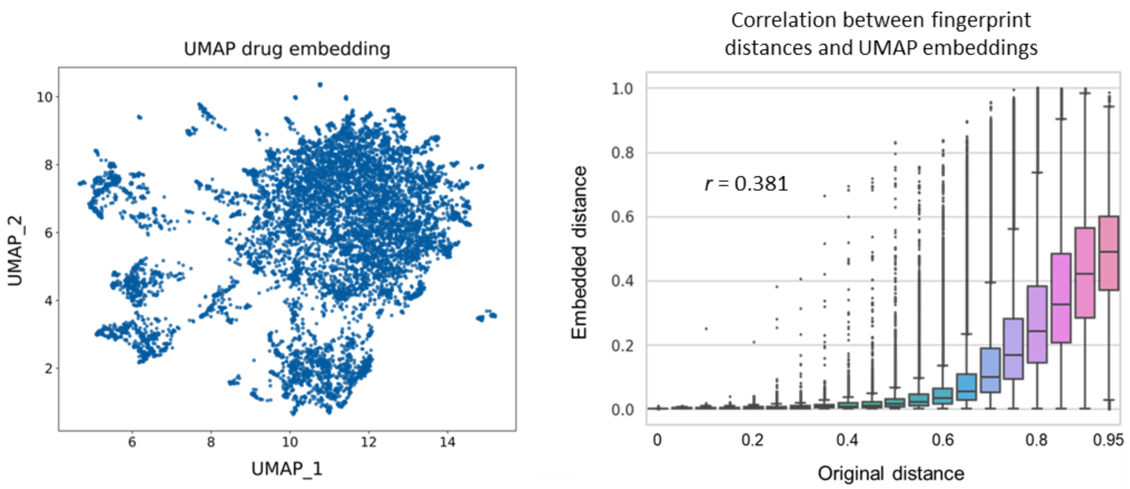

Figure S7: UMAP embeddings of drug molecules obtained with optimized model parameters: N_b=30, M_d=.0.1. Box plot representing the preservation of pairwise fingerprint similarity in the UMAP embedded space (Pearson correlation coefficient, r = 0.381).

The trained UMAP model forms the base of our substrate selection workflow. The model can be utilized for projecting any given class of substrate to obtain an overlapping drug-substrate map based on the learned drug scaffold similarity information.

5. Comparative experiments for molecular fingerprints

Molecular fingerprints are numeric representations encoding the structural information of molecules in a vector format. Various types of molecular fingerprints are known, depending upon their method of construction such as substructure-based, path-based topological fingerprints and circular fingerprints.[14] To analyze the effect of different fingerprints as molecular representations (input features) for the UMAP dimensionality reduction, MACCS keys and ECFP4 (512 bits) were considered. MACCS keys[15] are a set of 166 pre-defined substructural patterns making it a relatively simple fingerprint, whereas ECFP4[14] encodes the structural characteristics of a molecule by extracting all possible circular subgraphs up to diameter 4.

The overall average Tanimoto similarity[12] for all the drug molecules was computed to be 0.307 and 0.119 for MACCS keys and ECFP4, respectively. The comparison of the corresponding MACCS keys and ECFP4 Tanimoto similarity for each pair of drug molecules indicates that MACCS keys assign higher similarity values among the diverse drug molecules as compared to ECFP4 which differentiates the drug molecules stronger (Figure S8).

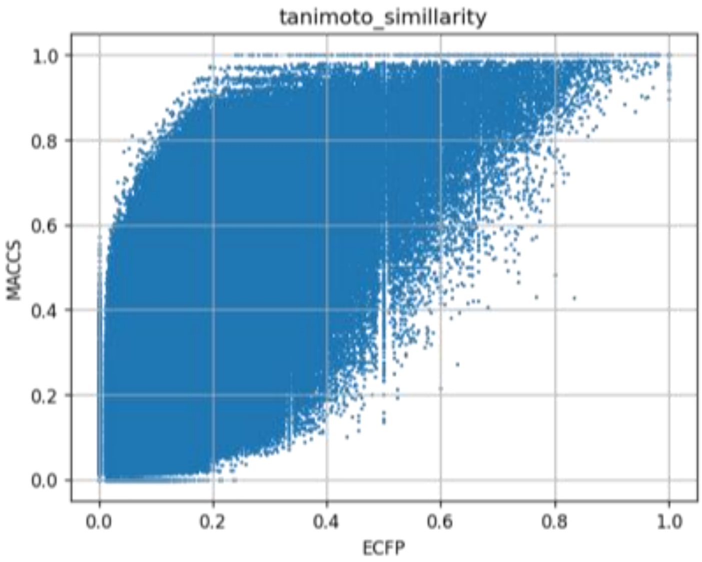

Figure S8: Tanimoto similarity scores for MACCS keys vs ECFP4 for all drug fingerprint pairs.

In order to compare MACCS keys and ECFP4 as input representation, a separate UMAP model was trained with both the fingerprints. Following parameters of 30 (*n_neighbors*), 0.1 (*min_dist*), 2 (*n_components*) and the Jaccard metric were chosen for training the UMAP algorithm. The output UMAP embeddings were subsequently clustered using hierarchical agglomerative clustering to compartmentalize the projected data. To quantify the structural similarity information captured by UMAP in mapping the drug molecules, we calculated the average cluster Tanimoto similarity by computing the fingerprint similarities of all the molecules that fall in one cluster and subsequently the average value for all the clusters was calculated. For a cluster size of 10, the average cluster Tanimoto similarity was found to be 0.456 and 0.159 for MACCS keys and ECFP4, respectively. This increase in average cluster similarity confirms that molecules lying closer in the projected space are structurally more similar.

Table S1: Analysis of average cluster Tanimoto similarity for MACCS keys and ECFP4 representation

Number of Clusters	Avg. cluster similarity ECFP	$\frac{cluster\ similarity}{overall\ similarity}$	Avg. cluster similarity MACCS	$\frac{cluster\ similarity}{overall\ similarity}$
10	0.159	1.341	0.456	1.481
15	0.176	1.482	0.463	1.504
20	0.183	1.535	0.475	1.542
25	0.187	1.573	0.481	1.562
50	0.2	1.677	0.512	1.664
100	0.23	1.929	0.552	1.794

To further investigate the similarity of molecules located in the proximate neighbourhood, the average cluster similarity was computed for a higher number of clusters, resulting in a finer partitioning of the space. While there is a steady increase in the average cluster similarity as the total number of clusters increases, ECFP4 has significantly higher ratios of cluster-to-overall similarity at larger numbers of clusters (Table S1). This suggests that ECFP4 is a more unique and distinct fingerprint representation in terms of molecular similarity.

While both fingerprints could be used as molecular representations for the UMAP dimensionality reduction, ECFP4 offers a more flexible and robust encoding of the structural information as compared to the predefined sets of MACCS keys. Therefore, we selected ECFP4 as the molecular representation for the further development of our workflow.

6. Clustering UMAP embeddings

For diversely sampling substrates from the drug-substrate map with maximum coverage, we sought to identify clusters in the drug map. The aim was to partition the embedded drug chemical space into different regions such that drug molecules sharing structural similarity fall in the same cluster. These regions could then be used as reference points for sampling the substrates when they are projected to generate the drug-substrate map. Accordingly, two clustering algorithms: k-means and hierarchical agglomerative clustering were evaluated. It should be mentioned again for clarity that the clustering was performed only on the drug embeddings, without the consideration of any substrates.

The K-means clustering algorithm iteratively assigns data points to k clusters based on the distance to cluster centroids, aiming to minimize the within-cluster sum of squares. However, its performance is sensitive to the initial centroid placement, which can lead to local optima. Additionally, due to the centroid-based distance minimization, it assumes the clusters to be of similar shapes and tends to form spherical clusters, making it less effective for datasets with varying cluster shapes and densities, such as in the given UMAP drug embedding. On the other hand, the hierarchical agglomerative clustering algorithm[16] offers better adaptability for datasets with varying cluster shapes and densities. Initially, the algorithm considers each molecule (data point) as an individual cluster and then iteratively merges them to build up larger clusters making it a “bottom-up” approach. This iterative merging process is based on the Euclidean distance metric and continues until a single cluster or until the desired number of clusters is achieved. Consequently, we opted for hierarchical agglomerative clustering with Ward linkage and maxclust criteria.

For determining the optimal number of clusters in the embedded space, we conducted the silhouette score analysis. Since the number of clusters determined the size of the substrate scope, we considered the cluster numbers ranging from 10 to 25. The average silhouette scores did not reveal any significant trend (Figure S10). Further investigation revealed that the clustering algorithm identifies a number of separated clusters alongside a large cluttered region (Figure S9). We observed that for higher number of clusters, the clustering algorithm starts to partition the cluttered region into finer sections, leading to minimal change in the silhouette scores (Figure S9). Finally, we chose 15 clusters for practical reasons. However, it should be noted that for a finer sampling of the space, a greater number of clusters can also be selected. While the decision for more clusters could give more detailed information about the potential scope and limitations of any reaction, it also reduces comparability.

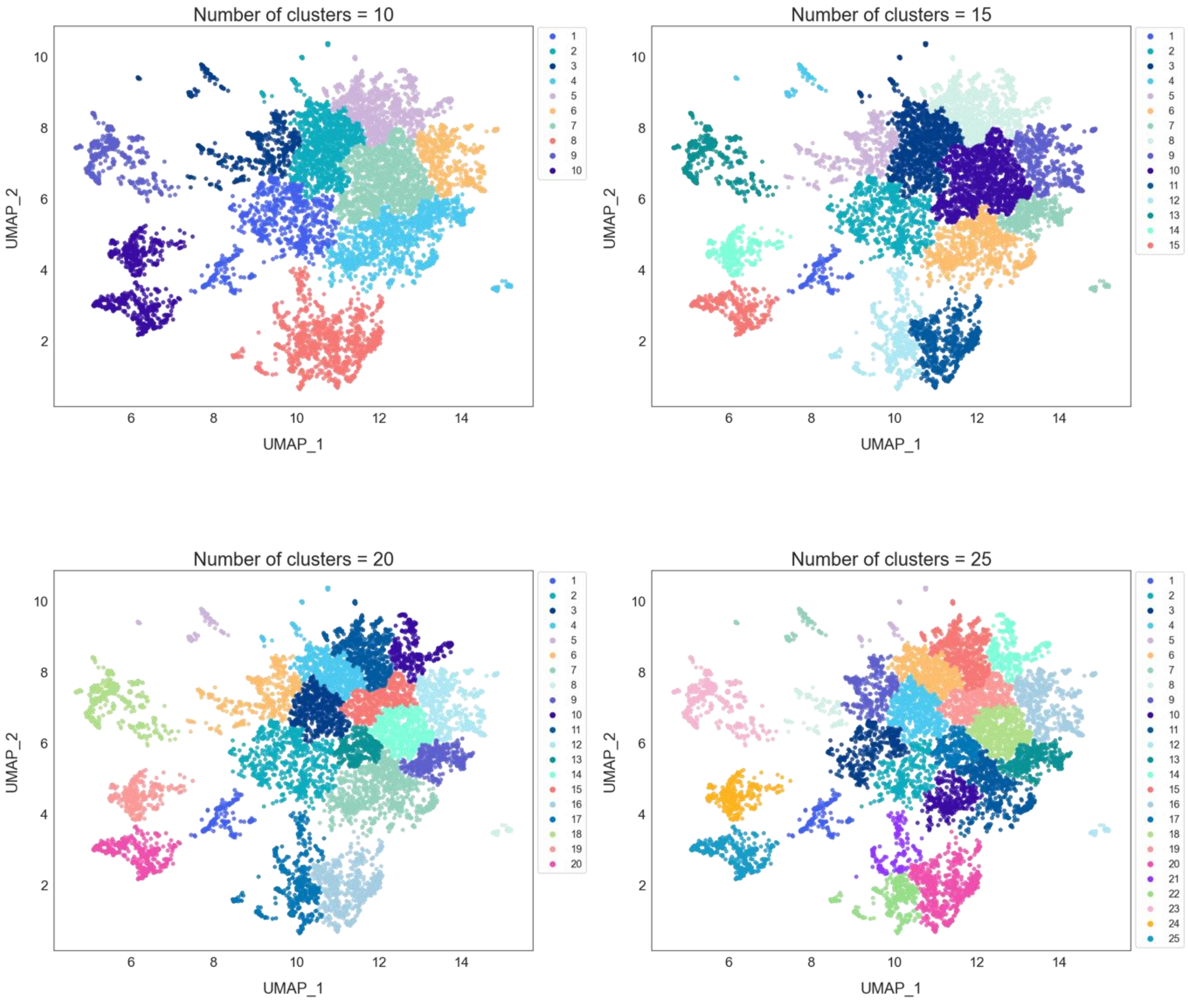

Figure S9: Comparison of hierarchical clustering of the drug embeddings with varying number of cluster.

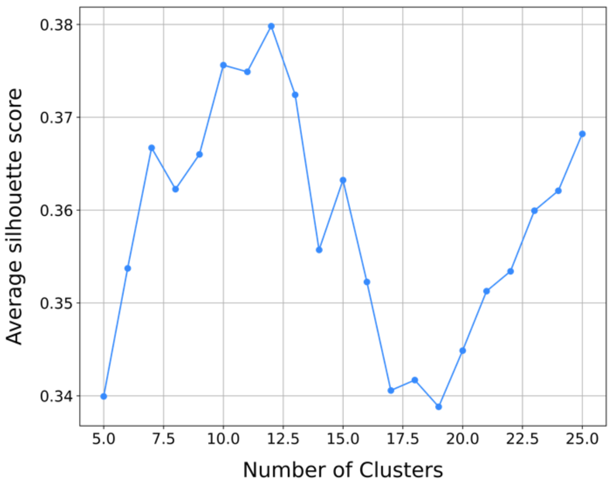

Figure S10: Relationship between the number of clusters and the average silhouette score for the drug embeddings.

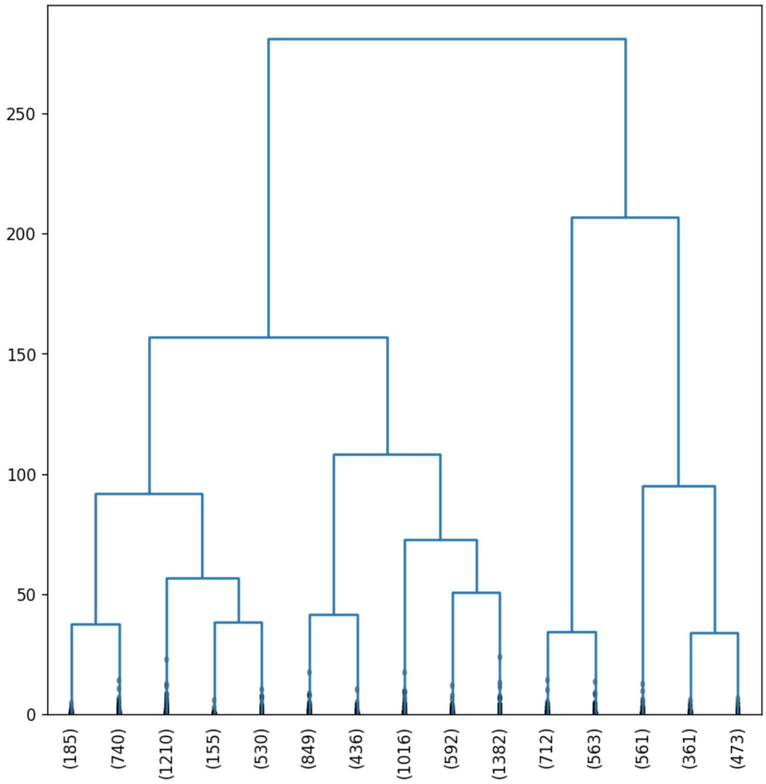

Figure S11: Dendrogram representing the hierarchical relationship of the 15 drug clusters.

7. Different substrate selection strategies

The following two strategies were evaluated for substrate selection from the drug-substrate map:

- Selecting substrates lying closest to the center of each drug cluster.
- Selecting substrates with the highest average Tanimoto similarity to drugs in the respective clusters.

The top-5 selections for each strategy can be found below. Both strategies yielded a structurally diverse set of alkenes and could be used. However, we chose the centermost alkene selection strategy since Tanimoto similarity is biased towards selecting larger molecules due to higher average similarity scores. The final substrates were then selected for experimental evaluation from the top-*N*, depending on their commercial availability (Figure S13).

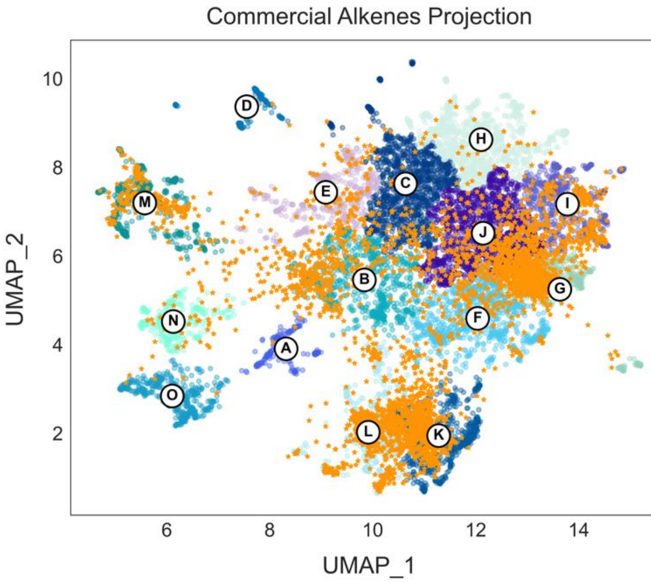

Figure S12: Commercial alkenes (yellow) projected over the drug chemical space using the UMAP model trained on drugs. Alphabet labels represent the centre of drug clusters.

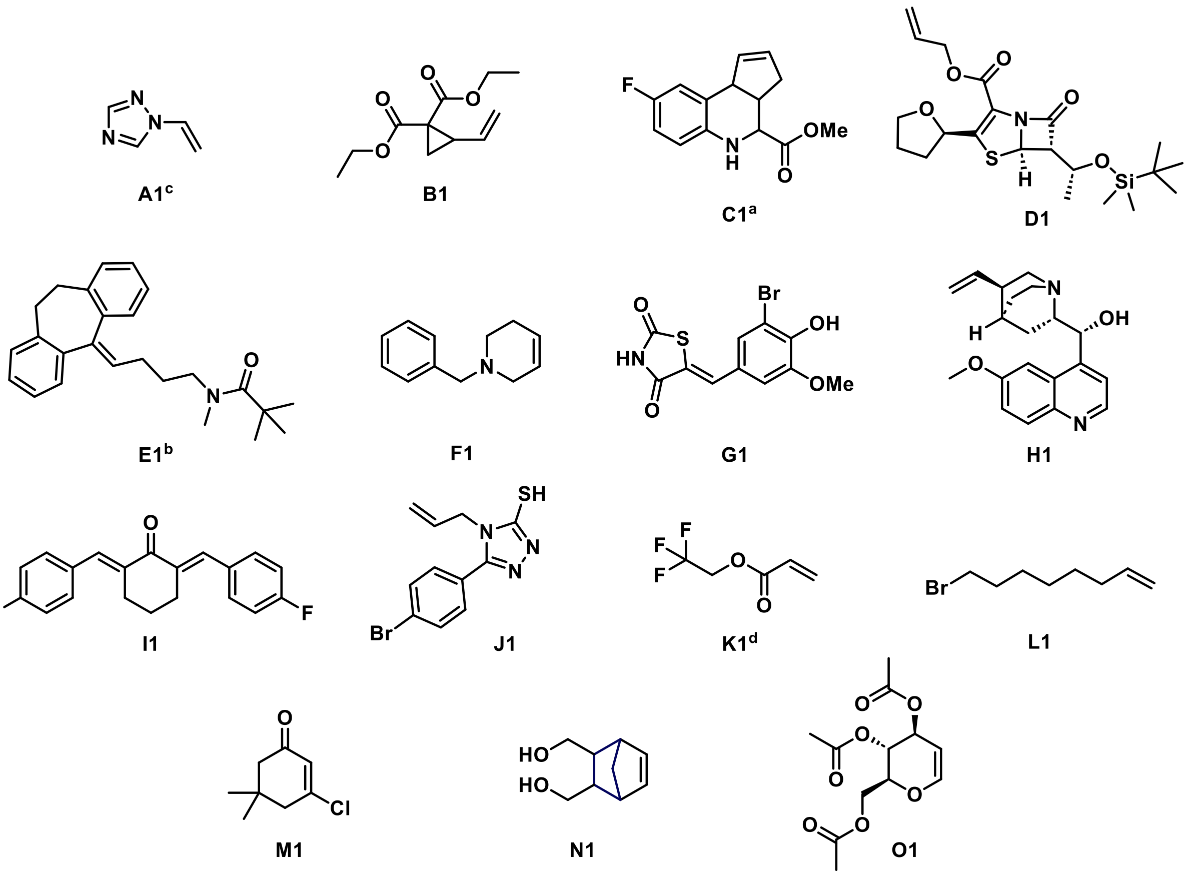

Figure S13: Alkenes selected for experimental evaluation. These alkenes were chosen based on the centremost substrate selection strategy and commercial availability. [a] the corresponding acid was converted to methyl ester. [b] the corresponding amine was converted to amide. [c] the substrate is the 6th closest to the centre. [d] the substrate is the 8th closest to the centre.

Top 5 alkenes lying closest to the centre of each drug cluster:

Cluster A:

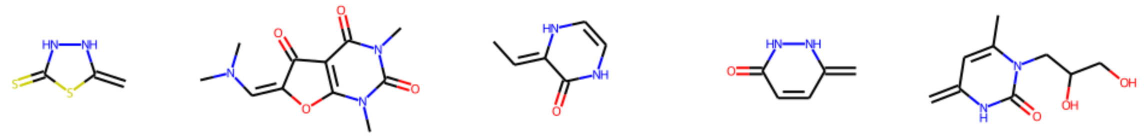

Cluster B:

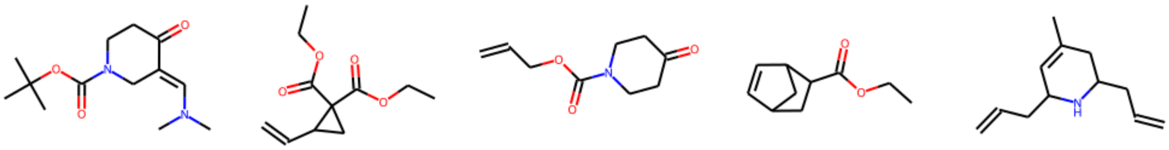

Cluster C:

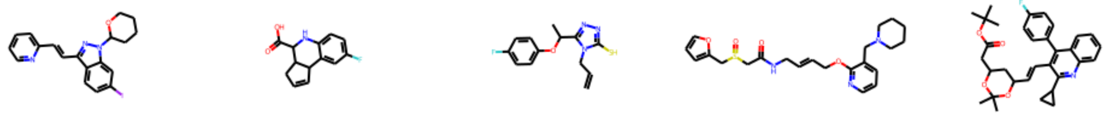

Cluster D:

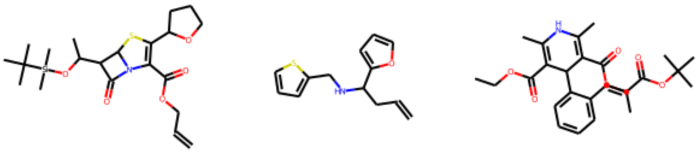

Cluster E:

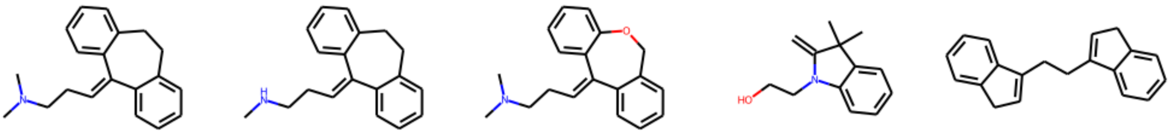

Cluster F:

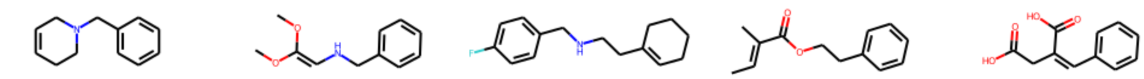

Cluster G:

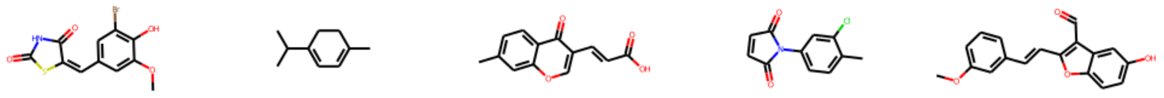

Cluster H:

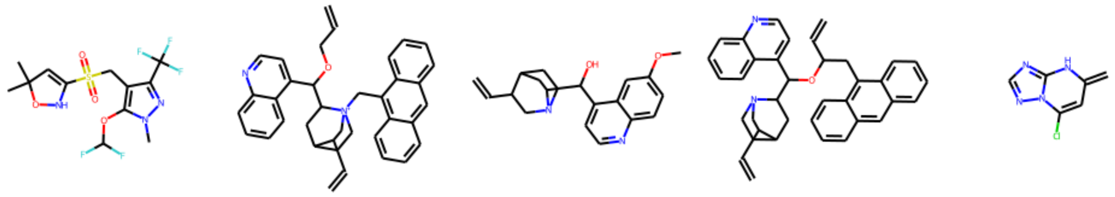

Cluster I:

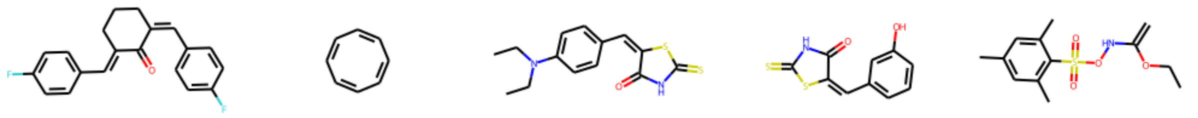

Cluster J:

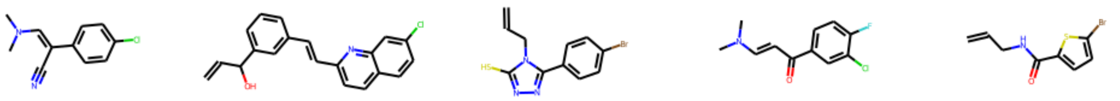

Cluster K:

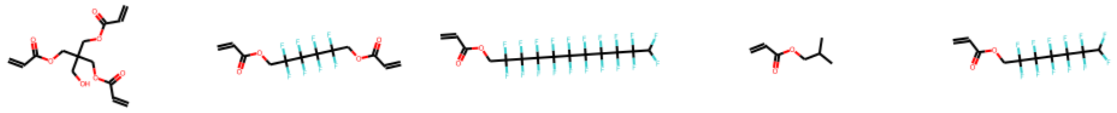

Cluster L:

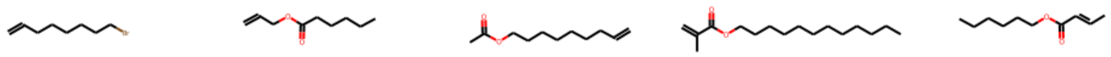

Cluster M:

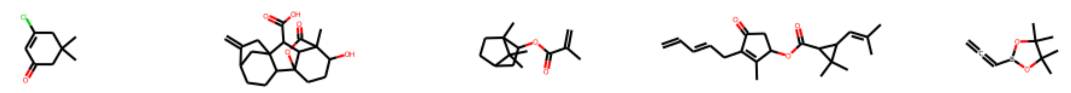

Cluster N:

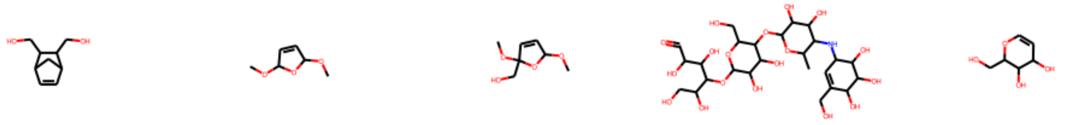

Cluster O:

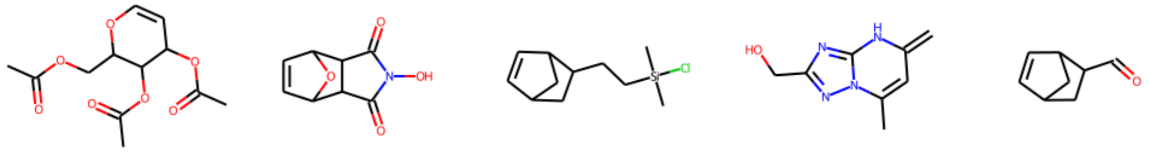

Top 5 alkenes based on highest average Tanimoto similarity to drugs:

Cluster A:

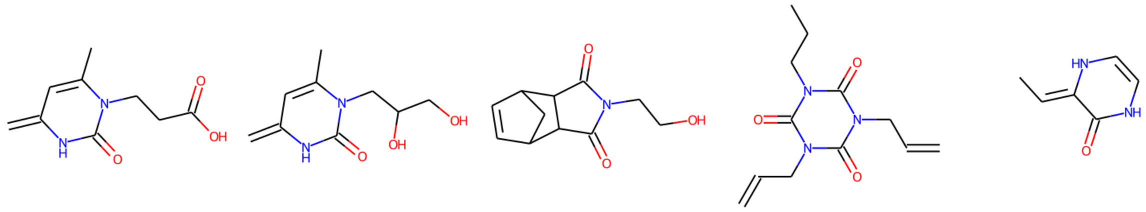

Cluster B:

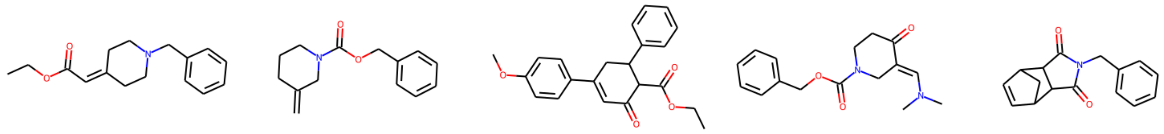

Cluster C:

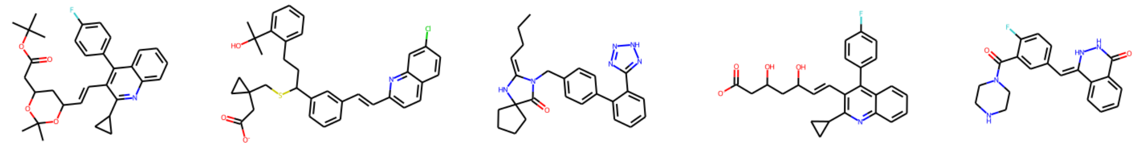

Cluster D:

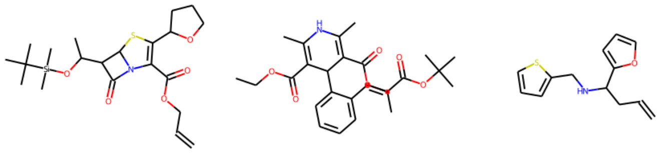

Cluster E:

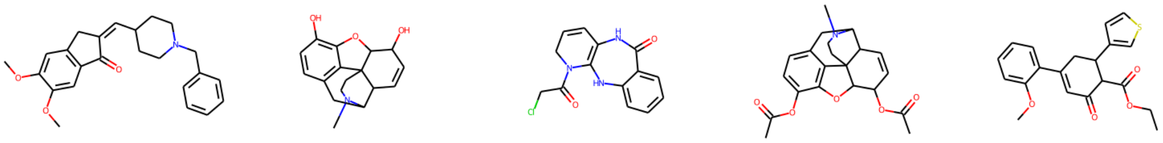

Cluster F:

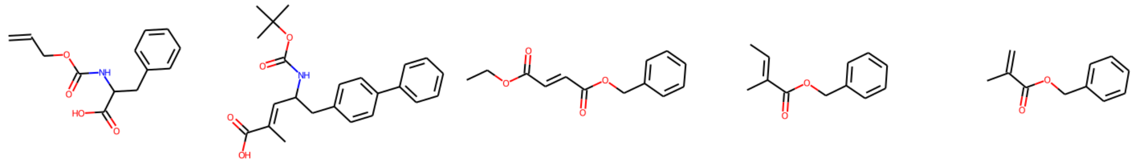

Cluster G:

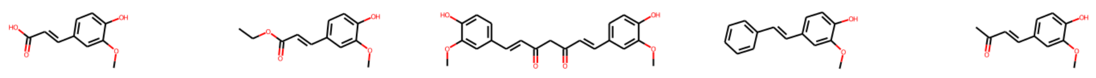

Cluster H:

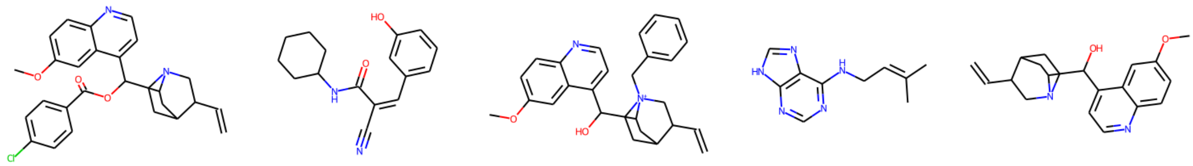

Cluster I:

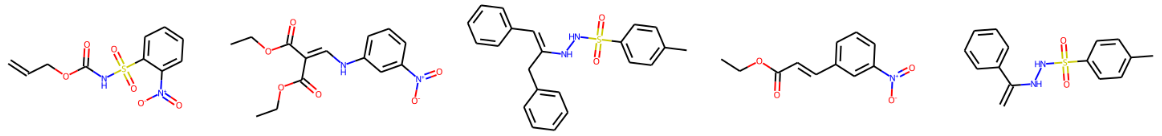

Cluster J:

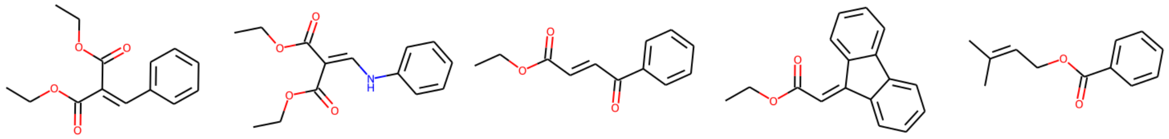

Cluster K:

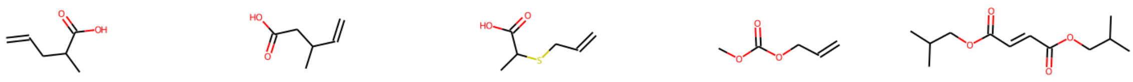

Cluster L:

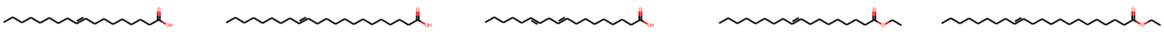

Cluster M:

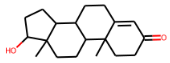 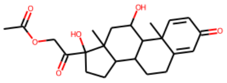 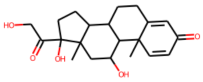 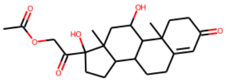 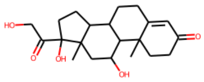

Cluster N:

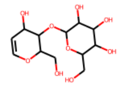 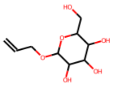 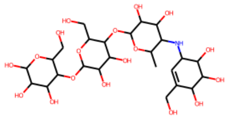 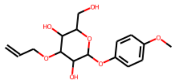 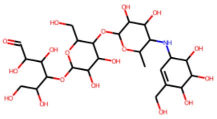

Cluster O:

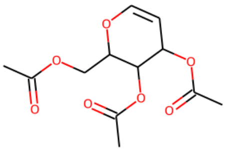 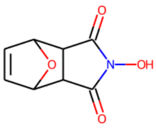 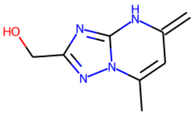 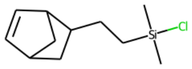 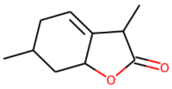

8. Application to other substrate classes

The transferability of the standardized substrate selection workflow was tested for other substrate classes: aryl bromides and heteroaryl bromides. The details of the dataset curation and preprocessing are discussed in detail in Section 2. The filtered datasets were projected utilizing the trained UMAP model to generate the drug-substrate maps (Figure S14, S16). Subsequently, the aryl/heteroaryl bromides lying closest to the center of each drug cluster were chosen for the standardized set of substrates (Figure S15, S17). It can be observed that the distribution of the projected aryl bromides (yellow) over the drug embeddings is non-uniform. This arises due to the fact that aryl bromides do not share the same scaffold complexity as compared to various drugs. For example, cluster D has no aryl bromide in its proximity. In such scenarios, one possible solution could be to partition the drug space into more clusters such that better sampling from the overlapped drug-substrate regions can be made.

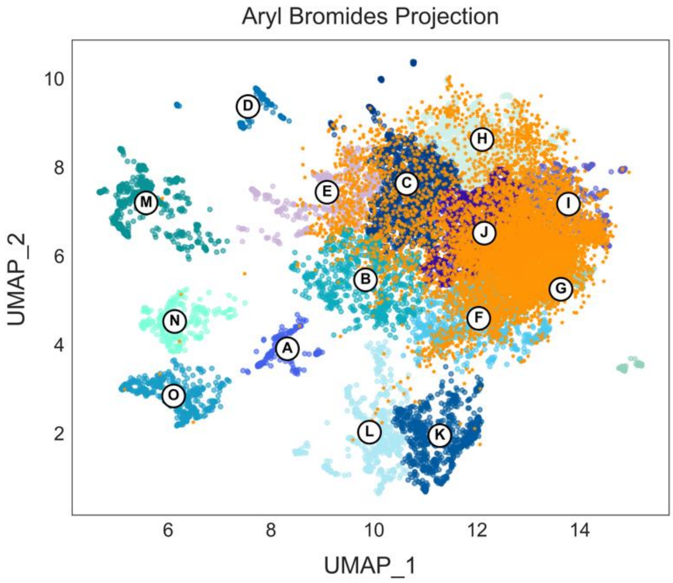

Figure S14: Projection of aryl bromides (yellow) over the drug chemical space with the trained UMAP model. Alphabet labels indicating centre of drug clusters.

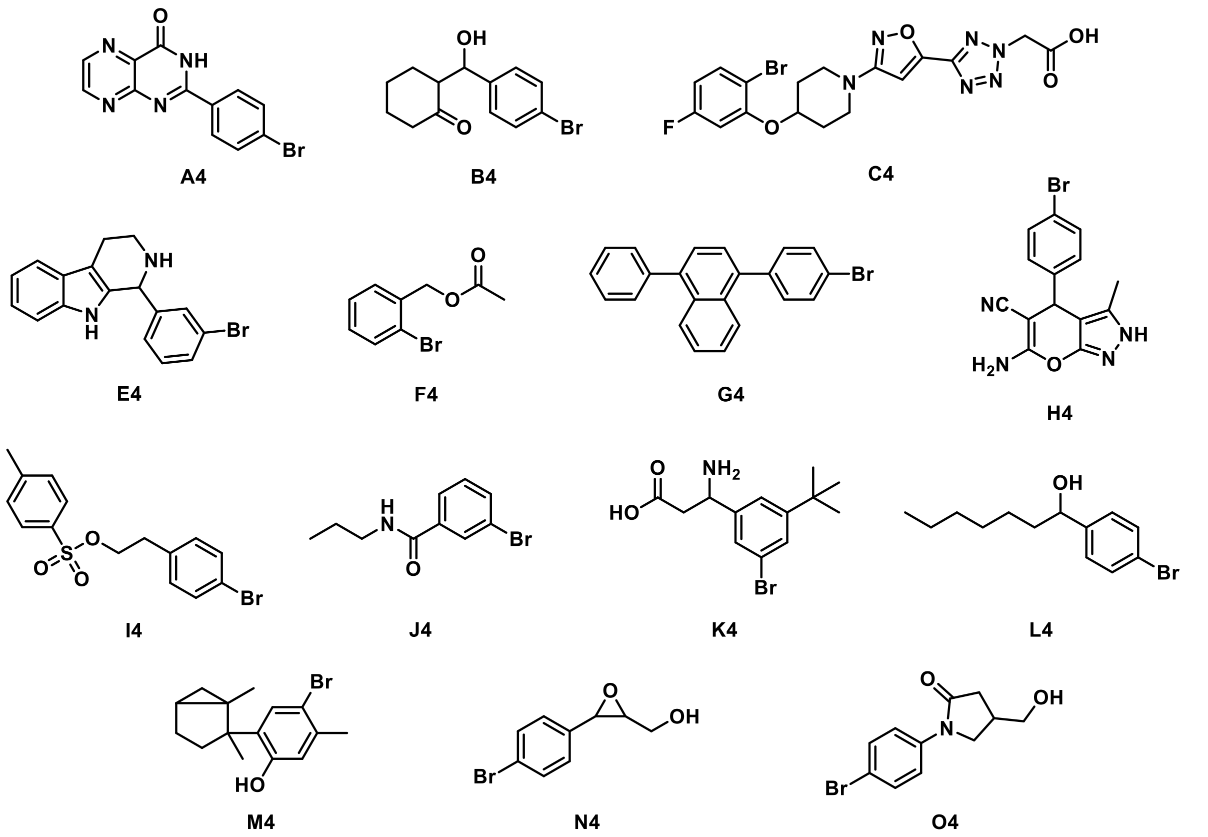

Figure S15: Representative set of aryl bromides selected by the standardized substrate selection workflow. Cluster D has no aryl bromides in its proximity.

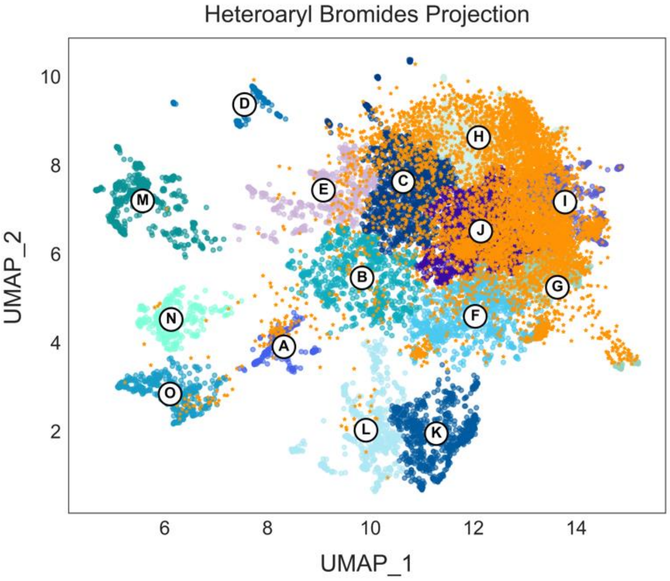

Figure S16: Projection of heteroaryl bromides (yellow) over the drug chemical space with the trained UMAP model. Alphabet labels indicating centre of drug clusters.

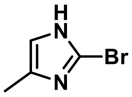

A5

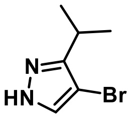

B5

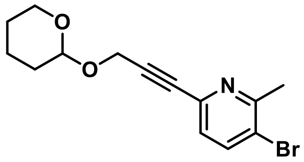

C5

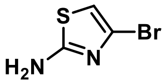

D5

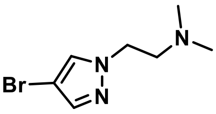

E4

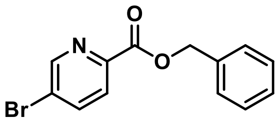

F4

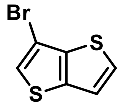

G4

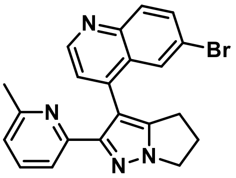

H5

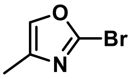

I5

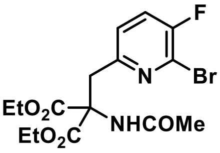

J5

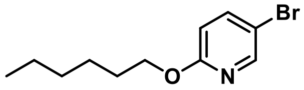

L5

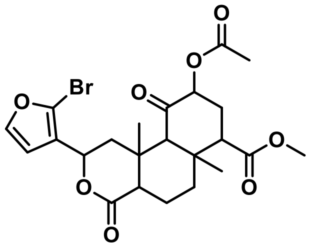

M5

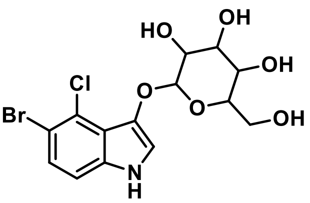

N5

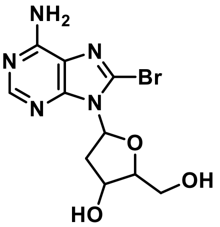

O5

Figure S17: Representative set of heteroaryl bromides selected by the standardized substrate selection workflow. Cluster K has no heteroaryl bromide in its proximity.

9. Experimental details

9.1 General procedure for photocatalytic imino-carboxylation

General procedure A (GP-A):

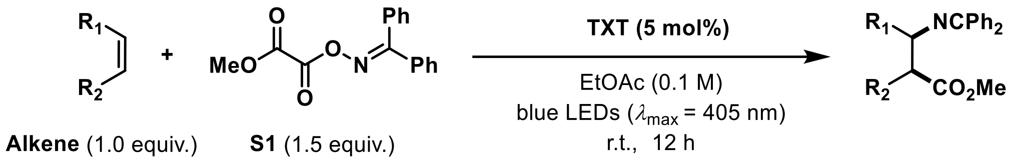

The synthesis of the bifunctional oxime ester **S1** and the photocatalytic reaction was performed following the reported literature procedure.[3] In an oven dried 10 ml Schlenk tube equipped with a teflon coated stirring bar, the oxime ester **S1** (85 mg, 0.3 mmol, 1.5 equiv.), alkene (0.2 mmol, 1.0 equiv., if solid) and thioxanthone (2.1 mg, 5 mol%) were charged under air, then the Schlenk tube was evacuated and refilled with argon for three times. Following this, dry EtOAc (0.1 M) and the appropriate alkene (0.2 mmol, 1.0 equiv., if liquid) were added under counter argon flow. The Schlenk tube was tightly sealed and stirred under irradiation with 18 W blue LEDs (λ_{max} = 405 nm) at room temperature for 12 hours. Upon completion, the resulting homogeneous solution was transferred to a 25 mL round bottom flask with the aid of DCM (3 x 5 mL). NEt_3 (approx. 0.5 mL) and SiO_2 were added to the solution and volatiles were removed under reduced pressure, affording a powder which was loaded on the column. Purification by flash column chromatography on SiO_2, pre-basified with NEt_3 using pentane: EtOAc mixtures afforded the corresponding beta-amino acid derivative products.

9.2 General procedure for osmium-catalyzed dihydroxylation

General procedure B (GP-B):

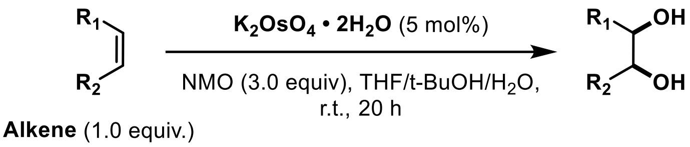

In a round bottom flask equipped with a stir bar and a glass stopper, the respective alkene (0.2 mmol, 1.0 equiv., if solid), $K_2OsO_4{\cdot}2H_2O$ (3.7 mg, 0.01 mmol, 5.0 mol%), 4-methylmorpholine 4-oxide (NMO, 70.3 mg, 0.6 mmol, 3.0 equiv.) were added.[17,18] Following this, *t*-BuOH: THF: H_2O (3.0 mL, 1:1:1 mixture) and the alkene (0.2 mmol, 1.0 equiv., if liquid) were added. The resulting mixture was stirred for 20 hours at room temperature. Upon completion, the reaction was quenched with Na_2SO_3 (252 mg, 2.0 mmol) and stirred for 1 hour, followed by extraction with CH_2Cl_2 or EtOAc (3 x 15 mL). The combined organic layers were washed with water, brine, dried over $MgSO_4$, filtered and the solvent was removed under reduced pressure. Purification by column chromatography on silica gel using pentane: EtOAc or CH_2Cl_2: MeOH mixtures afforded the corresponding diol products.

General procedure C (GP-C):

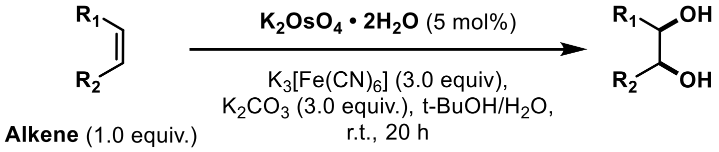

In a round bottom flask equipped with a stir bar and a glass stopper, the alkene (0.2 mmol, 1.0 equiv., if solid), $K_2OsO_4 \cdot 2H_2O$ (3.6 mg, 0.01 mmol, 5.0 mol%), $K_3[Fe(CN)_6]$ (198 mg, 0.6 mmol, 3.0 equiv.), K_2CO_3 (83 mg, 0.6 mmol, 3.0 equiv.) were added.[19,20] For tri-substituted olefins, $MeSO_2NH_2$ (19 mg, 0.2 mmol, 1.0 equiv.) was added.[19] Following this, *t*-BuOH: H_2O (3 mL, 1:1 mixture) and the alkene (0.2 mmol, 1.0 equiv., if liquid) were added. The resulting mixture was stirred for 20 hours at room temperature. Upon completion, the reaction was quenched with Na_2SO_3 (252 mg, 2.0 mmol) and stirred for 1 hour, followed by extraction with CH_2Cl_2 or EtOAc (3 x 15 mL). The combined organic layers were washed with water, brine, KOH (1.0 M, 5 mL, if $MeSO_2NH_2$ was used), dried over $MgSO_4$, filtered and then the solvent was removed under reduced pressure. Purification by column chromatography on silica gel using pentane: EtOAc or CH_2Cl_2: MeOH mixtures afforded the corresponding diol products.

10. Experimental data for the synthesized products

Methyl 8-fluoro-1,2-dihydroxy-2,3,3a,4,5,9b-hexahydro-1H-cyclopenta[c]quinoline-4-carboxylate (C2)

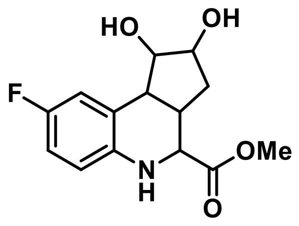

C2

Following *GP-C*, the title compound was prepared from methyl 8-fluoro-3a,4,5,9b-tetrahydro-3H-cyclopenta[c]quinoline-4-carboxylate (49.5 mg, 0.2 mmol, 1.0 equiv.). Purification by column chromatography on silica gel (CH_2Cl_2/MeOH: 100/1 to 30/1) afforded the title compound as a white solid in 50:50 diastereomeric ratio (34.88 mg, 0.124 mmol, 62%). Combined NMR data for both the isomers are reported below.

$\boldsymbol{R_f}$ = 0.35 (CH_2Cl_2/MeOH = 25/1).

^{1}H NMR (400 MHz, MeOD) δ 6.94 – 6.83 (m, 1H), 6.78 – 6.69 (m, 2H), 3.96 – 3.88 (m, 1H), 3.82 – 3.74 (m, 4H), 3.67 – 3.62 (m, 1H), 3.30 – 3.19 (m, 2H), 1.85 – 1.73 (m, 1H), 1.73 – 1.62 (m, 1H).

^{13}C NMR (101 MHz, MeOD) δ 173.63, 159.29, 156.95, 142.97, 142.95, 128.58, 128.51, 117.59, 117.52, 115.69, 115.46, 114.25, 114.02, 83.33, 73.90, 59.19, 52.49, 46.63, 46.62, 39.59, 31.80.

HRMS (ESI): calculated for $C_{14}H_{16}NO_4FNa^+$ ($[M+Na]^+$): 304.09556, found 304.09559.

2,3-dihydroxypropyl (5R,6S)-6-((R)-1-((tert-butyldimethylsilyl)oxy)ethyl)-7-oxo-3-((R)-tetrahydrofuran-2-yl)-4-thia-1-azabicyclo[3.2.0]hept-2-ene-2-carboxylate (D2)

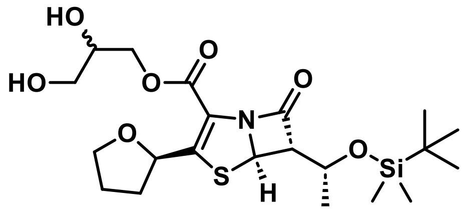

D2

Following *GP-B*, the title compound was prepared from allyl (5R,6S)-6-((R)-1-((tert-butyldimethylsilyl)oxy)ethyl)-7-oxo-3-((R)-tetrahydrofuran-2-yl)-4-thia-1 azabicyclo[3.2.0]hept-2-ene-2-carboxylate (88 mg, 0.2 mmol, 1.0 equiv.). Purification by column chromatography on silica gel (n-pentane/EtOAc: 10/1 to 1/1) afforded the title compound as a colourless oil in 55:45 diastereomeric ratio (68.2 mg, 0.144 mmol, 72%). Combined NMR data for both isomers are reported below.

***R*_f** = 0.32 (CH_2Cl_2/MeOH = 25/1).

^{1}H NMR (400 MHz, $CDCl_3$) δ 5.57 – 5.50 (m, 1H), 5.42 – 5.34 (m, 1H), 4.69 – 4.40 (m, 1H), 4.29 – 4.17 (m, 1H), 4.06 – 3.81 (m, 5H), 3.75 – 3.72 (m, 1H), 3.7 – 3.56 (m, 2H), 2.54 – 2.4 (m, 1H), 2.36 – 2.12 (m, 1H), 2.07 – 1.90 (m, 2H), 1.87 – 1.74 (m, 1H), 1.3 – 1.16 (m, 3H), 0.88 (s, 9H), 0.07 (s, 6H).

^{13}C NMR (101 MHz, $CDCl_3$) δ 173.95, 173.93, 163.09, 163.07, 159.00, 158.85, 117.68, 117.66, 74.95, 74.91, 71.63, 71.42, 69.82, 69.72, 69.35, 66.45, 65.73, 64.82, 64.72, 63.18, 62.80, 61.48, 61.44, 33.54, 26.12, 25.67, 25.65, 22.28, 22.23, 17.97, 17.95, -4.29, -4.32, -5.15, -5.24, -5.29.

HRMS (ESI): calculated for $C_{21}H_{35}NO_7SSiNa^+$ ($[M+Na]^+$): 496.17957, found 496.17855.

N-(4-hydroxy-4-(5-hydroxy-10,11-dihydro-5H-dibenzo[a,d][7]annulen-5-yl)butyl)-N-methylpivalamide (E2)

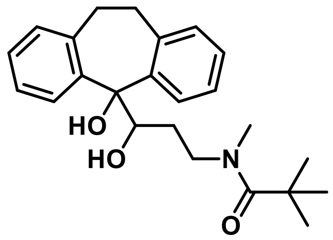

E2

Following *GP-C*, the title compound was prepared from N-(3-(10,11-dihydro-5H-dibenzo[a,d][7]annulen-5-ylidene)propyl)-N-methylpivalamide (69.5 mg, 0.2 mmol, 1.0 equiv.). Purification by column chromatography on silica gel (n-pentane/EtOAc: 20/1 to 3/1) afforded the title compound (33.31 mg, 0.148 mmol, 74%) as a yellowish oil.

***R*_f** = 0.25 (n-pentane/EtOAc = 5/1).

^{1}H NMR (400 MHz, $CDCl_3$) δ 7.95 (dd, *J* = 7.7, 1.7 Hz, 1H), 7.81 (dd, *J* = 7.8, 1.7 Hz, 1H), 7.23 – 7.11 (m, 4H), 7.1 – 7.03 (m, 2H), 4.58 (dd, *J* = 11.2, 2.3 Hz, 1H), 4.3 – 3.95 (m, 2H), 3.50 – 3.38 (m, 1H), 3.36 – 3.25 (m, 1H), 3.04 – 3.2 (m, 2H), 3.00 (s, 3H), 2.95 – 2.78 (m, 2H), 1.64 – 1.52 (m, 1H), 1.47 – 1.37 (m, 1H), 1.26 (s, 9H).

^{13}C NMR (101 MHz, $CDCl_3$) δ 178.91, 143.76, 142.53, 136.37, 135.98, 131.33, 129.96, 127.39, 127.21, 126.42, 126.21, 126.16, 125.78, 78.72, 71.64, 46.60, 38.88, 36.51, 32.93, 28.75, 28.12.

HRMS (ESI): calculated for $C_{24}H_{31}NO_3Na^+$ ($[M+Na]^+$): 404.21962, found 404.21916.

1-benzylpiperidine-3,4-diol (F2)

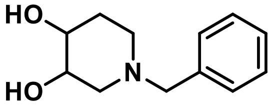

F2

Following *GP-B*, the title compound was prepared from 1-benzyl-1,2,3,6-tetrahydropyridine (34.65 mg, 0.2 mmol, 1.0 equiv.). Purification by column chromatography on silica gel (CH_2Cl_2/MeOH: 50/1 to 12/1) afforded the title compound in >95:5 diastereomeric ratio (cis:trans) as a colourless oil (24 mg, 0.116 mmol, 58%). Combined NMR data for both isomers are reported below.

R$_f$ = 0.4 (CH_2Cl_2/MeOH = 10/1).

^{1}H NMR (400 MHz, $CDCl_3$) δ 7.38 – 7.23 (m, 5H), 3.85 – 3.75 (m, 1H), 3.63 – 3.54 (m, 3H), 2.99 – 2.81 (m, 1H), 2.80 – 2.46 (m, 3H), 2.33 (d, *J* = 11.8 Hz, 1H), 2.23 – 2.06 (m, 1H), 1.89 – 1.67 (m, 2H).
^{13}C NMR (101 MHz, $CDCl_3$) δ 137.27, 129.15, 128.44, 127.50, 69.21, 68.58, 62.13, 56.70, 50.32, 29.68.

HRMS (ESI): calculated for $C_{12}H_{17}NO_2Na^+$ ($[M+Na]^+$): 230.11515, found 230.11512.

1-((3R,4S,6S)-6-((R)-hydroxy(6-methoxyquinolin-4-yl)methyl)quinuclidin-3-yl)ethane-1,2-diol (H2)

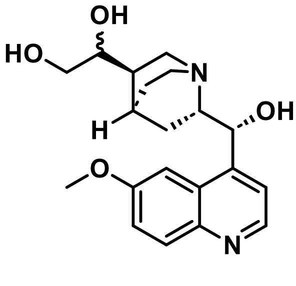

H2

Following *GP-C*, the title compound was prepared from (R)-[(2S,4S,5R)-5-ethenyl-1-azabicyclo[2.2.2]octan-2-yl]-(6-methoxyquinolin-4-yl)methanol (64.9 mg, 0.2 mmol, 1.0 equiv.). Purification by column chromatography on silica gel (CH_2Cl_2/MeOH: 20/1 to 5/1) afforded the title compound in 71:29 diastereomeric ratio as a white solid (30.1 mg, 0.84 mmol, 42%). Combined NMR data for both the isomers are reported below.

R$_f$ = 0.15 (CH_2Cl_2/MeOH = 5/1).

^{1}H NMR (400 MHz, MeOD) δ 8.69 – 8.61 (m, 1H), 7.97 – 7.88 (m, 1H), 7.74 – 7.62 (m, 1H), 7.43 – 7.4 (m, 1H), 7.39 – 7.34 (m, 1H), 5.68 – 5.6 (m, 1H), 4.04 – 3.87 (m, 4H), 3.80 – 3.57 (m, 2H), 3.51 – 3.37 (m, 1H), 3.1 – 2.98 (m, 1H), 2.97 – 2.72 (m, 3H), 2.28 – 2.11 (m, 1H), 1.81 – 1.58 (m, 2H), 1.57 – 1.46 (m, 2H), 1.16 – 0.99 (m, 1H).

^{13}C NMR (101 MHz, MeOD) δ 159.64, 159.61, 150.81, 150.74, 148.14, 148.12, 144.74, 144.69, 131.33, 131.30, 128.18, 128.03, 123.38, 119.93, 119.84, 102.38, 102.28, 74.94, 73.31, 72.44, 72.41, 66.18, 65.86, 60.72, 60.54, 56.41, 51.06, 50.97, 39.17, 39.05, 27.80, 27.42, 25.66, 23.64, 22.07, 20.95.

HRMS (ESI): calculated for $C_{20}H_{26}N_2O_4Na^+$ ($[M+Na]^+$): 381.17848, found 381.17852.

8-bromooctane-1,2-diol (L2)

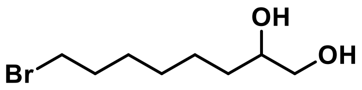

L2

Following *GP-B*, the title compound was prepared from 8-bromooct-1-ene (38.22 mg, 0.2 mmol, 1.0 equiv.). Purification by column chromatography on silica gel (CH_2Cl_2/MeOH: 100/1 to 20/1) afforded the title compound (33.31 mg, 0.148 mmol, 74%) as a yellowish oil.

R_f = 0.32 (CH_2Cl_2/MeOH = 25/1).

^{1}H NMR (400 MHz, $CDCl_3$) δ 3.75 – 3.60 (m, 2H), 3.46 – 3.34 (m, 3H), 2.32 (s, 2H), 1.92 – 1.80 (m, 2H), 1.53 – 1.28 (m, 8H).

^{13}C NMR (101 MHz, $CDCl_3$) δ 72.23, 66.83, 33.95, 33.01, 32.68, 28.76, 28.05, 25.39.

HRMS (ESI): calculated for $C_8H_{17}O_2BrNa^+$ ($[M+Na]^+$): 247.03041, found 247.03036.

5,6-bis(hydroxymethyl)bicyclo[2.2.1]heptane-2,3-diol (N2)

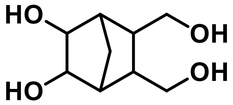

N2

Following *GP-B*, the title compound was prepared from bicyclo[2.2.1]hept-5-ene-2,3-diyldimethanol (30.842 mg, 0.2 mmol, 1.0 equiv.). Purification by column chromatography on silica gel (CH_2Cl_2/MeOH: 50/1 to 12/1) afforded the title compound as a colourless oil in 50:50 diastereomeric ratio (21.8 mg, 0.116 mmol, 58%). Combined NMR data for both isomers are reported below.

R_f = 0.42 (CH_2Cl_2/MeOH = 10/1)

^{1}H NMR (400 MHz, MeOD) δ 3.91 (d, *J* = 1.7 Hz, 2H), 3.79 – 3.69 (m, 2H), 3.59 – 3.50 (m, 2H), 2.24 – 2.17 (m, 4H), 1.95 – 1.88 (m, 1H), 1.23 – 1.16 (m, 1H).

^{13}C NMR (101 MHz, MeOD) δ 70.07, 60.25, 48.41, 42.67, 34.03.

HRMS (ESI): calculated for $C_{19}H_{16}O_4Na^+$ ($[M+Na]^+$): 211. 09408, found 211.09394.

(2R,3R,4R)-2-(acetoxymethyl)-5,6-dihydroxytetrahydro-2H-pyran-3,4-diyl diacetate (O2)

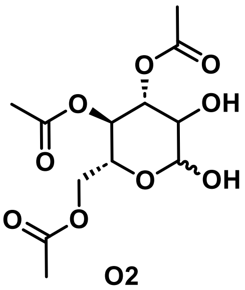

O2

Following *GP-B*, the title compound was prepared from (2R,3S,4R)-2-(acetoxymethyl)-3,4-dihydro-2H-pyran-3,4-diyl diacetate (54.5 mg, 0.2 mmol, 1.0 equiv.). Purification by column chromatography on silica gel (CH_2Cl_2/MeOH: 100/1 to 25/1) afforded the title compound in 72:28 diastereomeric ratio (13.7 mg, 0.05 mmol, 25%) as a colorless oil. Combined NMR data for both the isomers are reported below.

R_f = 0.4 (CH_2Cl_2/MeOH = 25/1).

^{1}H NMR (500 MHz, $CDCl_3$) δ 5.45 – 5.35 (m, 1H), 4.90 – 4.78 (m, 1H), 4.51 – 4.40 (m, 1H), 4.39 – 4.30 (m, 1H), 4.11 (ddd, *J* = 10.1, 4.3, 2.3 Hz, 1H), 3.61 – 3.55 (m, 1H), 3.24 (dd, *J* = 3.8, 1.2 Hz, 1H), 3.07 (dd, *J* = 22.0, 4.9 Hz, 1H), 2.13 (d, *J* = 3.7 Hz, 3H), 2.11 (d, *J* = 1.1 Hz, 3H), 2.09 (d, *J* = 0.7 Hz, 3H).

^{13}C NMR (101 MHz, $CDCl_3$) δ 171.85, 171.75, 171.68, 171.35, 171.10, 170.26, 95.58, 90.37, 74.70, 74.46, 73.23, 72.54, 70.90, 69.95, 69.31, 69.05, 62.94, 62.88, 20.91, 20.88, 20.83, 20.76.

HRMS (ESI): calculated for $C_{12}H_{18}O_9Na^+$ ($[M+Na]^+$): 329.08430, found: 329.08402.

Methyl 3-((diphenylmethylene)amino)-3-(1H-1,2,4-triazol-1-yl)propanoate (A3)

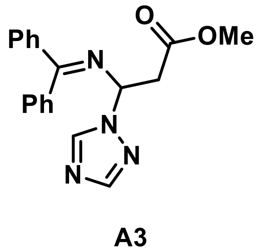

A3

Following *GP-A*, the title compound was prepared from 1-vinyl-1H-1,2,4-triazole (19.1 mg, 0.2 mmol, 1.0 equiv.). Purification by column chromatography on silica gel (n-pentane/EtOAc: 20/1 to 1/1) afforded the title compound (16 mg, 0.048 mmol, 24%) as a yellow oil.

R_f = 0.45 (n-pentane/EtOAc = 1/1).

^{1}H NMR (400 MHz, $CDCl_3$) δ 8.22 (s, 1H), 7.95 (s, 1H), 7.70 – 7.62 (m, 2H), 7.55 – 7.48 (m, 3H), 7.47 – 7.42 (m, 1H), 7.35 (m, *J* = 8.3, 6.9 Hz, 2H), 7.20 – 7.13 (m, 2H), 6.09 (t, *J* = 6.1 Hz, 1H), 3.60 (s, 3H), 3.23 (dd, *J* = 15.8, 6.1 Hz, 1H), 3.06 (dd, *J* = 15.8, 6.2 Hz, 1H).

^{13}C NMR (101 MHz, $CDCl_3$) δ 173.17, 169.45, 151.92, 142.28, 138.42, 135.15, 131.63, 130.19, 129.64, 129.37, 129.08, 128.40, 127.48, 77.48, 77.16, 76.84, 72.94, 52.05, 41.52.

HRMS (ESI): calculated for $C_{19}H_{18}N_4O_2Na^+$ ($[M+Na]^+$): 357.13220, found: 357.13198.

1,1-diethyl 5-methyl 1-((diphenylmethylene)amino)pent-3-ene-1,1,5-tricarboxylate (B3)

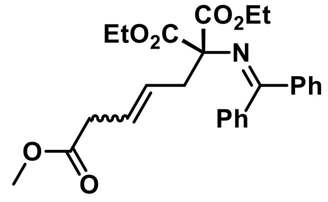

B3

Following *GP-A*, the title compound was prepared from diethyl-2-vinylcyclopropane-1,1-dicarboxylate (42.4 mg, 0.2 mmol, 1.0 equiv.). Due to the radical reaction pathway, the cyclopropane undergoes ring opening. Purification by column chromatography on silica gel (n-pentane/EtOAc: 33/1 to 8/1) afforded the title compound in 78:22 *E:Z* isomeric ratio (20.7 mg, 0.046 mmol, 23%) as a yellow oil. Combined NMR data for both the isomers are reported below.

R_f = 0.15 (n-pentane/EtOAc = 10/1).

^{1}H NMR (400 MHz, $CDCl_3$) δ 7.63 – 7.57 (m, 2H), 7.41 – 7.34 (m, 4H), 7.33 – 7.28 (m, 2H), 7.22 (m, *J* = 6.1, 2.8, 1.7 Hz, 2H), 5.93 – 5.58 (m, 2H), 4.04 – 3.80 (m, 4H), 3.65 (m, *J* = 8.4 Hz, 3H), 3.11 (m, *J* = 30.5, 7.0, 1.5 Hz, 2H), 2.94 – 2.83 (m, 2H), 1.16 (m, *J* = 7.1, 2.0 Hz, 6H).

^{13}C NMR (101 MHz, $CDCl_3$) δ 172.29, 169.61, 169.54, 169.23, 140.94, 136.41, 130.58, 130.55, 129.68, 129.62, 129.13, 129.11, 128.97, 128.80, 128.77, 128.07, 128.00, 127.77, 127.20, 126.09, 124.33, 77.48, 77.16, 76.84, 75.11, 74.70, 61.66, 61.56, 51.93, 51.84, 42.19, 38.10, 33.15, 13.97, 13.93.

HRMS (ESI): calculated for $C_{26}H_{29}NO_6Na^+$ ($[M+Na]^+$): 474.18871, found: 474.18895.

Methyl 2-(5-((diphenylmethylene)amino)-10,11-dihydro-5H-dibenzo[a,d][7]annulen-5-yl)-4-(N-methylpivalamido)butanoate (E3)

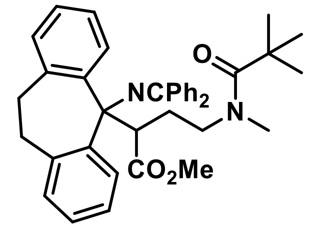

E3

Following *GP-A*, the title compound was prepared from N-(3-(10,11-dihydro-5H-dibenzo[a,d][7]annulen-5-ylidene)propyl)-N-methylpivalamide (69.5 mg, 0.2 mmol, 1.0 equiv.). Purification by column chromatography on silica gel (n-pentane/EtOAc: 20/1 to 8/1) afforded the title compound (14 mg, 0.024 mmol, 12%) as a yellow oil.

R$_f$ = 0.35 (n-pentane/EtOAc = 10/1).

^{1}H NMR (400 MHz, $CDCl_3$) δ 7.70 – 7.61 (m, 2H), 7.52 – 7.45 (m, 2H), 7.42 – 7.30 (m, 4H), 7.15 – 6.92 (m, 6H), 6.82 – 6.73 (m, 2H), 6.03 – 5.83 (m, 2H), 3.72 – 3.66 (m, 1H), 3.49 (s, 3H), 3.46 – 3.38 (m, 1H), 3.1 – 3.01 (m, 1H), 2.81 (s, 3H), 2.60 (dd, *J* = 15.4, 9.3 Hz, 1H), 2.29 – 2.19 (m, 1H), 2.14 – 1.97 (m, 2H), 1.27 – 1.22 (m, 2H), 1.18 (s, 9H).

^{13}C NMR (101 MHz, $CDCl_3$) δ 177.26, 173.39, 168.43, 143.44, 143.00, 142.28, 141.84, 141.35, 138.42, 132.33, 131.84, 130.14, 129.37, 129.24, 128.51, 128.02, 127.43, 127.15, 126.81, 126.70, 126.67, 125.51, 125.34, 74.57, 61.91, 51.41, 48.88, 38.78, 36.17, 36.00, 28.36, 28.30, 28.22.

HRMS (ESI): calculated for $C_{39}H_{42}N_2O_3Na^+$ ($[M+Na]^+$): 609.30876, found: 609.30922.

4-methyl 1-(2,2,2-trifluoroethyl) 2-((diphenylmethylene)amino)succinate (K3)

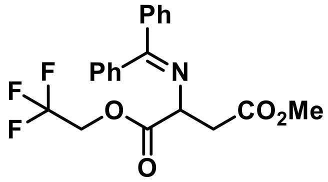

K3

Following *GP-A*, the title compound was prepared from 2,2,2-trifluoroethyl acrylate (30.82 mg, 0.2 mmol, 1.0 equiv.). Purification by column chromatography on silica gel (n-pentane/EtOAc: 33/1 to 8/1) afforded the title compound (13.37 mg, 0.034 mmol, 17%) as a yellow oil.

R$_f$ = 0.33 (n-pentane/EtOAc = 10/1).

^{1}H NMR (400 MHz, $CDCl_3$) δ 7.63 – 7.58 (m, 2H), 7.47 (dd, *J* = 5.1, 1.9 Hz, 3H), 7.41 – 7.39 (m, 1H), 7.34 (dd, *J* = 7.8, 1.5 Hz, 2H), 7.25 – 7.21 (m, 2H), 4.63 (dd, *J* = 7.1, 6.0 Hz, 1H), 4.57 – 4.42 (m, 2H), 3.65 (s, 3H), 3.09 (dd, *J* = 16.5, 5.9 Hz, 1H), 2.88 (dd, *J* = 16.4, 7.1 Hz, 1H).

^{13}C NMR (101 MHz, $CDCl_3$) δ 172.97, 170.86, 169.45, 139.13, 135.77, 130.77, 130.07, 129.45, 129.39, 129.15, 129.02, 128.98, 128.59, 128.35, 128.29, 128.26, 128.12, 127.83, 127.80, 127.74, 126.46, 126.19, 61.41, 61.37, 61.05, 60.68, 60.32, 51.83, 37.70.

^{19}F NMR (376 MHz, $CDCl_3$) δ -73.63.

HRMS (ESI): calculated for $C_{20}H_{18}NO_4F_3Na^+$ ($[M+Na]^+$): 416.10801, found: 416.10763.

Methyl 9-bromo-3-((diphenylmethylene)amino)nonanoate (L3)

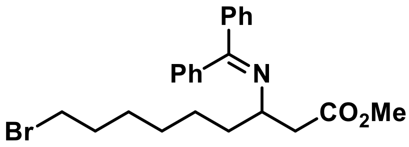

L3

Following *GP-A*, the title compound was prepared from 8-bromooct-1-ene (38.22 mg, 0.2 mmol, 1.0 equiv.). Purification by column chromatography on silica gel (n-pentane/EtOAc: 200/1 to 100/1) afforded the title compound (33.57 mg, 0.077 mmol, 39%) as a yellow oil.

R_f = 0.45 (n-pentane/EtOAc = 20/1).

^{1}H NMR (400 MHz, $CDCl_3$) δ 7.62 – 7.54 (m, 2H), 7.50 – 7.41 (m, 3H), 7.39 – 7.28 (m, 3H), 7.22 – 7.16 (m, 2H), 3.83 – 3.75 (m, 1H), 3.61 (s, 3H), 3.36 (t, *J* = 6.8 Hz, 2H), 2.72 – 2.49 (m, 2H), 1.84 – 1.73 (m, 2H), 1.63 – 1.50 (m, 2H), 1.40 – 1.32 (m, 2H), 1.27 – 1.13 (m, 4H).

^{13}C NMR (101 MHz, $CDCl_3$) δ 172.40, 167.72, 140.09, 136.93, 129.91, 128.53, 128.34, 128.31, 128.04, 127.96, 58.48, 51.44, 41.15, 36.18, 33.96, 32.74, 28.68, 28.04, 25.89.

HRMS (ESI): calculated for $C_{23}H_{28}NO_2BrNa^+$ ($[M+Na]^+$): 452.11956, found: 452.11989.

Methyl 2-((diphenylmethylene)amino)-5,5-dimethyl-3-oxocyclohex-1-ene-1-carboxylate (M3)

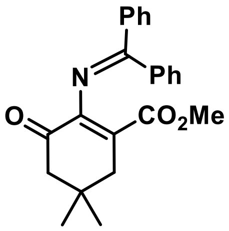

M3

Following *GP-A*, the title compound was prepared from 3-chloro-5,5-dimethylcyclohex-2-en-1-one (31.72 mg, 0.2 mmol, 1.0 equiv.). Purification by column chromatography on silica gel (n-pentane/EtOAc: 20/1 to 10/1) afforded the title compound (30 mg, 0.083 mmol, 42%) as a yellow oil.

R_f = 0.4 (n-pentane/EtOAc = 10/1).

^{1}H NMR (400 MHz, $CDCl_3$) δ 7.59 – 7.53 (m, 4H), 7.48 – 7.38 (m, 6H), 3.73 (s, 3H), 2.21 (s, 2H), 2.16 (s, 2H), 0.80 (s, 6H).

^{13}C NMR (101 MHz, $CDCl_3$) δ 194.06, 167.98, 166.08, 164.76, 136.51, 130.86, 129.08, 128.40, 115.59, 51.92, 50.84, 43.51, 32.33, 29.71, 27.93.

HRMS (ESI): calculated for $C_{23}H_{23}NO_3Na^+$ ($[M+Na]^+$): 384.15701, found: 384.15668.

Methyl 3-((diphenylmethylene)amino)-5,6-bis(hydroxymethyl)bicyclo[2.2.1]heptane-2-carboxylate (N3)

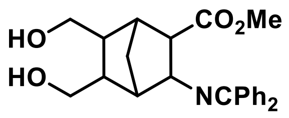

N3

Following *GP-A*, the title compound was prepared from bicyclo[2.2.1]hept-5-ene-2,3-diyldimethanol (30.842 mg, 0.2 mmol, 1.0 equiv.). Purification by column chromatography on silica gel (n-pentane/EtOAc: 50/1 to 1/1) afforded the title compound in 50:50 diastereomeric ratio (44 mg, 0.112 mmol, 56%) as a yellow oil. Combined NMR data for both the isomers are reported below.

R_f = 0.25 (n-pentane/EtOAc = 5/1).

^{1}H NMR (400 MHz, $CDCl_3$) δ 7.50 – 7.34 (m, 5H), 7.34 – 7.18 (m, 3H), 7.12 – 6.99 (m, 2H), 4.21 – 4.07 (m, 1H), 3.99 – 3.90 (m, 1H), 3.78 – 3.69 (m, 1H), 3.53 – 3.37 (m, 4H), 3.15 – 2.88 (m, 1H), 2.68 – 2.48 (m, 2H), 2.38 – 2.27 (m, 1H), 2.24 – 2.03 (m, 2H), 1.61 – 1.27 (m, 2H).

^{13}C NMR (101 MHz, $CDCl_3$) δ 175.02, 172.93, 170.44, 166.67, 139.24, 138.88, 136.59, 136.25, 134.82, 130.65, 130.12, 128.80, 128.74, 128.65, 128.51, 128.46, 128.39, 128.35, 128.06, 127.67, 127.60, 66.95, 63.56, 61.53, 61.41, 61.33, 60.86, 60.52, 51.60, 51.39, 49.27, 49.24, 47.17, 47.02, 46.59, 45.29, 45.20, 43.99, 43.28, 43.13, 43.06, 42.39, 38.91, 36.70.

HRMS (ESI): calculated for $C_{24}H_{27}NO_4Na^+$ ($[M+Na]^+$): 416.18323, found: 416.18343.

11. Copies of ^{1}H and ^{13}C NMR

^{1}H NMR (400 MHz, MeOD) for **C2**

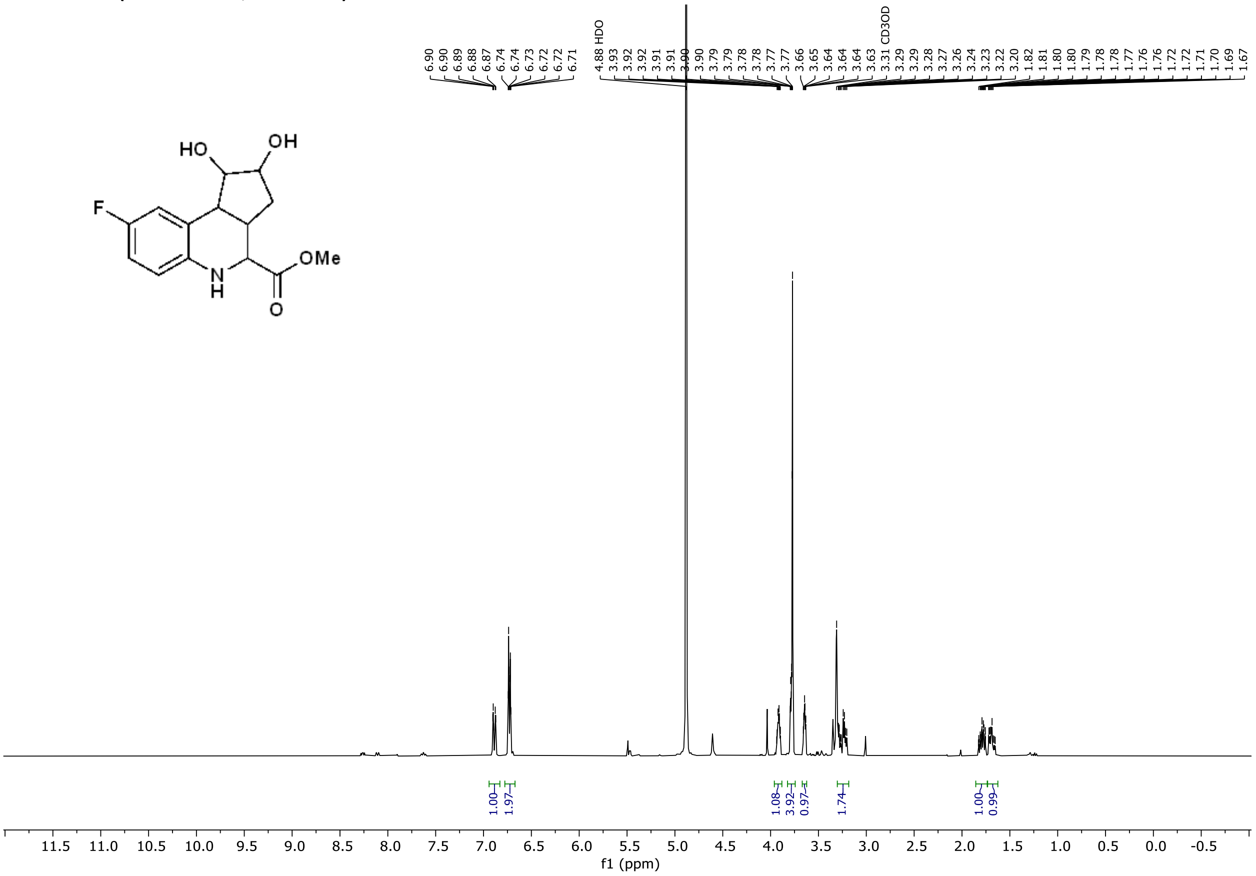

^{13}C NMR (101 MHz, MeOD) for **C2**

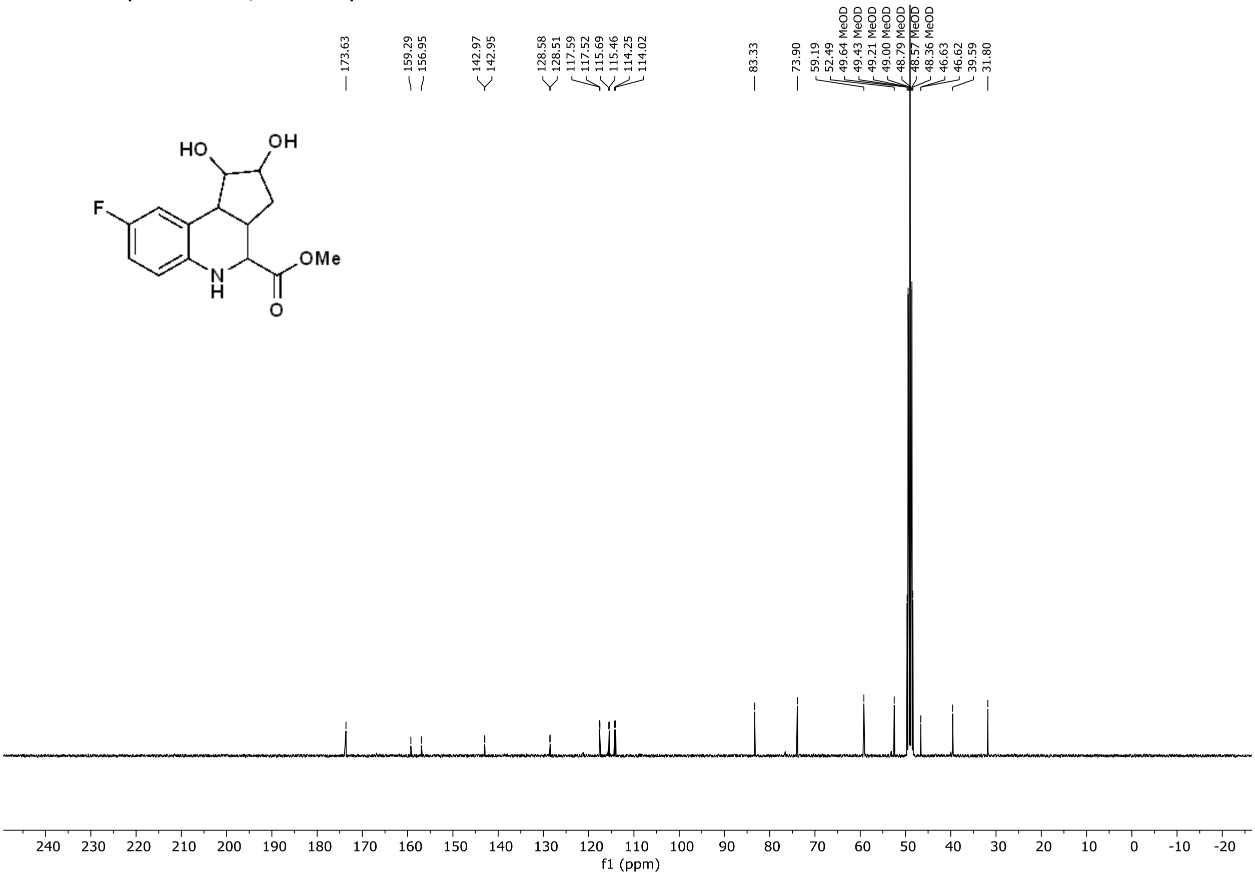

^{1}H NMR (400 MHz, $CDCl_3$) for **D2**

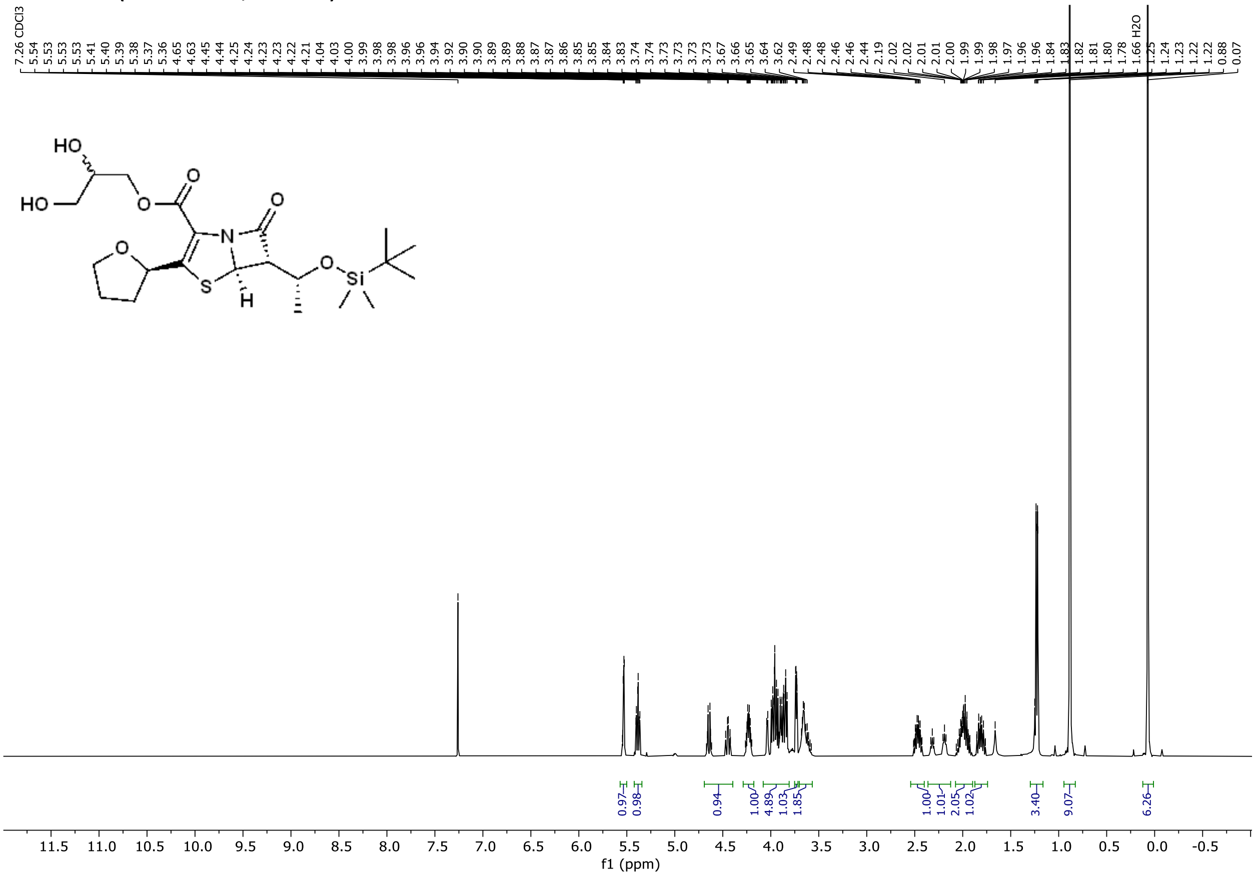

^{13}C NMR (101 MHz, $CDCl_3$) for **D2**

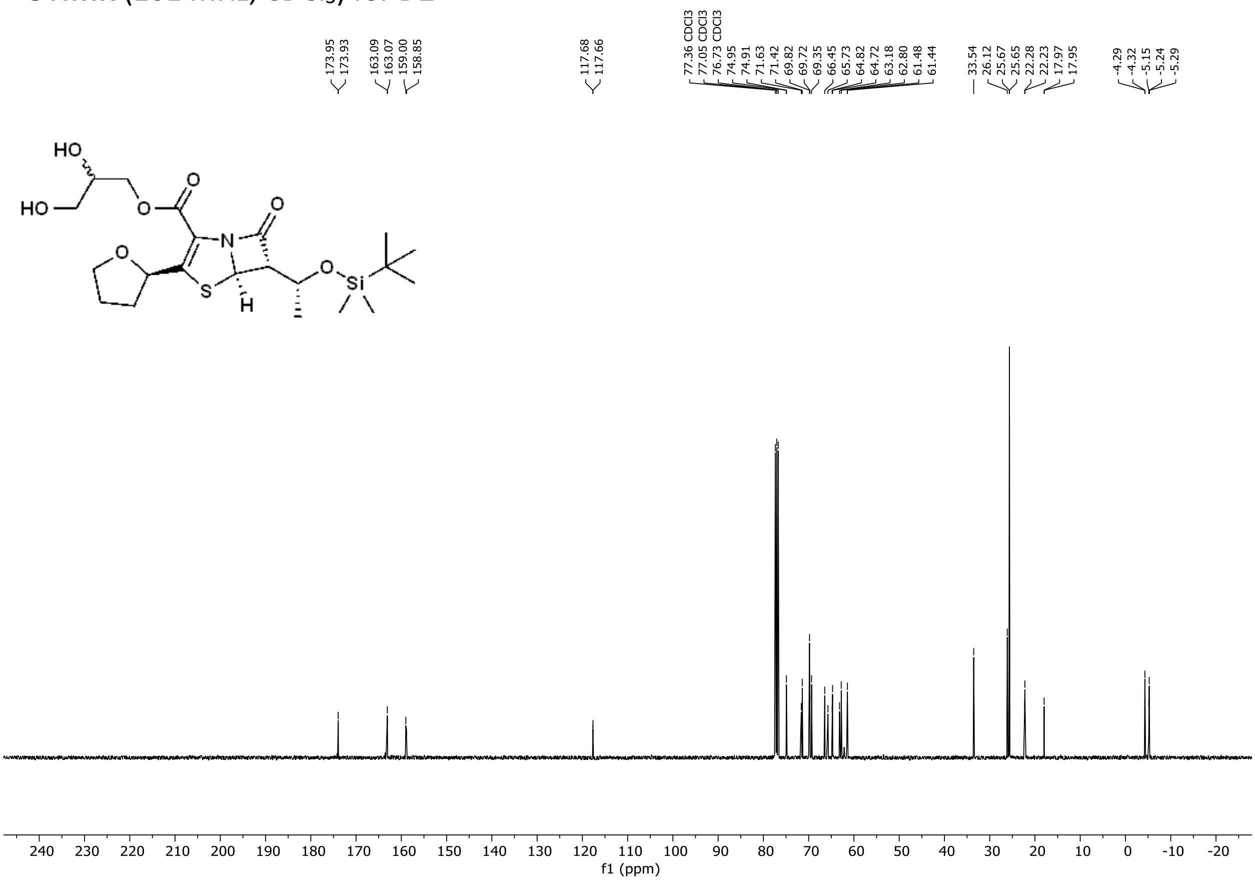

^{1}H NMR (400 MHz, $CDCl_3$) for **E2**

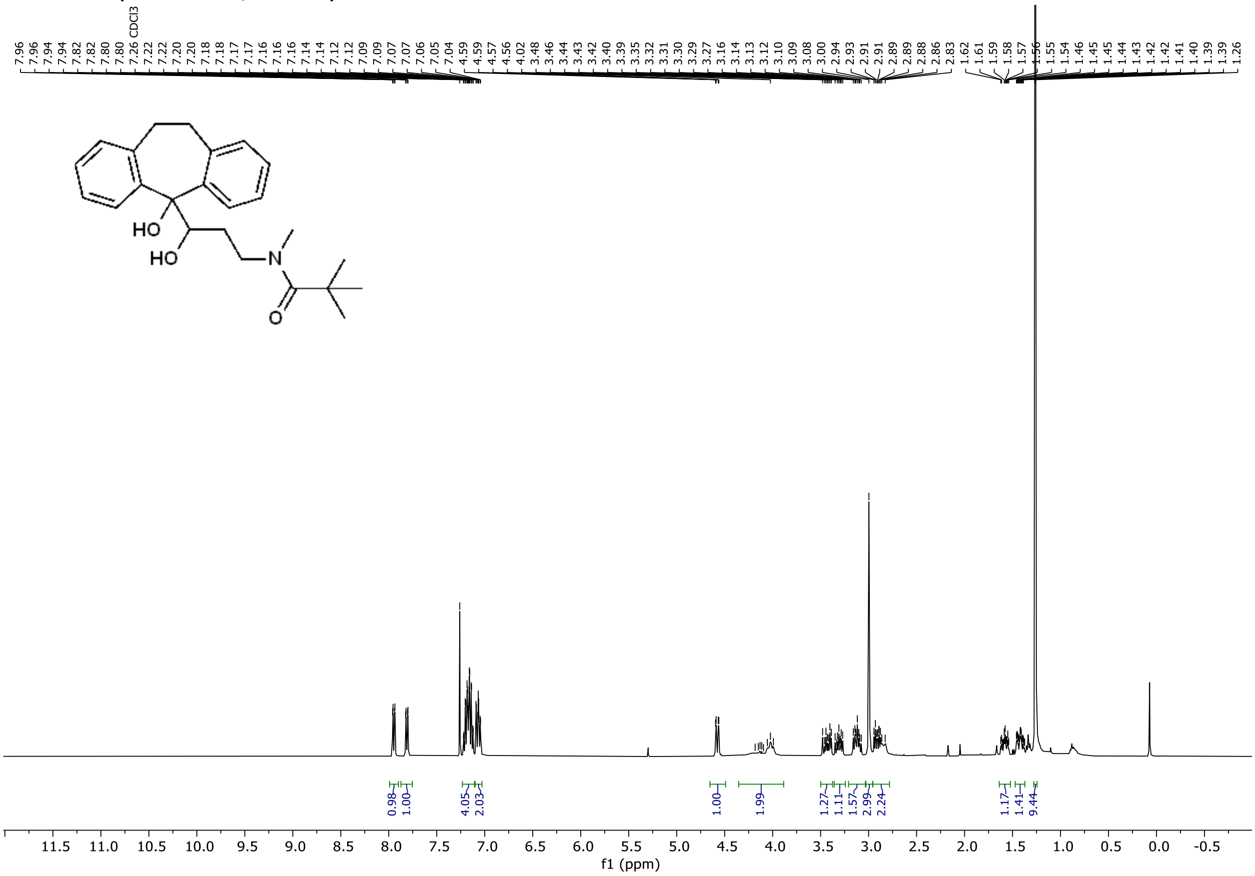

^{13}C NMR (101 MHz, $CDCl_3$) for **E2**

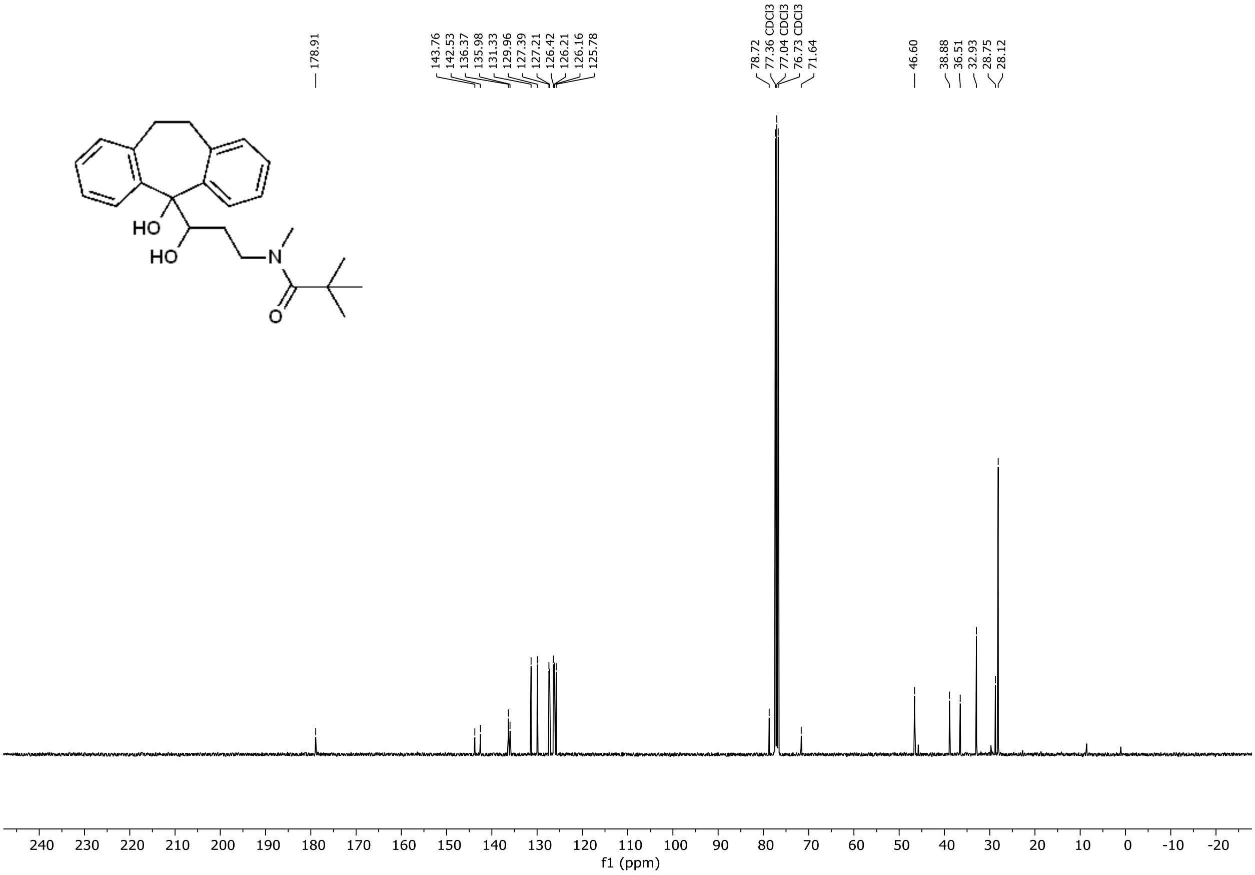

^{1}H NMR (400 MHz, $CDCl_3$) for **F2**

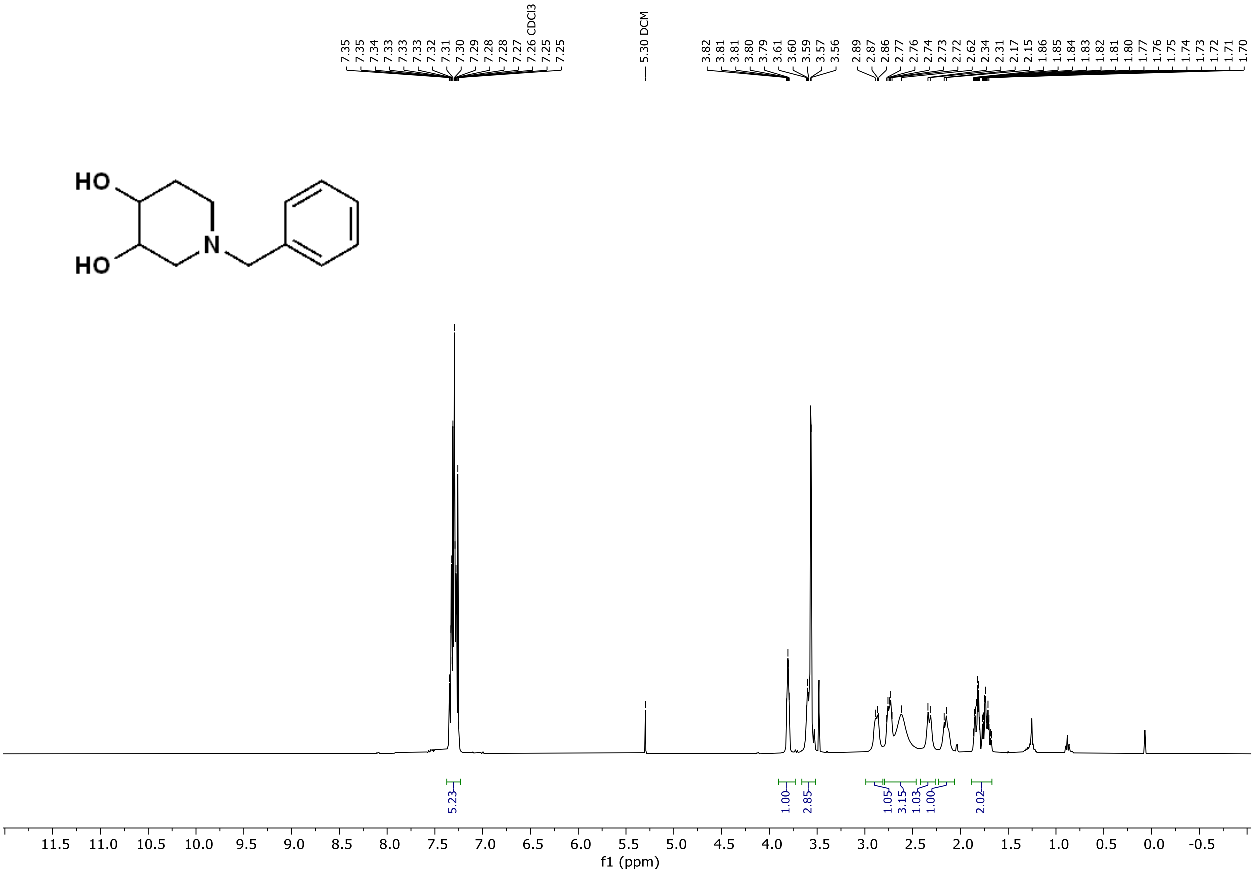

^{13}C NMR (101 MHz, $CDCl_3$) for **F2**

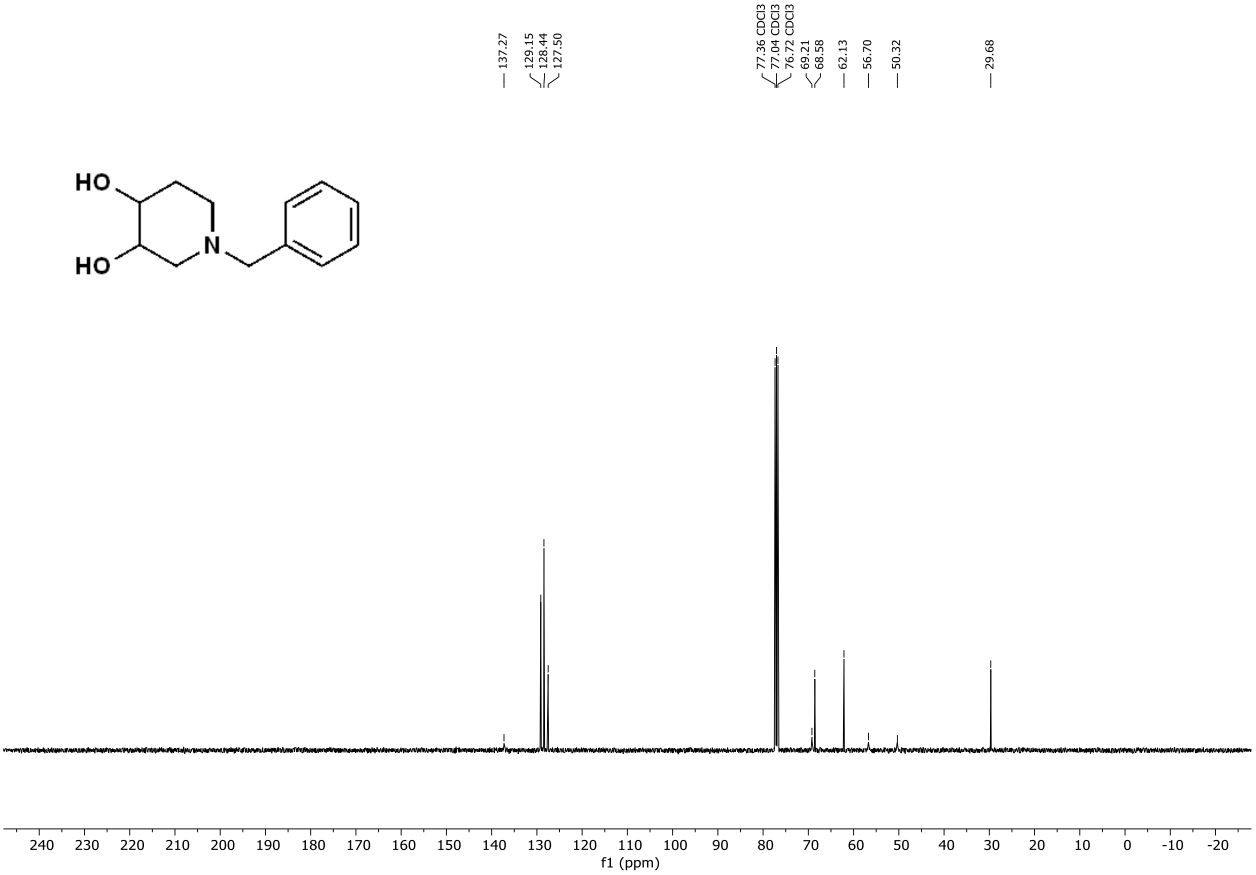

^{1}H NMR (400 MHz, MeOD) for **H2**

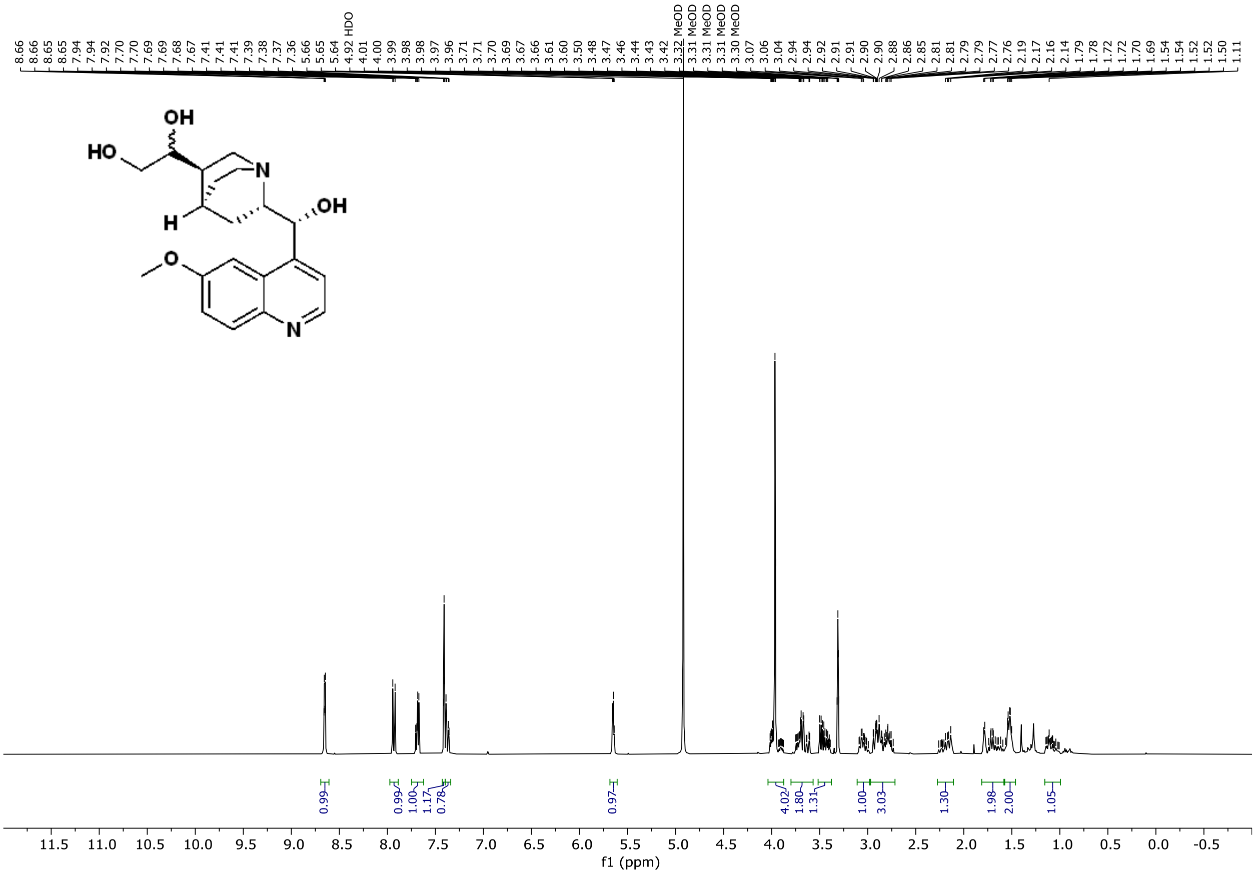

^{13}C NMR (101 MHz, MeOD) for **H2**

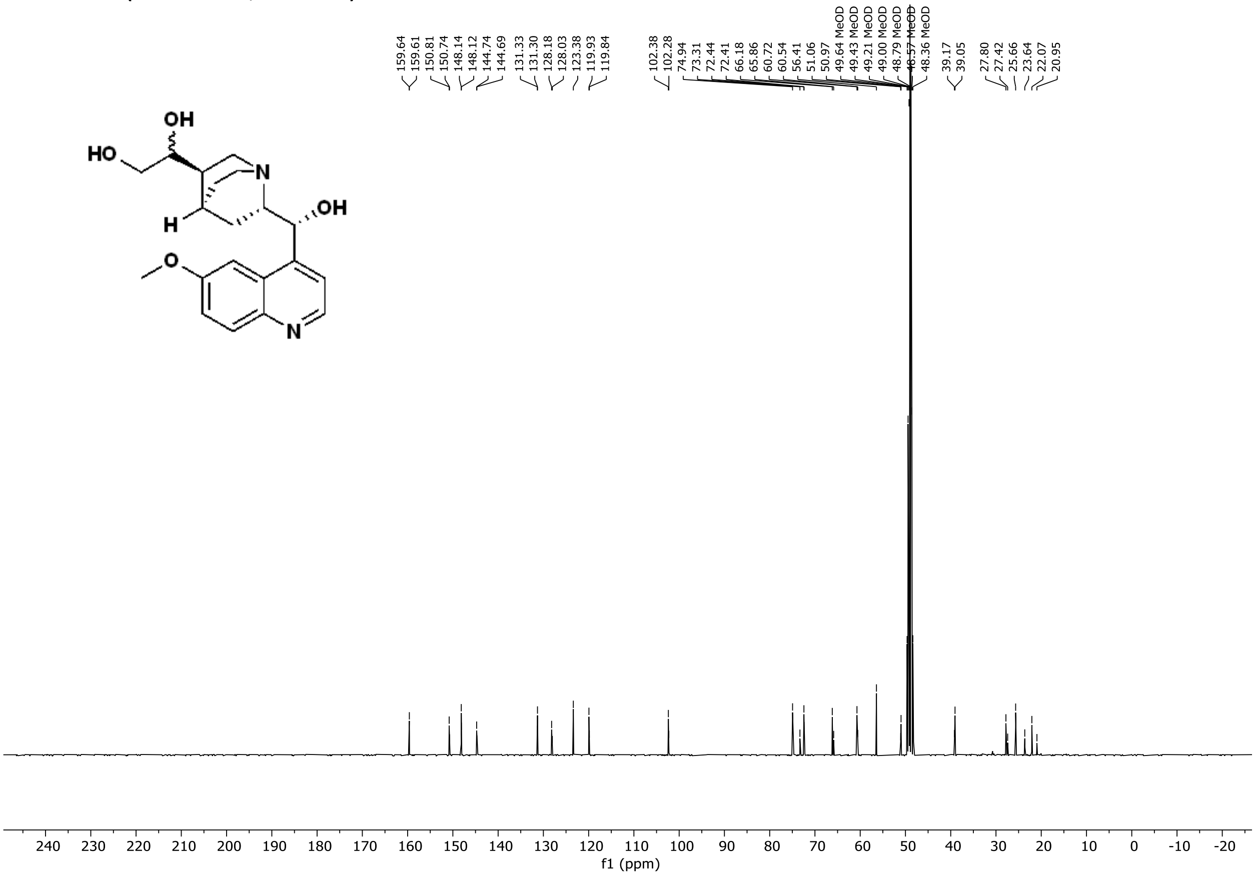

^{1}H NMR (400 MHz, $CDCl_3$) for **L2**

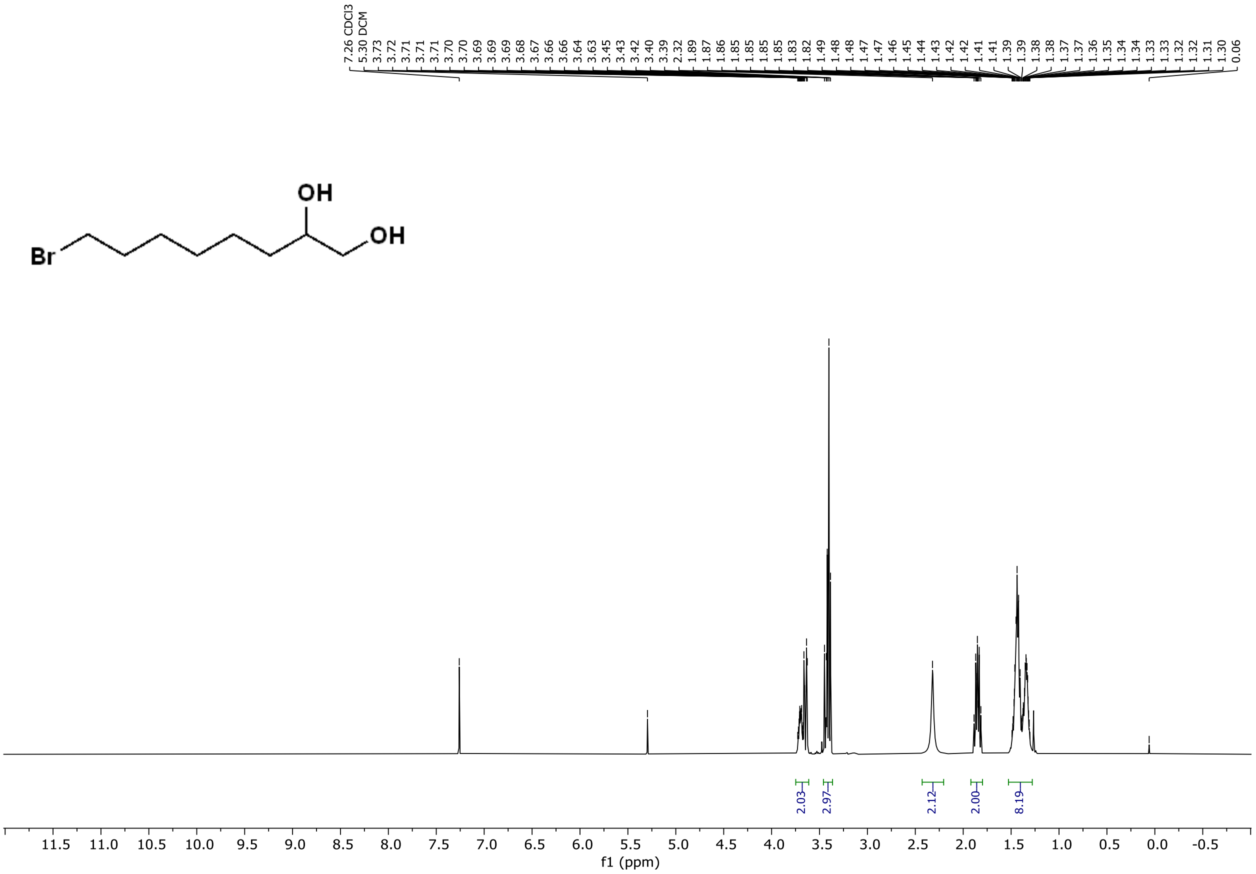

^{13}C NMR (101 MHz, $CDCl_3$) for **L2**

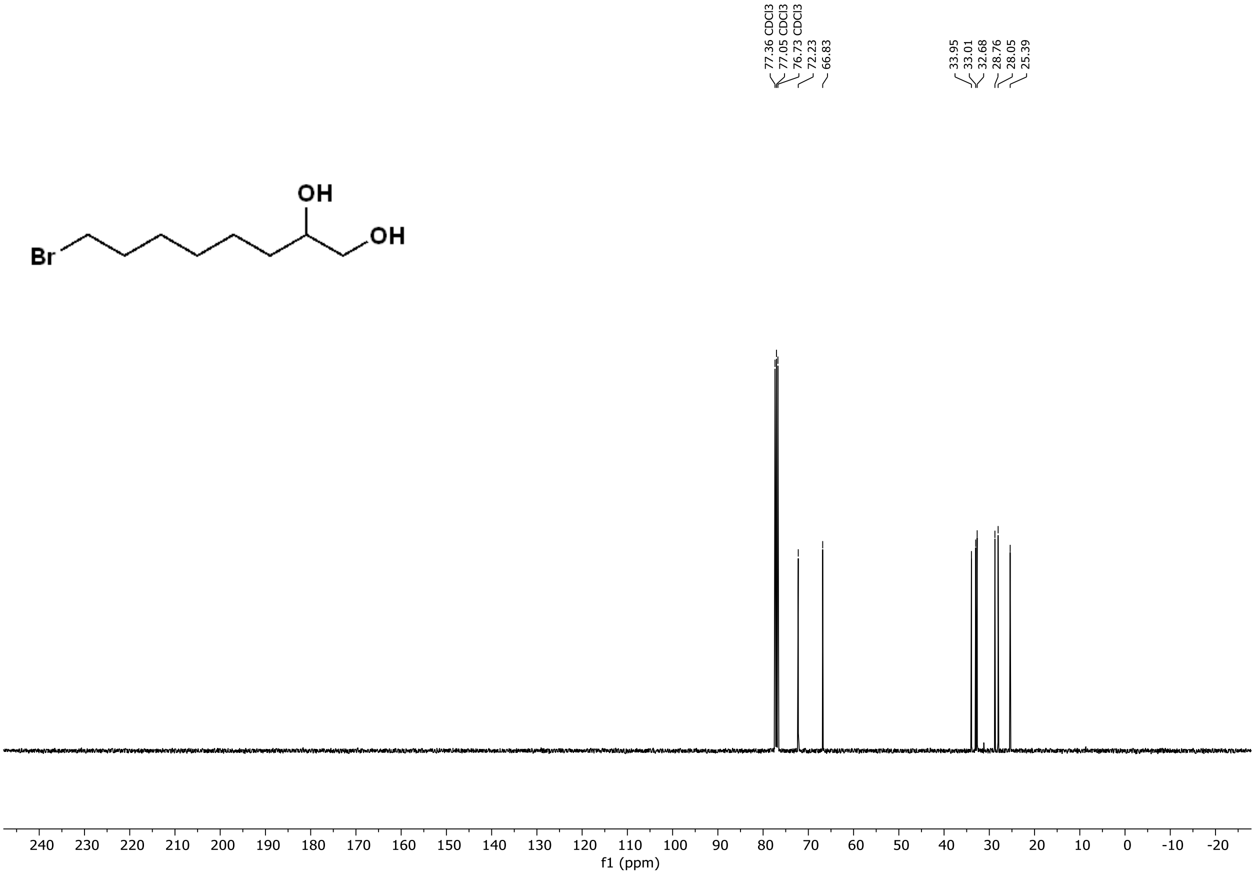

^{1}H NMR (400 MHz, MeOD) for **N2**

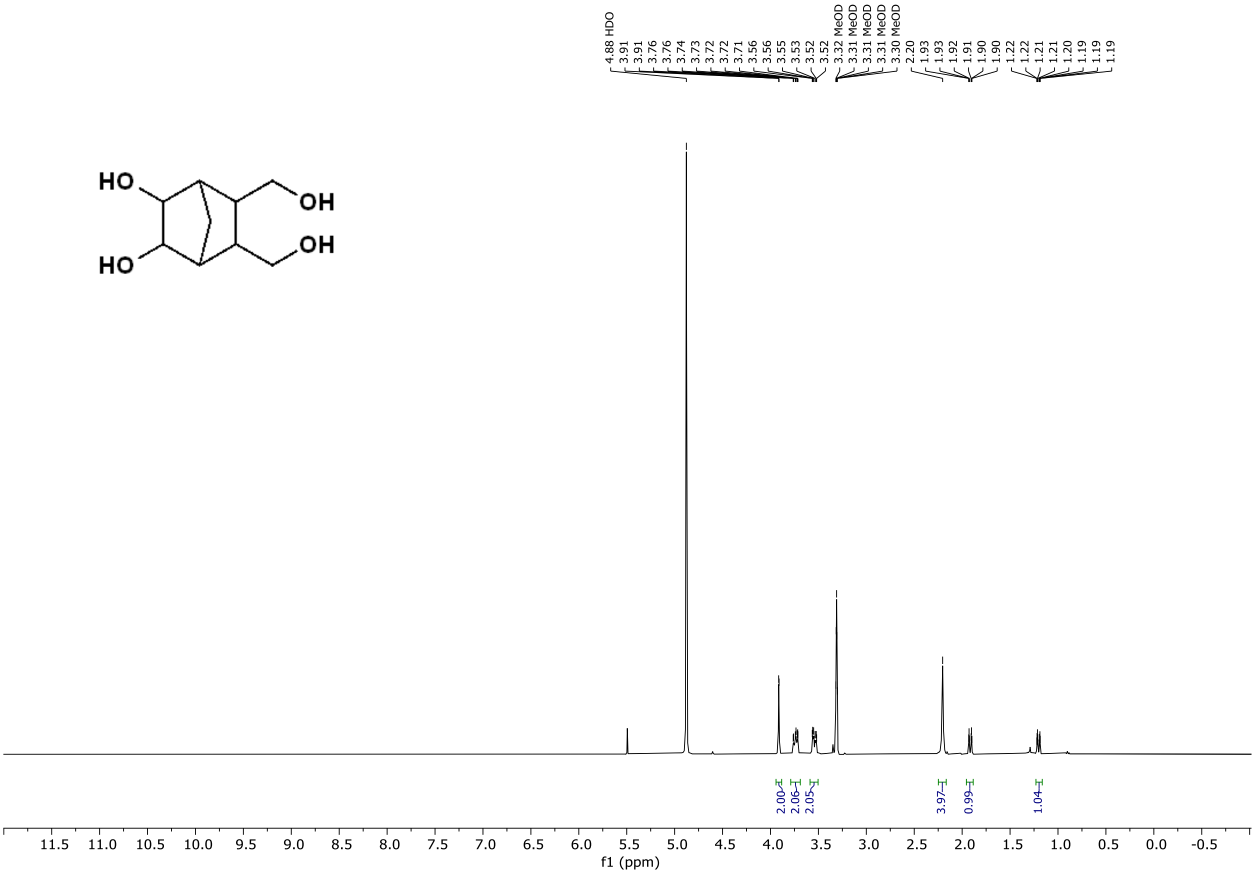

^{13}C NMR (101 MHz, MeOD) for **N2**

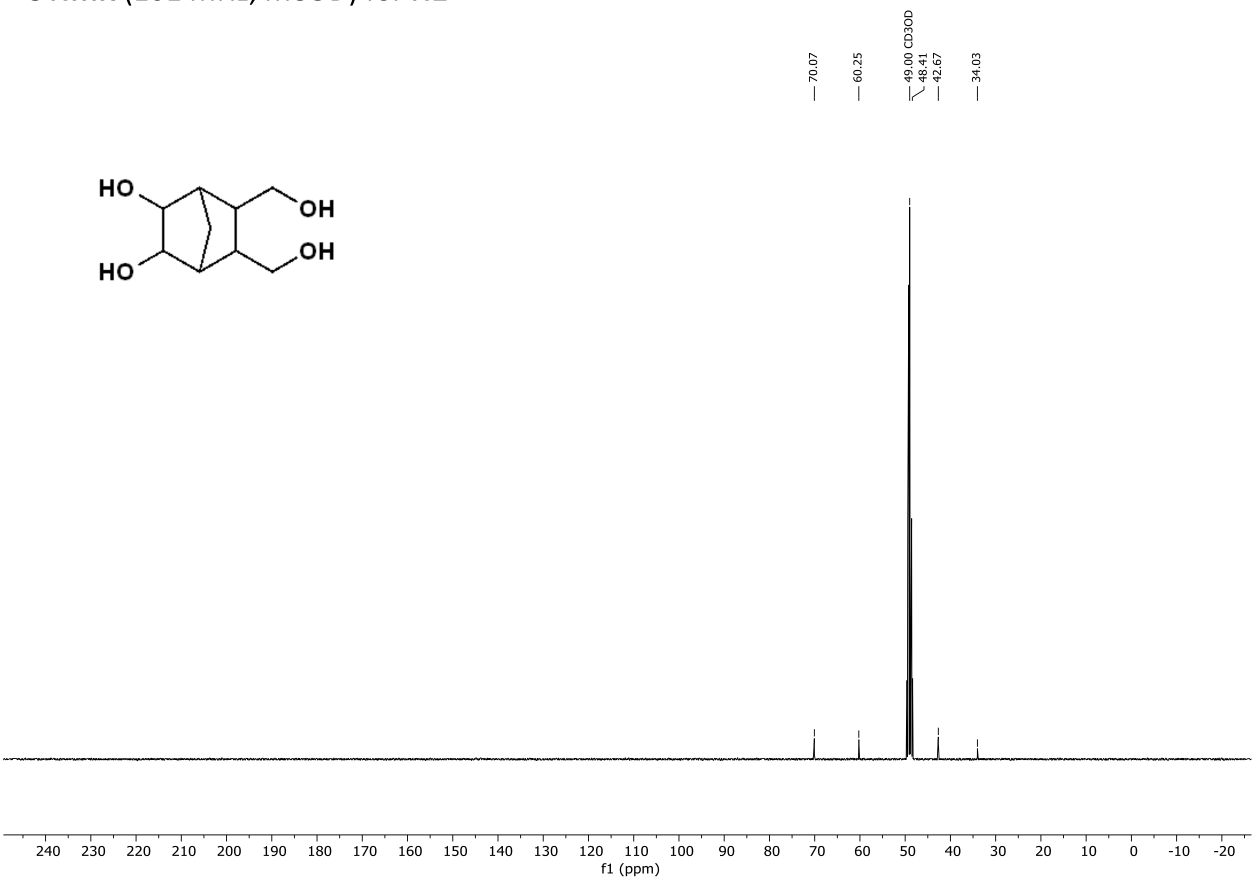

^{1}H NMR (500 MHz, $CDCl_3$) for **O2**

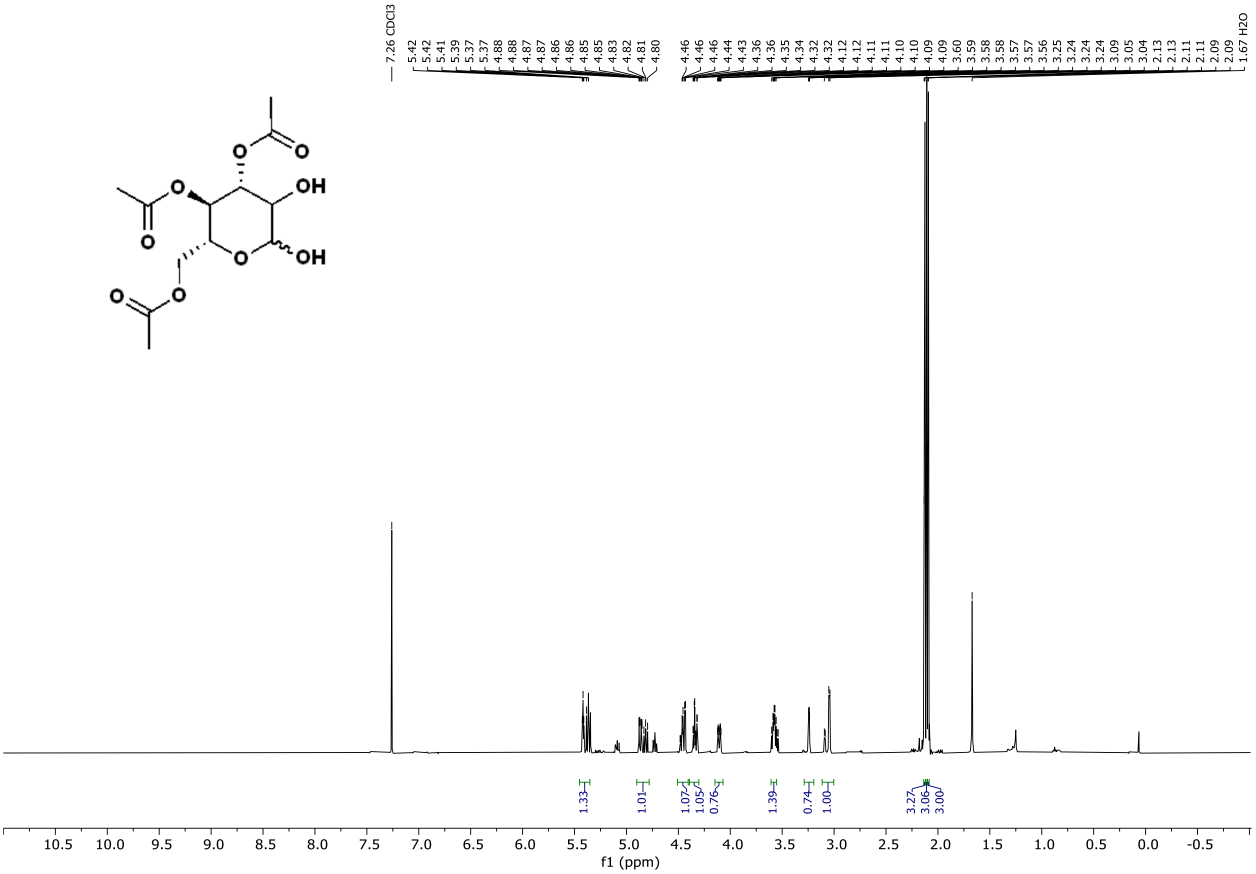

^{13}C NMR (101 MHz, $CDCl_3$) for **O2**

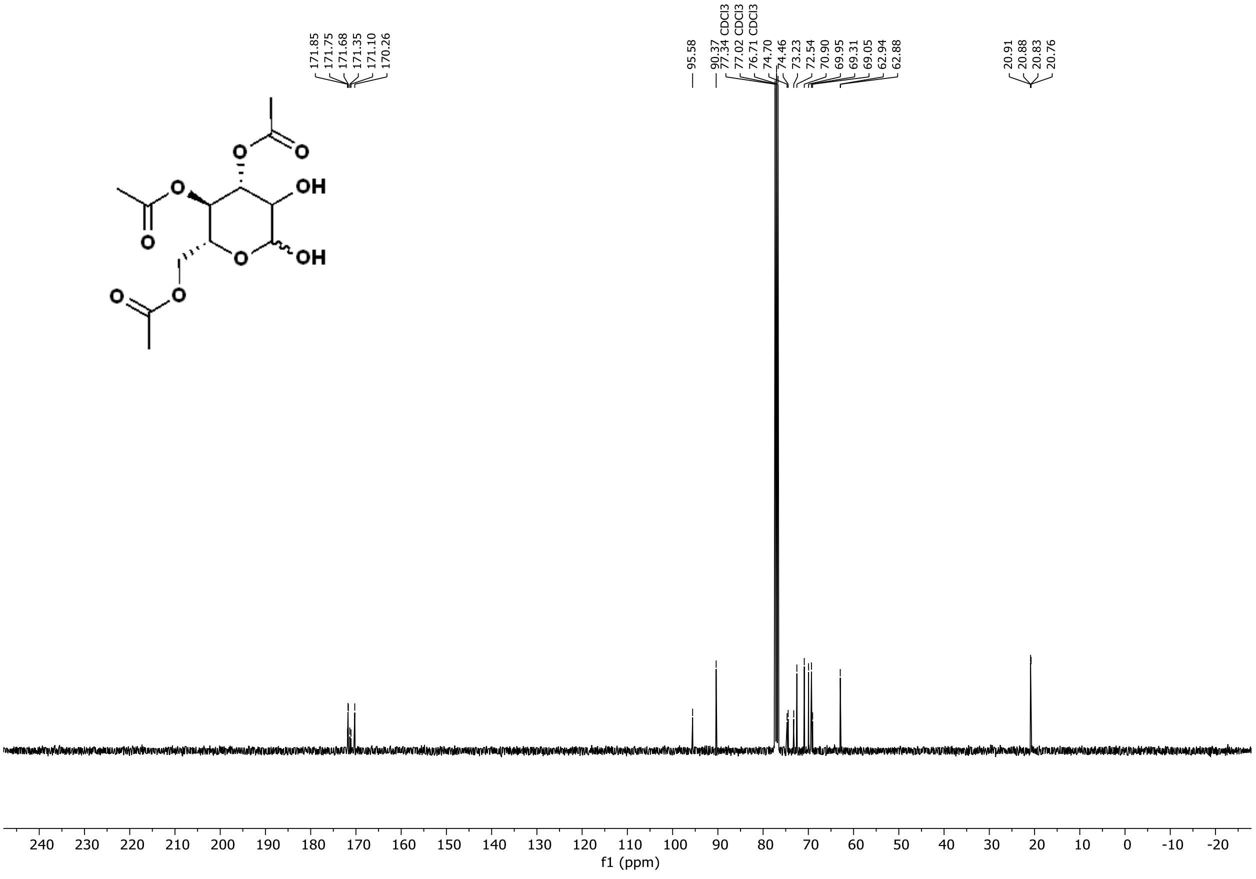

^{1}H NMR (400 MHz, $CDCl_3$) for **A3**

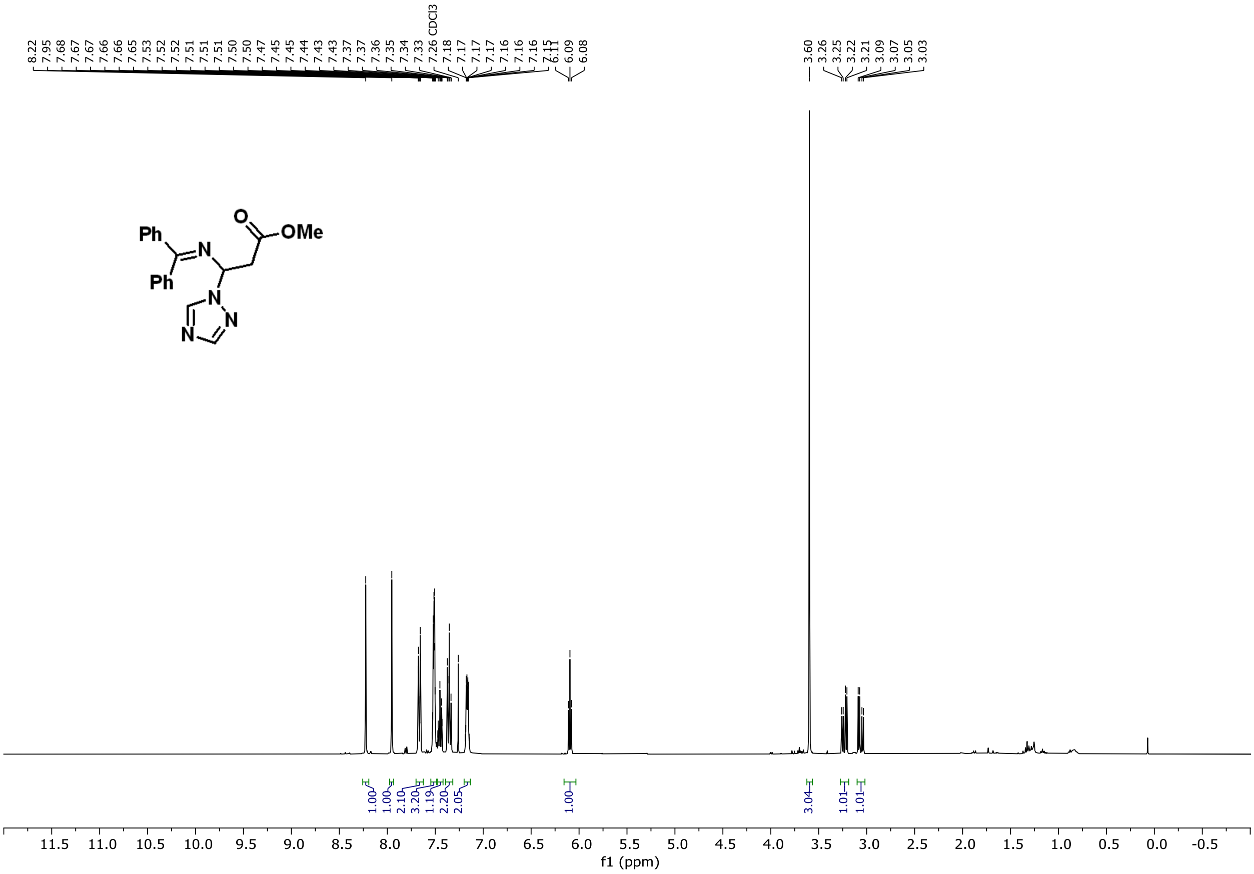

^{13}C NMR (101 MHz, $CDCl_3$) for **A3**

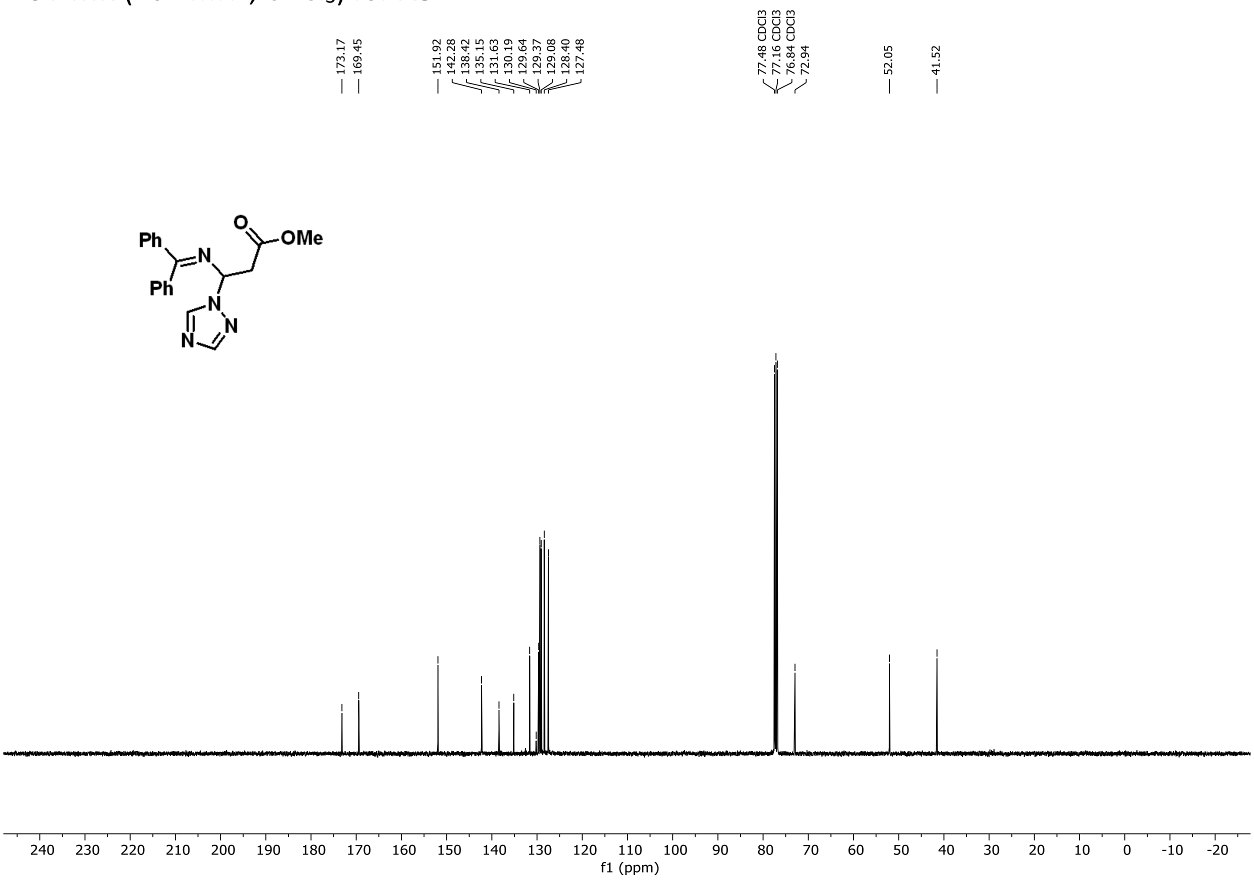

^{1}H NMR (400 MHz, $CDCl_3$) for **B3**

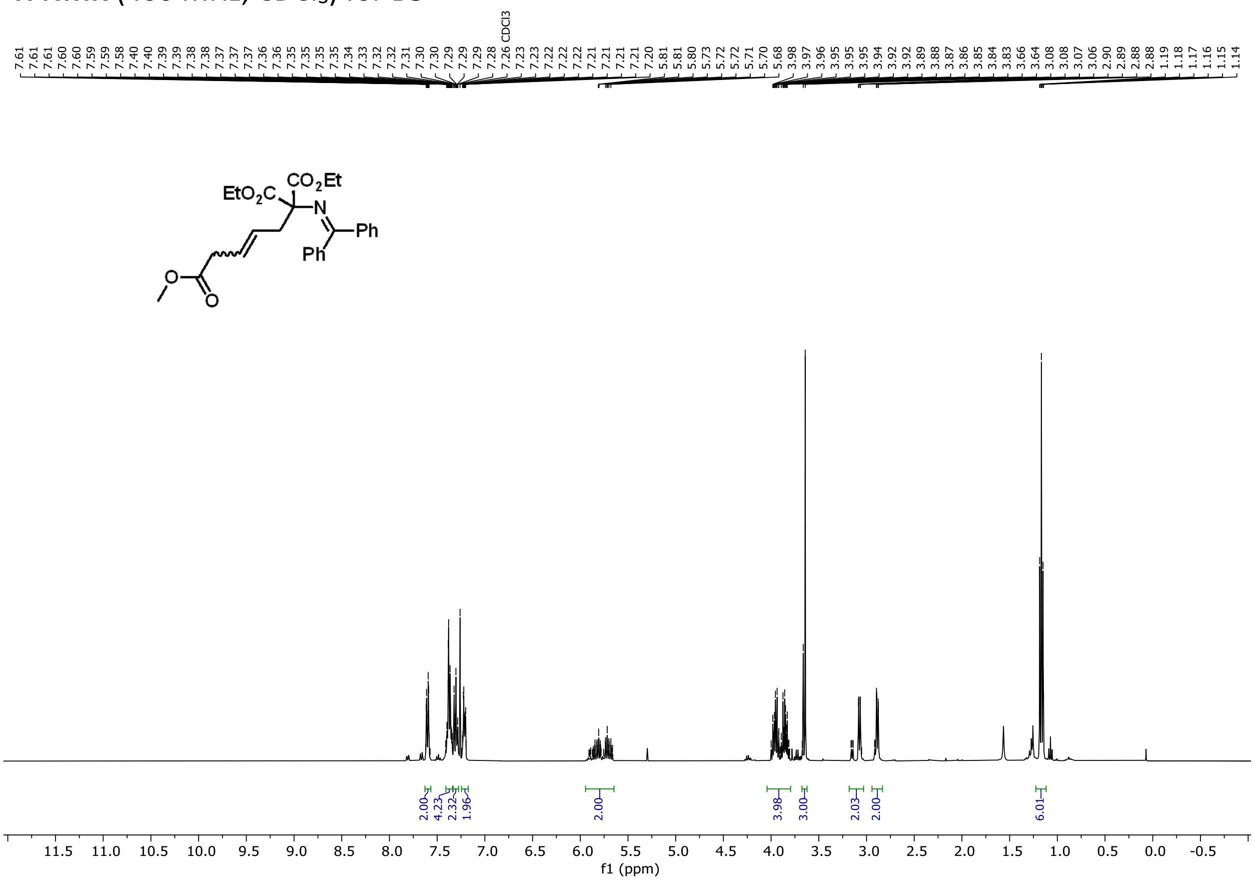

^{13}C NMR (101 MHz, $CDCl_3$) for **B3**

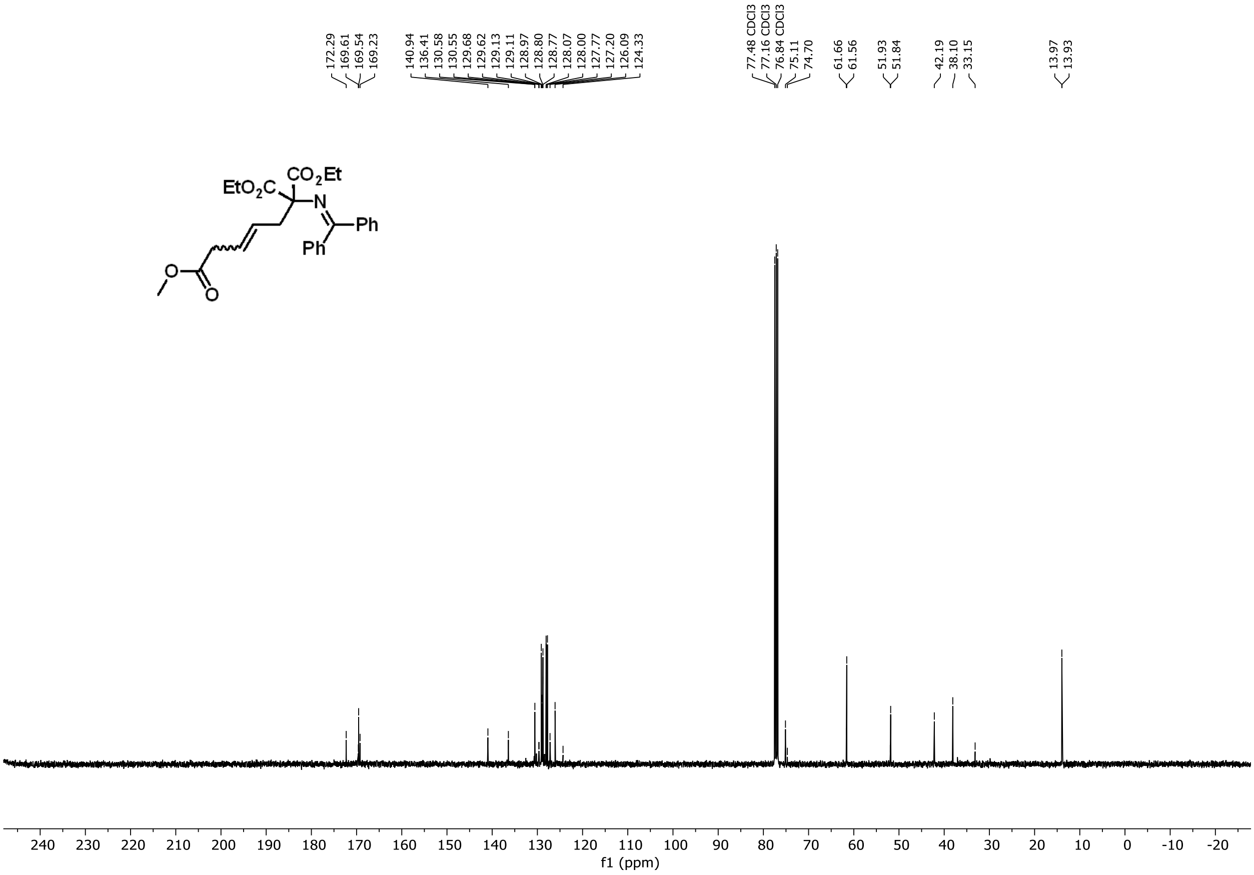

^{1}H NMR (400 MHz, $CDCl_3$) for **E3**

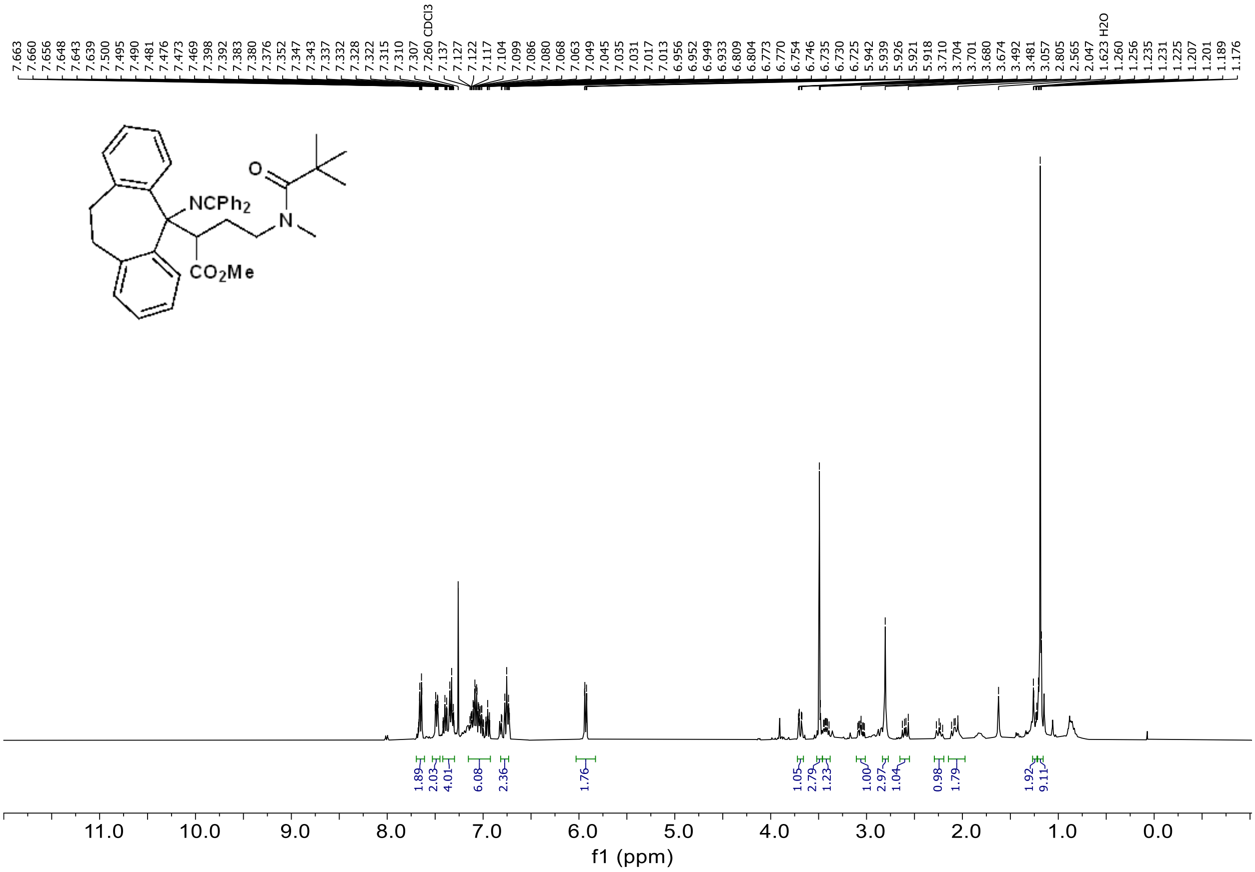

^{13}C NMR (101 MHz, $CDCl_3$) for **E3**

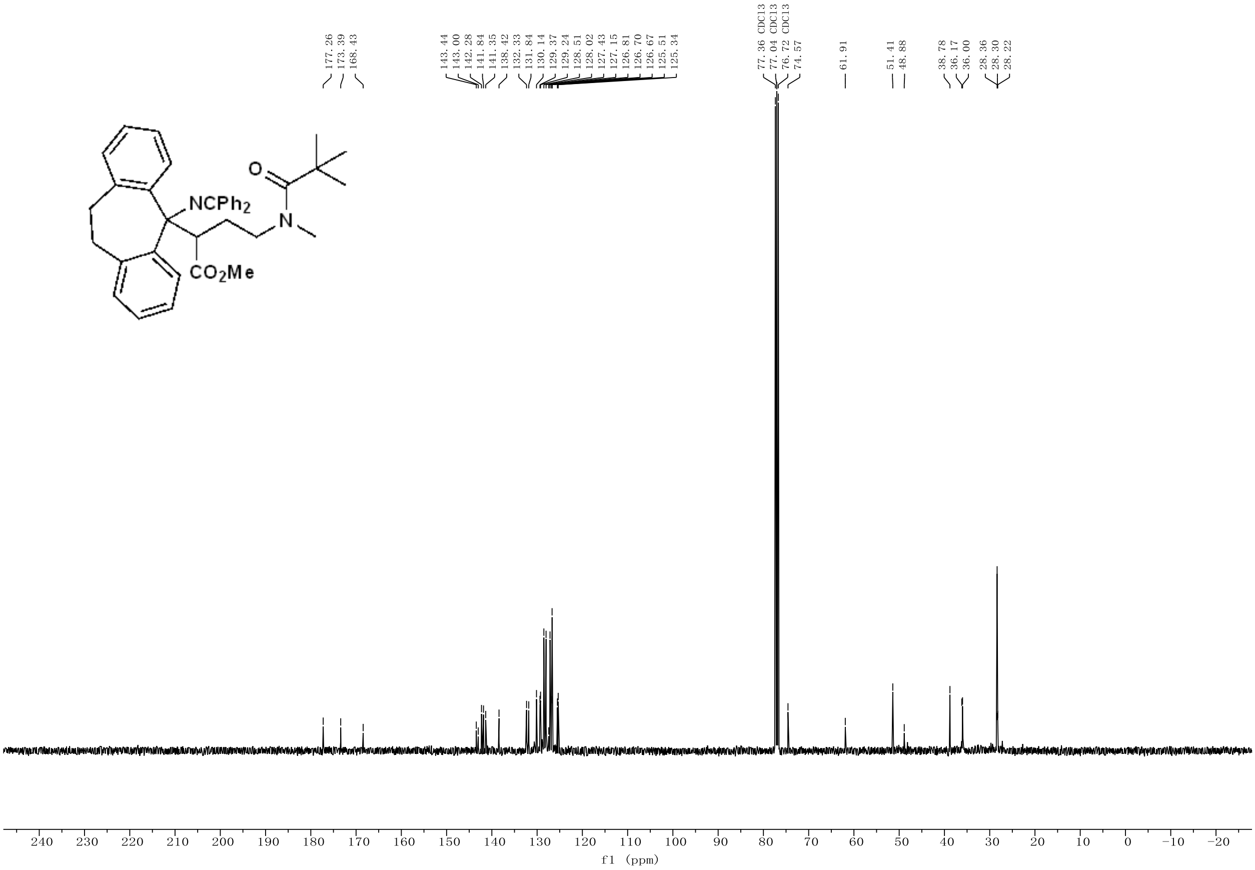

^{1}H NMR (400 MHz, $CDCl_3$) for **K3**

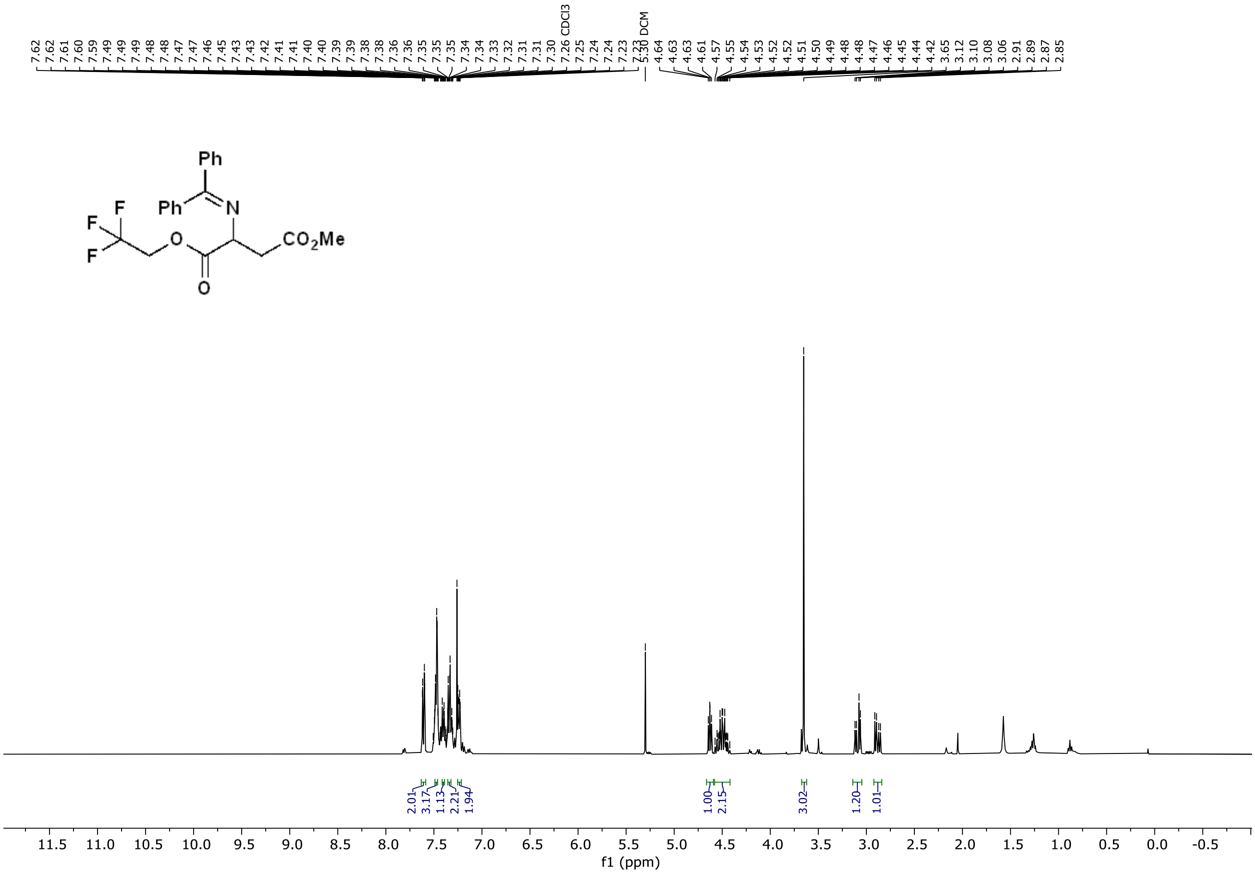

^{13}C NMR (101 MHz, $CDCl_3$) for **K3**

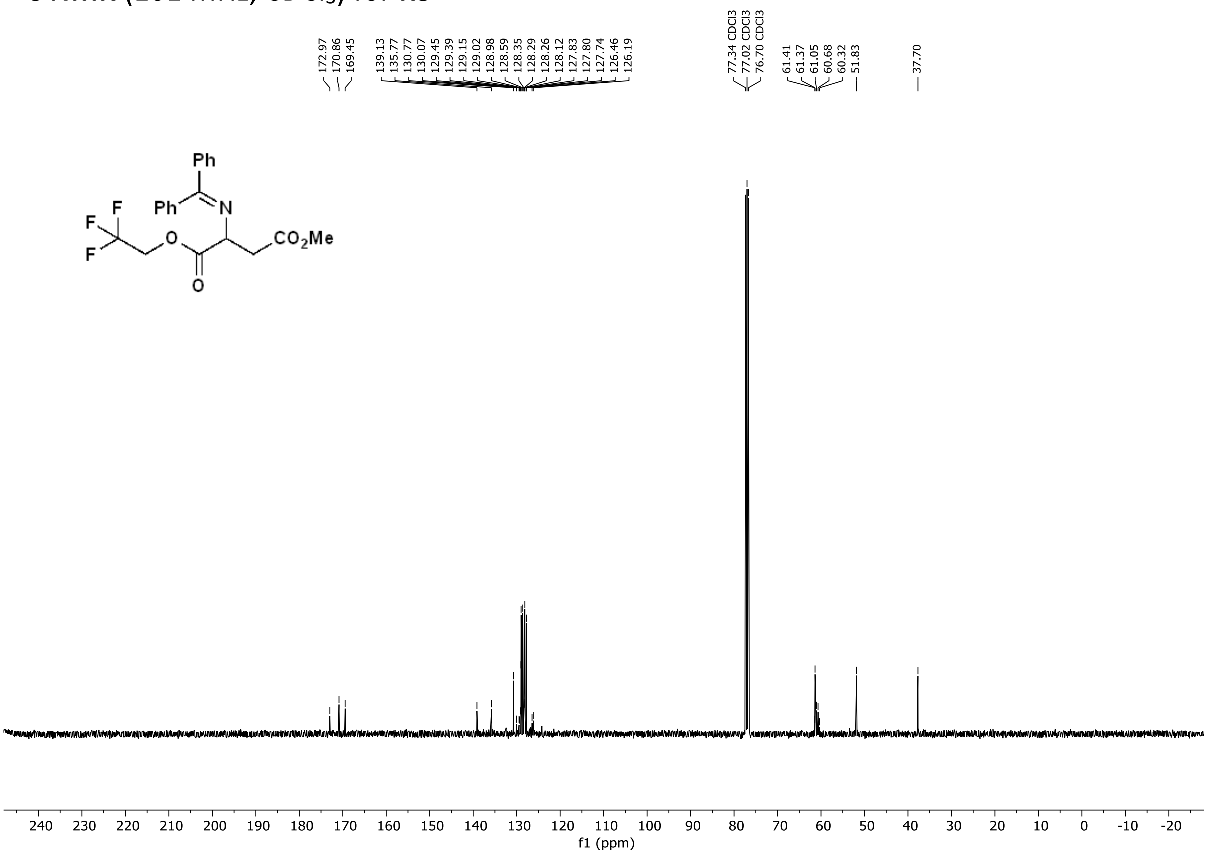

^{19}F NMR (376 MHz, $CDCl_3$) for **K3**

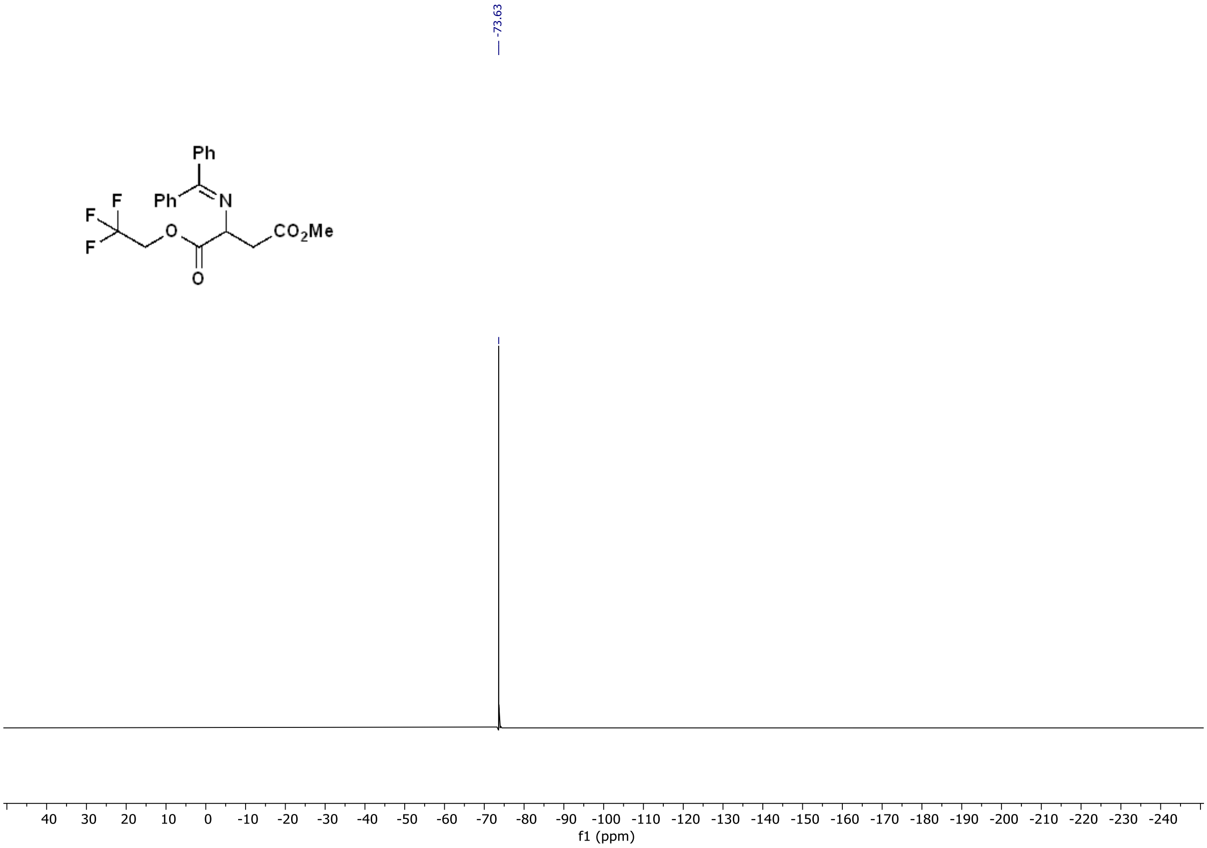

^{1}H NMR (400 MHz, $CDCl_3$) for **L3**

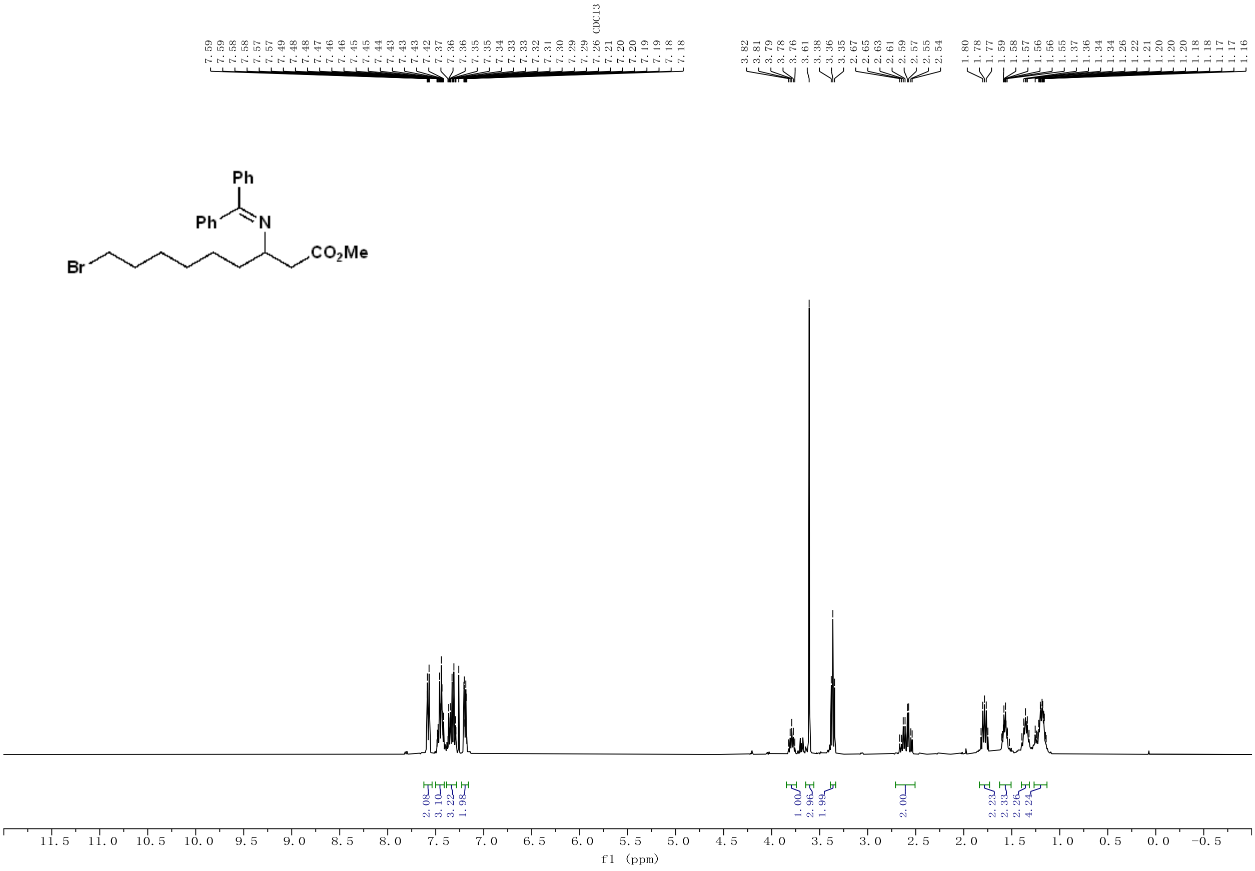

^{13}C NMR (101 MHz, $CDCl_3$) for **L3**

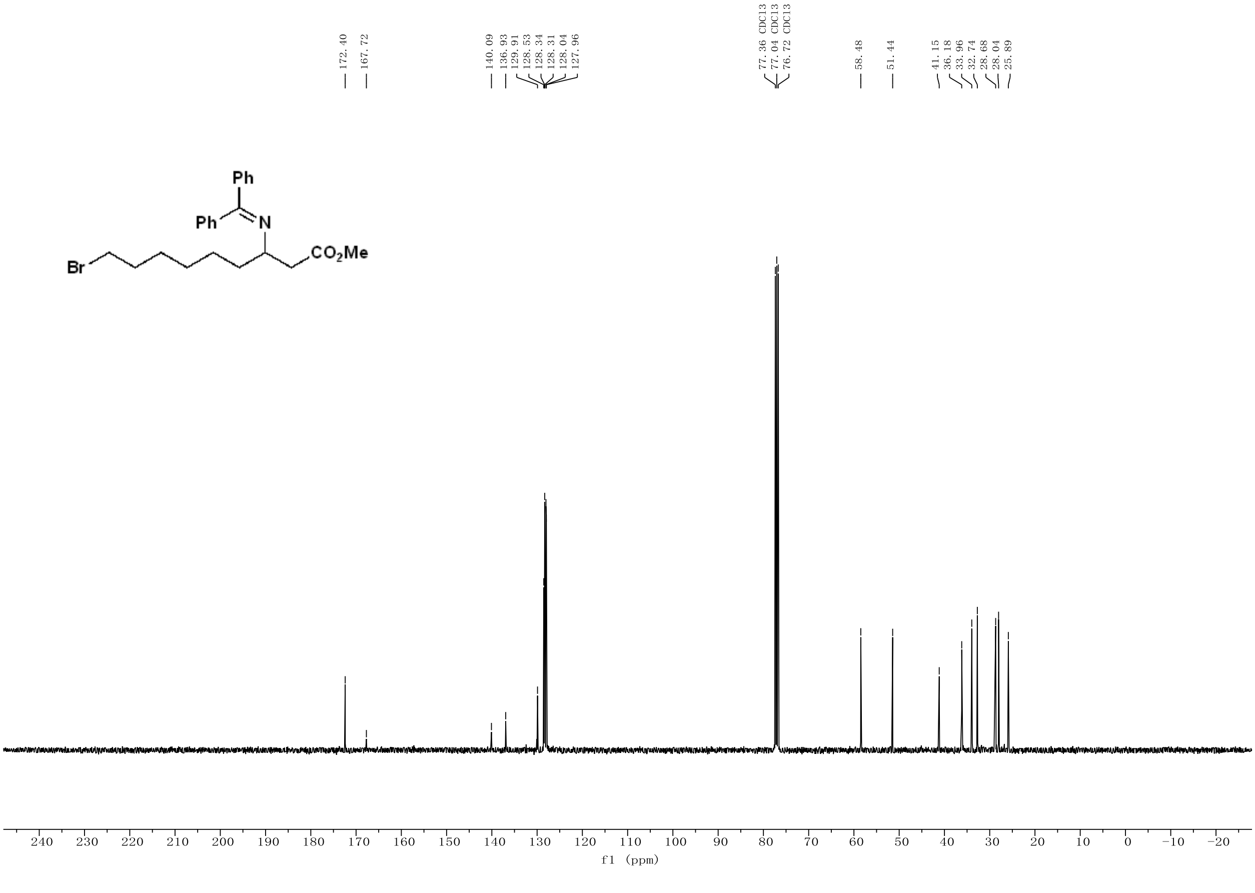

^{1}H NMR (400 MHz, $CDCl_3$) for **M3**

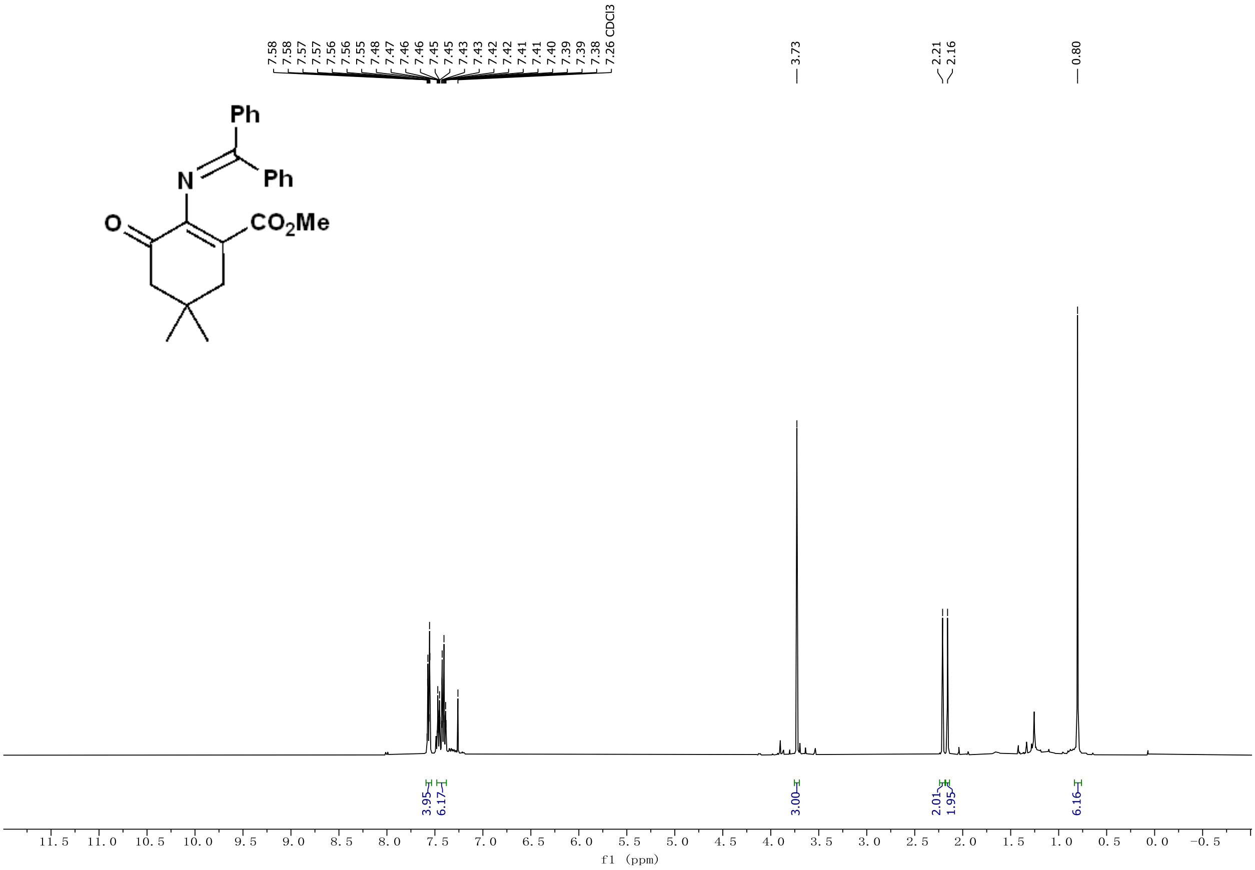

^{13}C NMR (101 MHz, $CDCl_3$) for **M3**

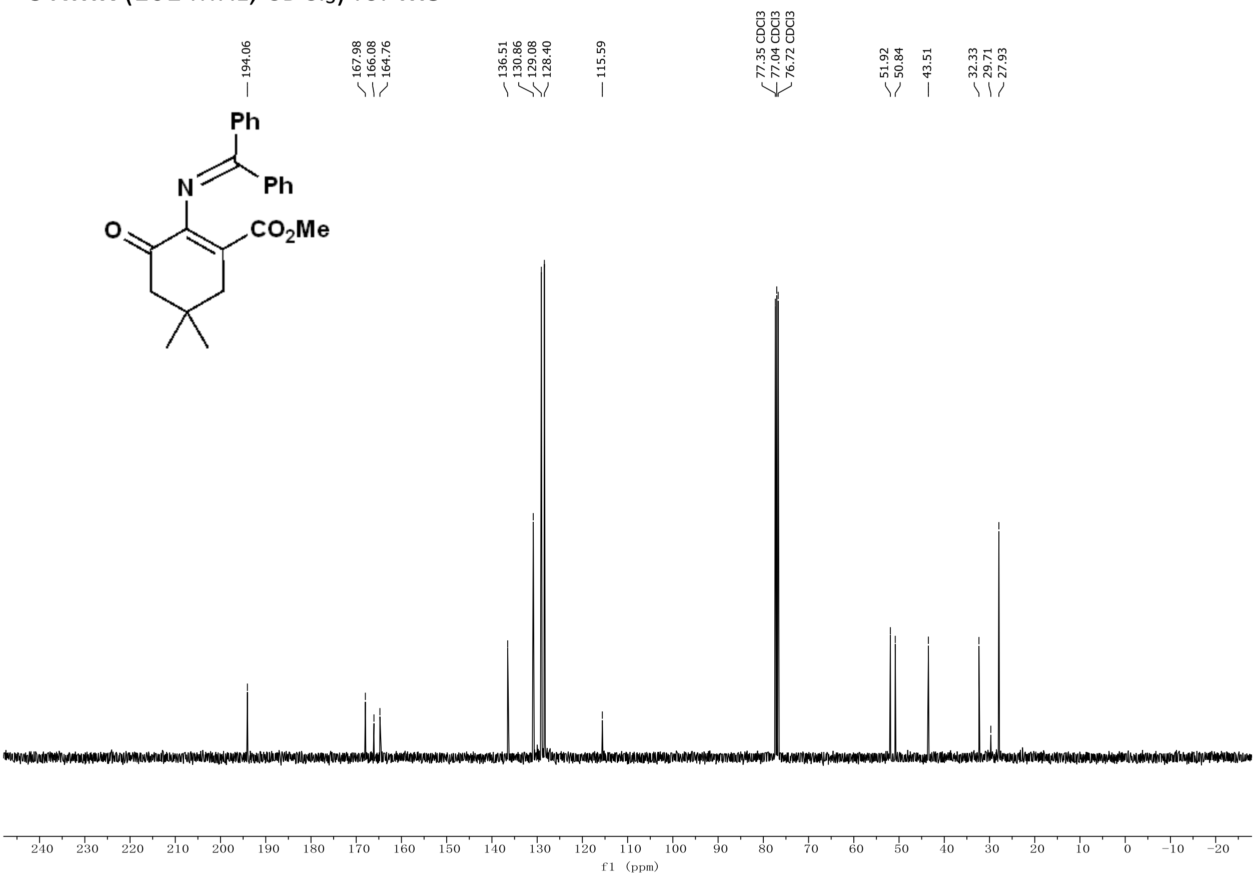

^{1}H NMR (400 MHz, $CDCl_3$) for N3

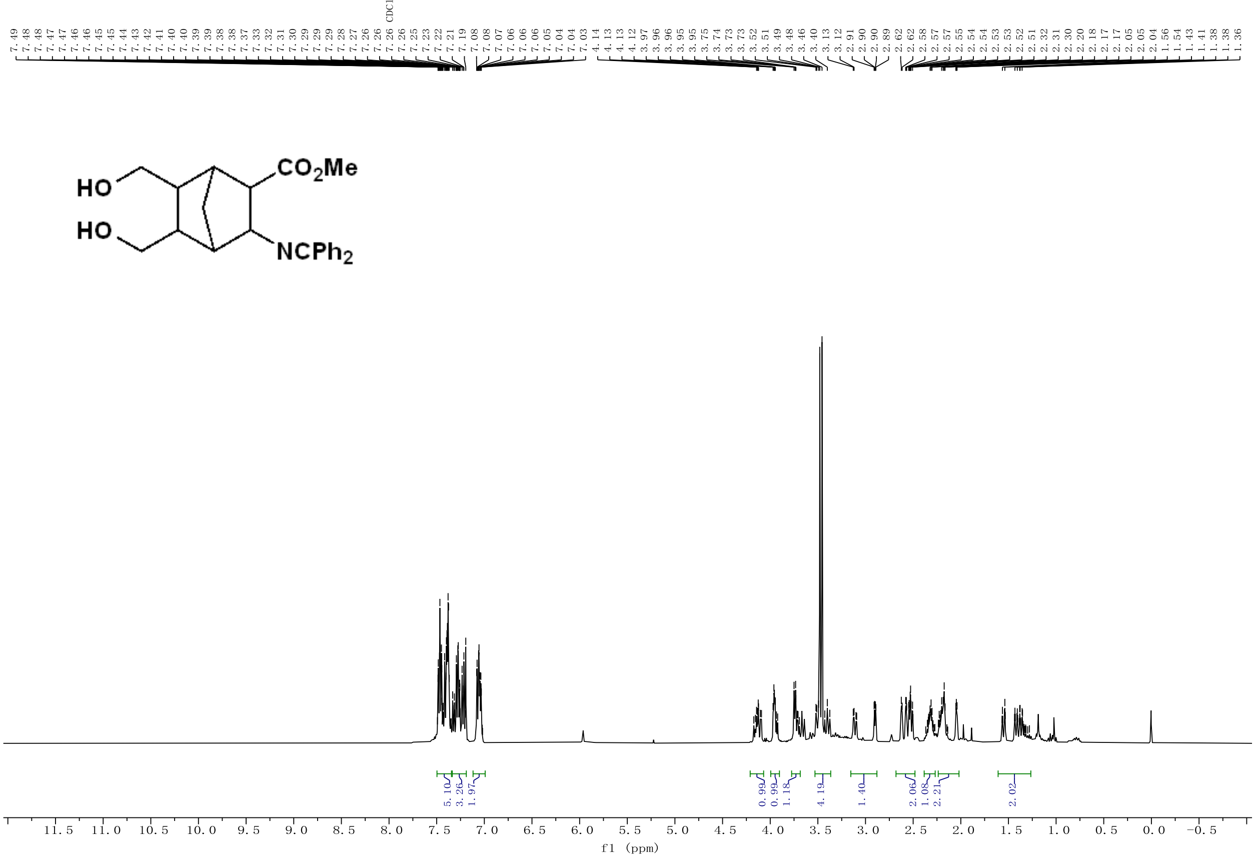

^{13}C NMR (101 MHz, $CDCl_3$) for N3

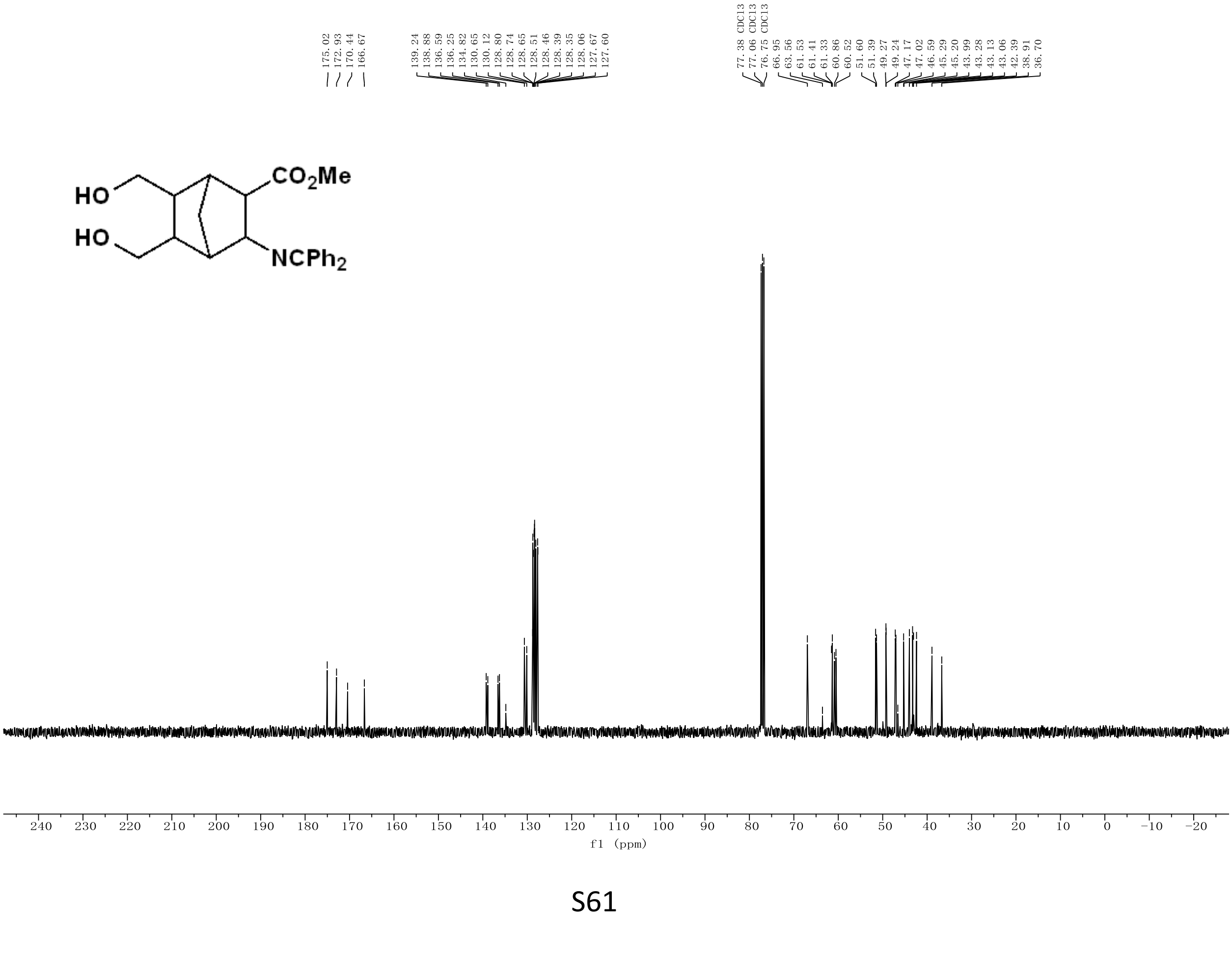

12. References

1. Wishart, D. S. *et al.* DrugBank 5.0: a major update to the DrugBank database for 2018. *Nucleic Acids Res.* **46,** D1074-D1082; 10.1093/nar/gkx1037 (2018).
2. Weininger, D. SMILES, a chemical language and information system. 1. Introduction to methodology and encoding rules. *J. Chem. Inf. Comput. Sci.* **28,** 31–36; 10.1021/ci00057a005 (1988).
3. Tan, G. *et al.* Photochemical single-step synthesis of β-amino acid derivatives from alkenes and (hetero)arenes. *Nat. Chem.* **14,** 1174–1184; 10.1038/s41557-022-01008-w (2022).
4. Elsevier B.V. Reaxys.
5. Daylight Chemical Information Systems, Inc. SMARTS. Available at https://www.daylight.com/dayhtml/doc/theory/theory.smarts.html.
6. Strieth-Kalthoff, F. *et al.* Machine Learning for Chemical Reactivity: The Importance of Failed Experiments. *Angew. Chem. Int. Ed.* **61,** e202204647; 10.1002/anie.202204647 (2022).
7. Kozlowski, M. C. On the Topic of Substrate Scope. *Org. Lett.* **24,** 7247–7249; 10.1021/acs.orglett.2c03246&ref=pdf (2022).
8. Kariofillis, S. K. *et al.* Using Data Science To Guide Aryl Bromide Substrate Scope Analysis in a Ni/Photoredox-Catalyzed Cross-Coupling with Acetals as Alcohol-Derived Radical Sources. *J. Am. Chem. Soc.* **144,** 1045–1055; 10.1021/jacs.1c12203 (2022).
9. Collins, K. D. & Glorius, F. A robustness screen for the rapid assessment of chemical reactions. *Nat. Chem.* **5,** 597–601; 10.1038/nchem.1669 (2013).
10. Flask. Available at https://flask.palletsprojects.com/en/2.3.x/.
11. McInnes, L., Healy, J. & Melville, J. UMAP: Uniform Manifold Approximation and Projection for Dimension Reduction, 2018.
12. Maggiora, G., Vogt, M., Stumpfe, D. & Bajorath, J. Molecular similarity in medicinal chemistry. *J. Med. Chem.* **57,** 3186–3204; 10.1021/jm401411z (2014).
13. Rousseeuw, P. J. Silhouettes: A graphical aid to the interpretation and validation of cluster analysis. *J. Comput. Appl. Math.* **20,** 53–65; 10.1016/0377-0427(87)90125-7 (1987).
14. Rogers, D. & Hahn, M. Extended-connectivity fingerprints. *J. Chem. Inf. Model.* **50,** 742–754; 10.1021/ci100050t (2010).
15. Durant, J. L., Leland, B. A., Henry, D. R. & Nourse, J. G. Reoptimization of MDL keys for use in drug discovery. *J. Chem. Inf. Comput. Sci.* **42,** 1273–1280; 10.1021/ci010132r (2002).
16. Müllner, D. Modern hierarchical, agglomerative clustering algorithms, 2011.
17. VanRheenen, V., Kelly, R. C. & Cha, D. Y. An improved catalytic OsO4 oxidation of olefins to -1,2-glycols using tertiary amine oxides as the oxidant. *Tetrahedron Lett.* **17,** 1973–1976; 10.1016/S0040-4039(00)78093-2 (1976).

18. Schroeder, M. Osmium tetraoxide cis hydroxylation of unsaturated substrates. *Chem. Rev.* **80,** 187–213; 10.1021/cr60324a003 (1980).

19. Kolb, H. C., VanNieuwenhze, M. S. & Sharpless, K. B. Catalytic Asymmetric Dihydroxylation. *Chem. Rev.* **94,** 2483–2547; 10.1021/cr00032a009 (1994).

20. Jacobsen, E. N., Marko, I., Mungall, W. S., Schroeder, G. & Sharpless, K. B. Asymmetric dihydroxylation via ligand-accelerated catalysis. *J. Am. Chem. Soc.* **110,** 1968–1970; 10.1021/ja00214a053 (1988).
